# Widespread declines of formerly common species reshape local assemblages over time

**DOI:** 10.1038/s41467-026-77501-4

**Published:** 2026-09-12

**Authors:** Weiqiang Xu, Junfeng Gao, Qiang Su, Xiaorui Lv, Jonathan M. Chase

**Affiliations:** 1 College of Earth and Planetary Sciences, University of Chinese Academy of Sciences, Beijing, China; 2 Institute of Botany, Chinese Academy of Sciences, Beijing, China; 3 College of Resources and Environment, University of Chinese Academy of Sciences, Beijing, China; 4 German Centre for Integrative Biodiversity Research (iDiv) Halle-Jena-Leipzig, Leipzig, Germany; 5 Department of Computer Science, Martin-Luther-University Halle-Wittenberg, Halle (Saale), Germany

**Keywords:** Biodiversity, Macroecology, Biogeography

## Abstract

Despite dramatic human-driven changes across ecosystems, local-scale aggregate biodiversity metrics are often surprisingly stable, even as communities experience substantial reorganization of species identities and relative abundances. While rare species are often thought to be more vulnerable to these changes, emerging evidence suggests that common species may experience disproportionately greater declines. To evaluate these alternatives, we synthesize population trends for species initially rare or common across assemblages from 4748 sites within 129 studies spanning terrestrial, freshwater, and marine ecosystems. We quantify how population trends relate to initial abundances using hierarchical Bayesian models that account for regression to the mean and include realm, taxon, and environmental covariates. We find consistently stronger declines among formerly common species in marine and terrestrial assemblages, while freshwater assemblages show no clear directional pattern. Rare species present at the start also decline, but less so than common species, while colonists that were not present at the start of the time series compensate for losses within the rarest abundance category. Climate change and anthropogenic drivers modulate these trends, with increasing temperature and non-climatic human pressures attenuating losses of initially common species, particularly in marine systems. Overall, we find widespread declines in common species through time, with potential functional consequences.

## Abstract (web summary)

Human-driven environmental change is reshaping ecological communities worldwide. Analysing 4,748 sites across 129 studies, we show that formerly common species decline disproportionately compared to rare species in marine and terrestrial assemblages.

## Introduction

Rapid biodiversity change is a defining feature of the Anthropocene^1^. Across ecosystems, temporal changes in species richness, total abundance, and species composition have become central metrics for tracking the state of biodiversity and the potential influence of anthropogenic drivers, such as climate change and land use^2–4^. Yet debates persist about the direction and magnitude of these changes^5,6^. Some syntheses suggest that most measures of biodiversity show consistent declines across many taxa^7,8^, while others report relatively stable patterns, especially at local scales^3,9^. However, summary metrics like abundance or species richness can mask substantial reorganization within communities, where declines in some species are offset by increases in others^3,10,11^. Likewise, total abundance and other summary variables may remain stable even as species identities shift, populations fluctuate, or dominance hierarchies shift^9,12^. Importantly, accounting for bias, uncertainty, and spatial representation can strongly influence how we infer population and community-level patterns^13–15^.

Within a given ecological assemblage, the total abundance of individuals can provide an indicator that reflects ongoing change within that assemblage. Total abundance can decline if some anthropogenic driver consistently and negatively influences all taxa within that assemblage. For example, estimates of vertebrates in general^16^, birds in many parts of the world^17,18^, and terrestrial insects^19,20^ often show a general declining trend across entire assemblages. On the other hand, total abundances can increase, for example, when anthropogenic pressures are alleviated^19^, when environmental conditions (including temperature) are enhanced^21^, and/or when disturbances suppress competitors or predators, allowing other, often smaller species to be released and increase in total abundance^22,23^.

Changes in total abundance within an assemblage, however, need not uniformly reflect changes in the abundances of the taxa within that assemblage^24–26^. Prominent indicators, such as the Living Planet Index^16^ and related analyses^25,27^, emphasize trends in selected populations, but have a number of potential biases (e.g., taxonomic focus on vertebrates^15,28^) that complicate inference about how biodiversity change is distributed across species ranks within an assemblage. Regardless of whether overall abundances in an assemblage are static, decreasing or increasing, conservation concerns have traditionally emphasized that rare species are most vulnerable to changing conditions, owing to their small populations and often narrow environmental tolerances^29^. Some assemblage-level studies support this, showing steeper declines in rare species, especially where environmental filtering or habitat loss prevails^30^. Occupancy studies likewise suggest that widespread species often expand while narrowly distributed species contract their ranges through time^31,32^, but whether this translates to abundance patterns remains unclear. In contrast, some evidence from assemblages of birds^7,17,33^, insects^12,34^ and plants^35^ suggests that, even after accounting for sampling biases and statistical artifacts such as the regression to the mean^36,37^, species that were initially more common in the assemblage at the start of the time series may often experience disproportionate declines compared to those that were initially rarer. To date, there appears to be little consistent directionality to these trends; some studies report steeper declines in species that were initially more common, while others report greater proportional decline in initially rarer species or no systematic differences (Supplementary Data 1).

We evaluate species-time series level population trends using a global compilation of community time series from taxa spanning terrestrial, freshwater, and marine ecosystems from the BioTIME 2.0 database^38^ in order to determine whether there are general patterns in the directionality of population trends among species that were initially more common or rare in the assemblage. From the BioTIME 2.0 database^38^, we select time series from assemblages with at least 10 sampled years (mean = 15.9 years) from across 129 studies and 4,748 sites (1,225 from terrestrial, 66 from freshwater ecosystems, and 3,457 from marine systems; Supplementary Fig. 1). We first assign species to assemblage level abundance classes at the start of the time series, and we apply a regression-to-the-mean correction to mitigate bias in trend estimates related to initial abundance. This correction is necessary because species with high or low extreme initial abundances can show artifactual trends due to random fluctuations, independent of any real directional change. We estimate the expected regression-to-the-mean effect from the longest time series in our dataset and subtract this from the full trend estimates (see Methods), shrinking results toward zero and reducing bias in each realm separately^12^. We then fit a hierarchical Bayesian autoregressive model to estimate species-level abundance trends over time, including realm and year (with their interaction) as fixed effects, and random intercepts for study, taxonomic group, time series, and species-time series combinations. We implement random slopes at all hierarchical levels to capture differential temporal responses across them. The model incorporates temporal autocorrelation through an autoregressive structure with random effects nested within each time series. To assess the role of environmental drivers, we model abundance trends as a function of temperature change^39,40^ and other non-climatic anthropogenic drivers (e.g., land transformation, resource exploitation, pollution, and invasive species)^41^. For each response, the model yields a trend slope estimate of change and associated 80%, 90%, and 95% credible intervals (CRI). We classify trends as strong evidence when the 95% CRI does not overlap zero, moderate when the 90% CRI excludes zero, and weak when the 80% CRI excludes zero^12^.

## Results and discussion

### Disproportionate declines of common species across realms

**Fig. 1:**
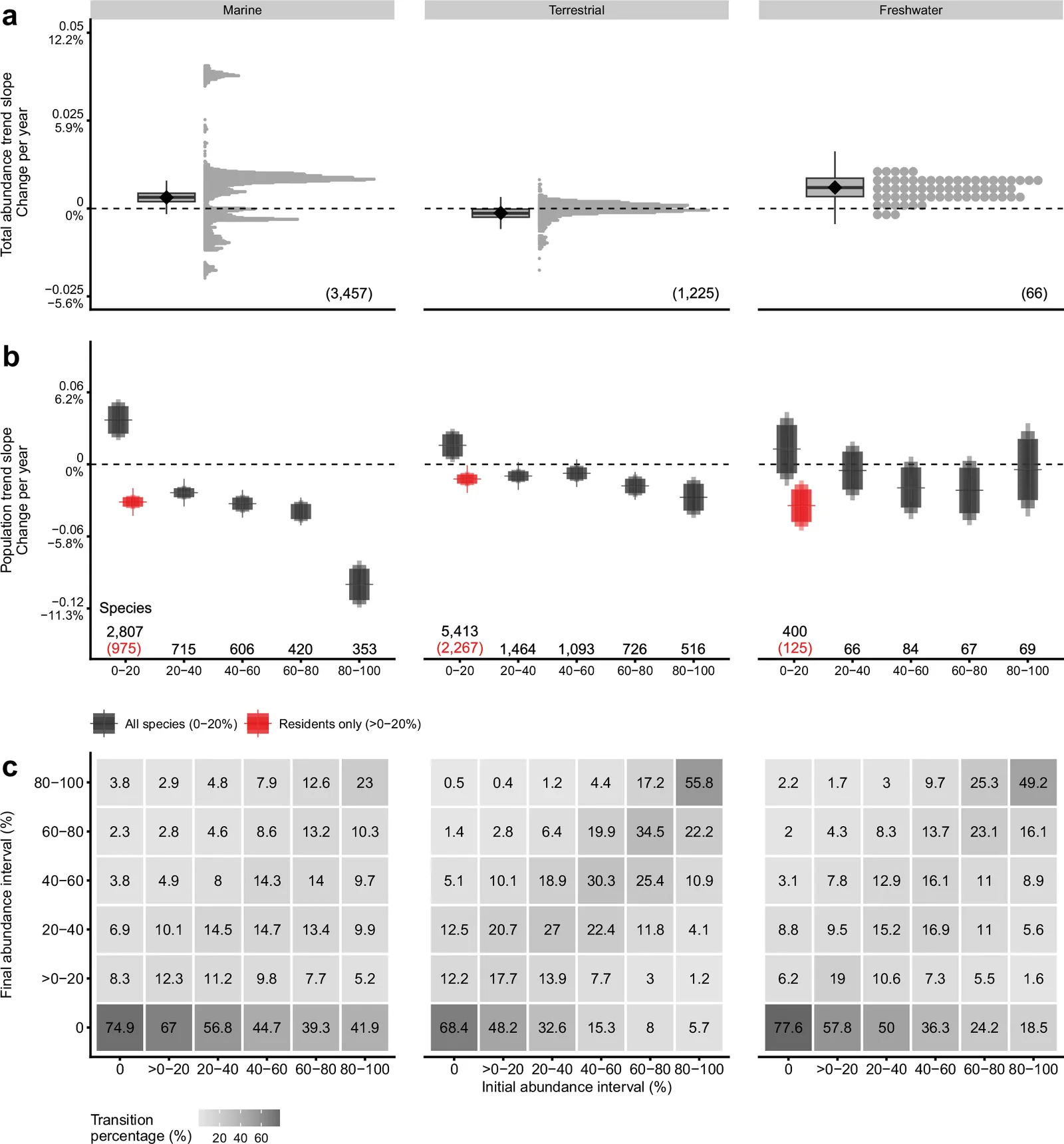
Total abundance change and contributions across species abundance distributions. **a** Distribution of total abundance change across all assemblages. Each point represents one time series; boxplots show the median (horizontal line), the interquartile range (box bounds), and values within 1.5× the IQR (whiskers), with filled diamonds indicating means. Numbers in parentheses indicate the total number of independent assemblage time series per realm (marine: *n* = 3457; terrestrial: *n* = 1225; freshwater: *n* = 66). **b** Population trend estimates per initial abundance interval, corrected for regression-to-the-mean effects. Cross symbols indicate posterior means; thick, medium, and thin error bars represent 80%, 90%, and 95% credible intervals, respectively, from 4000 posterior samples of fixed effects. Grey symbols include all species in the 0–20% interval (including colonists); red symbols show estimates for initial residents only (species with non-zero abundance in the first survey year, excluding colonists). Numbers indicate the number of species per interval, parenthetical values for the 0–20% class show the resident-only subset. **c** Abundance bin transition matrices across realms. Each cell shows the percentage of species transitioning from an initial (*x*-axis) to a final (*y*-axis) abundance interval, based on log_10_-transformed abundance in the first and last survey years. Darker shading indicates higher transition percentages. The diagonal represents species that retained their initial abundance rank. The zero column shows species absent at the start of the time series that colonized later (colonists), and the zero row shows species present at the start that were absent by the end (local extinctions).

In marine assemblages, we found moderate evidence that total abundance increased through time (0.73% annually, 90% CRI: 0.04, 1.41%), despite considerable heterogeneity among studies. In particular, we observed more negative abundance trends from studies that included multiple taxa (Supplementary Fig. 2), suggesting that heterogeneous trends among individual taxa may miss broader patterns observed among several taxa. While increases in abundance in marine systems may seem antithetical to the decline narrative from marine systems, such increases in total abundance are consistent with documented losses of top predators and associated trophic releases of abundant prey^23,42^ as well as compositional shifts toward smaller-bodied species^43,44^. In freshwater, we also found weak evidence of an overall increase in total abundance (1.38%, 80% CRI: 0.22, 2.56%), some of which may be attributed to water quality improvements, increasing nutrient inputs, climate change, or invasion of nonnative species^19,45,46^. Finally, in terrestrial assemblages, we found no directional trends in total abundance (Fig. 1a and Supplementary Data 2) despite global estimates of population declines in several terrestrial taxa^16,18,19,25,27^.

We next examined how groups of species from different parts of the relative abundance spectrum changed through time. Both marine and terrestrial systems showed differences in population change rates depending on initial abundance, while freshwater showed no such variation, though the limited number of freshwater studies (*n* = 66 time series) may have constrained our ability to detect patterns. In both marine and terrestrial systems, species initially in the most abundant category (80–100%) showed the strongest declines (marine: –9.51%, 95% CRI: –11.23, –7.70%; terrestrial: –2.70%, 95% CRI: –4.35, –1.03%), with intermediate abundance categories also declining but less steeply (Fig. 1b and Supplementary Data 3).

When we compared trends of species initially in the rarest category (0–20%), we considered two conceptually distinct definitions of this group that are often confounded. The first included all species occurring at any point during the time series that were initially low in abundance, encompassing both species that were present but rare at the start, and species absent initially that either colonized later or were too rare to detect (initial residents + colonists). The second included only species that were present but rare at the very start of the time series, excluding species that colonized later. Species that were colonists only were excluded because their trends can only increase from zero or return to zero if they subsequently go extinct.

When both residents (initial abundance >0) and colonists (initial abundance = 0) were included, trends in this rarest category were positive for both marine (3.76%, 95% CRI: 2.05, 5.56%) and terrestrial realms (1.60%, 95% CRI: 0.19, 3.00%). However, this effect was primarily driven by colonizing species whose numbers started at zero and then increased. When we removed colonists and considered only initial residents, the rarest species also declined, albeit less steeply than those in the most abundant categories for both marine (–3.08%, 95% CRI: –3.65, –2.51%) and terrestrial (–1.21%, 95% CRI: –1.80, –0.62%) systems. This is consistent with an independent analysis of a subset of assemblages from the same dataset that found increasing rarity through time driven primarily by immigration rather than recovery of resident rare populations^47^.

To evaluate the sensitivity of our results to the choice of abundance interval classification, we repeated analyses using quartile-based abundance intervals rather than equal log_10_-spaced bins, finding qualitatively identical results (Supplementary Fig. 3). To address sensitivity to different starting times, we also defined initial abundance as the mean over the first three and five years rather than the first year alone, and again found qualitatively similar results (Supplementary Fig. 4). Finally, to quantify and control for regression-to-the-mean bias, which could produce artifactual trends where initially common species appear to decline and initially rare species appear to increase, we truncated time series by one and three years and used the difference in slope estimates as realm-specific correction factors^12^. Although this correction reduced the magnitude of estimated trends, the direction and relative ordering across abundance classes remained consistent (Supplementary Fig. 5 and Supplementary Data 4).

Another way to visualize how species change in relative abundance through time is to examine how species in different initial abundance categories transition to other abundance categories from the start to the end of their time series, including new colonists that entered the assemblage after the first observations and species that went locally extinct before the final observations were taken (Fig. 1c). An important observation is that in each realm, approximately 70–80% of colonizing species that were not present in the first observations were highly transient, going locally extinct before the final observation (Fig. 1c). This is consistent with the frequent observation that species assemblages tend to be characterized by a suite of core species that are consistently present, and transient species that frequently come and go^48,49^. In addition, we would expect that many transient species that may be present at the start of the time series will not be observed at the end, and their subsequent loss will contribute to an overall decline in initially rare species that cannot be disentangled from losses that may result from other ecological processes (e.g., habitat deterioration, species interactions).

Local extinction rates of species that were initially rare in the time series were consistently higher than those of species that were more common in all systems, as might be often expected from both probability and ecological processes^11,50–52^. In addition, in terrestrial and freshwater assemblages, about 50% of the species present in the most common category at the start of the time series remained in this category at the end. In marine systems, however, only about 23% of species that were initially more common in the time series remained so at the end, with most moving to lower abundance categories. The pronounced loss of initially abundant marine species may reflect a combination of commercial fishing targeting removal of the most common species^53^ and climate-driven range shifts that are particularly rapid in marine systems^54,55^.

### Variation among taxa

**Fig. 2:**
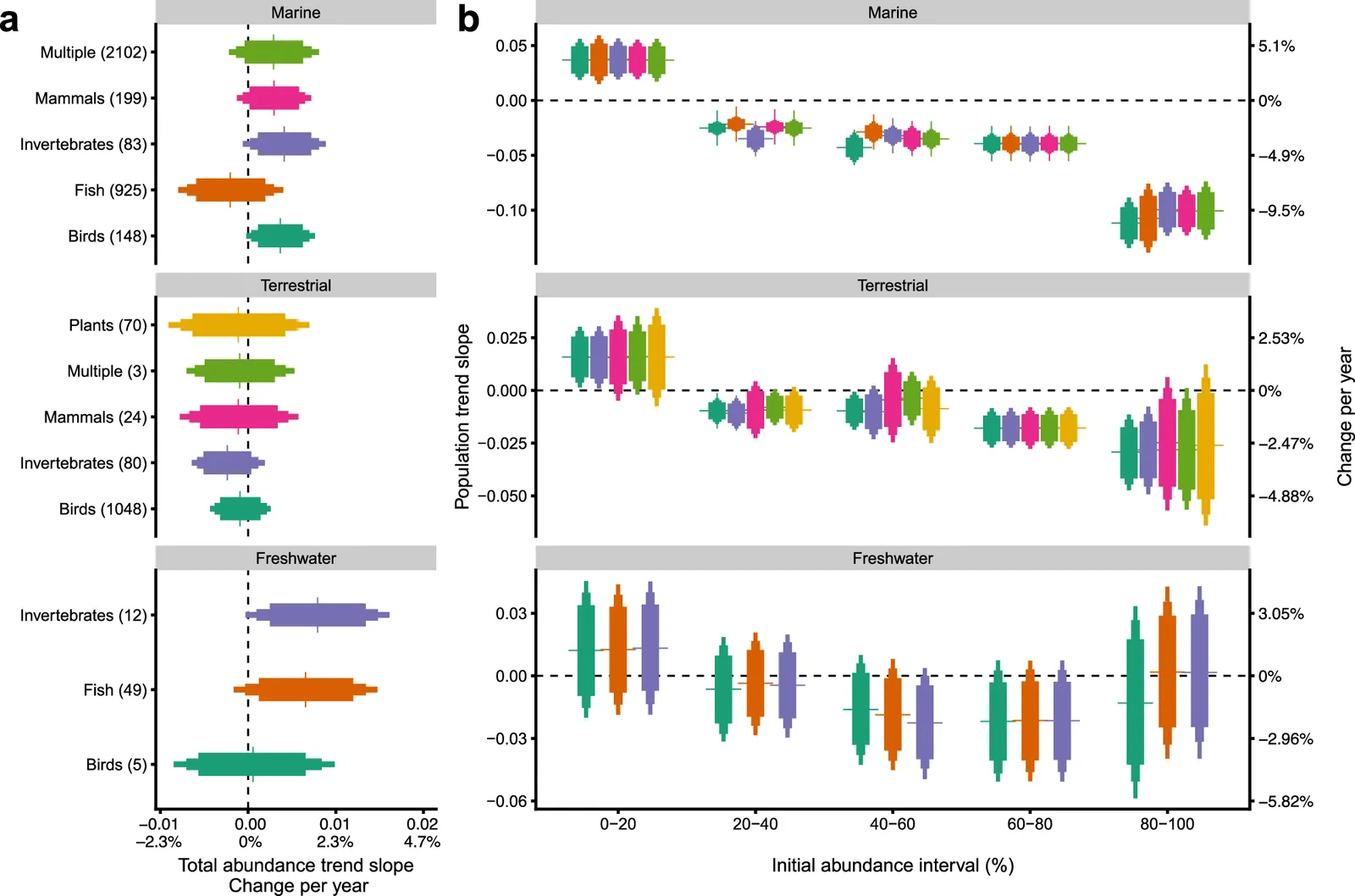
Taxonomic patterns of abundance change across realms and abundance ranks. **a** Trend slope of total abundance change across taxonomic groups. Cross symbols indicate posterior means; thick, medium, and thin error bars represent 80%, 90%, and 95% credible intervals, respectively. Numbers in parentheses indicate assemblage time series per taxonomic group within each realm. Colors identify taxonomic groups as labeled on the *y*-axis. Marine assemblages show predominantly positive trends; terrestrial and freshwater systems show no significant changes for most taxa. **b** Population trends across taxonomic groups and realms in relation to initial relative abundance rank. Cross symbols indicate posterior means from taxon-level random effects; thick, medium, and thin error bars represent 80%, 90%, and 95% credible intervals, respectively. Sample sizes (number of species) per taxonomic group, realm, and abundance interval are provided in Supplementary Data 6. Colors correspond to taxonomic groups as in (**a**). Common species consistently decline more than rarer species across marine and terrestrial ecosystems.

Patterns of total abundance change varied by taxa and realm (Fig. 2a and Supplementary Data 5). In marine systems, there was moderate evidence for increases in invertebrate abundance (0.95%, 90% CRI: 0.05, 1.88%), possibly because of trophic restructuring following predator declines^23^. There was also moderate evidence for increases in marine birds (0.85%, 90% CRI: 0.08, 1.62%) and weak evidence for increases in marine mammals (0.68%, 80% CRI: 0.04, 1.35%), both potentially as a result of conservation recoveries in some assemblages^53^. Marine fish abundances did not change across the time series, possibly due to the opposing effects of overexploitation and trophic release. In terrestrial systems, we found no clear trends for any taxa (Fig. 2a), which is surprising given previous evidence for declines in terrestrial insects^12,19^ and birds^7^, including many time series that were also included in our synthesis. We have no clear explanation for this discrepancy, except that the database we used contains more heterogeneous data than some of the more iconic studies. Finally, despite wide credible intervals and limited sample size, there was moderate evidence of increases in total abundances of invertebrates (1.84%, 90% CRI: 0.22, 3.47%) in freshwater systems, which is consistent with previous syntheses^19,56^. Likewise, we found weak evidence of increases in the abundance of freshwater fishes (1.52%, 80% CRI: 0.28, 2.80%; Fig. 2a), which is consistent with syntheses from warm, but not cold, streams^46^.

Despite these differences in total abundance trends among taxa, we found similar shifts in the strength of declines among species in different abundance categories for most taxa in marine and terrestrial realms (Fig. 2b and Supplementary Data 6). That is, most taxa that were in the most abundant category declined steeply through time, while species with intermediate abundances declined more slowly. For all taxa in these realms, temporal changes in species that were categorized in the initially rarest category increased through time when colonizing species were included, but still declined when only initially resident rare species were considered. Nevertheless, as with the general pattern, the decline in initially rare species was much less than that of species in the more abundant category for most taxa in marine and terrestrial realms (Fig. 2b). Freshwater taxa again showed no clear increasing or decreasing patterns regardless of their initial abundance class.

From a conservation perspective, the fact that initially rarer species tended to experience weaker declines or increases may seem encouraging yet somewhat counterintuitive, given their presumed vulnerability to change. However, there are several reasons why more common species could initially be more likely to decline than rare species. For example, if species living in their more favorable conditions were historically more common species in an assemblage (as predicted by the abundance-center hypothesis^57,58^), directional environmental change could disproportionately impact them by shifting to conditions that are less favorable and lead to steeper declines in previously abundant species. This process systematically undermines the conditions that historically enabled more common species to be abundant. Likewise, many common species are particularly vulnerable to overharvesting by humans^50^ and/or from natural enemies such as herbivores or disease^59,60^, which would tend to lead to their disproportionate decline. Indeed, there are many examples where once hyper-abundant species have gone extinct or near extinct, which can have profound consequences for ecosystem functioning^50,61^. Rare species in a given assemblage, by contrast, may persist at the ecological margins where their local abundances are governed more by dispersal limitation, environmental constraints, or biotic interactions that buffer them from environmental changes^50^.

These patterns of internal reorganization within communities likely underlie the high species turnover that is consistently observed across biodiversity time series, with typically static biodiversity trends^2,3,5,54^. We found that temporal trends in biodiversity are largely absent in the terrestrial and freshwater systems we synthesized, whereas in marine systems, we found strong evidence of increasing species richness through time (0.75%, 95% CRI: 0.32, 1.16%; Supplementary Fig. 6), consistent with a previous synthesis on a smaller dataset^62^. Because we found increasing trends in both total abundance and species richness, but little evidence for changes in diversity measures that include relative abundances (e.g., Hill numbers; Supplementary Fig. 6), we infer that the patterns of increasing species richness in marine systems primarily resulted from a sampling effect associated with increases in the numbers of individuals.

### Community-level declines amplify common species losses

**Fig. 3:**
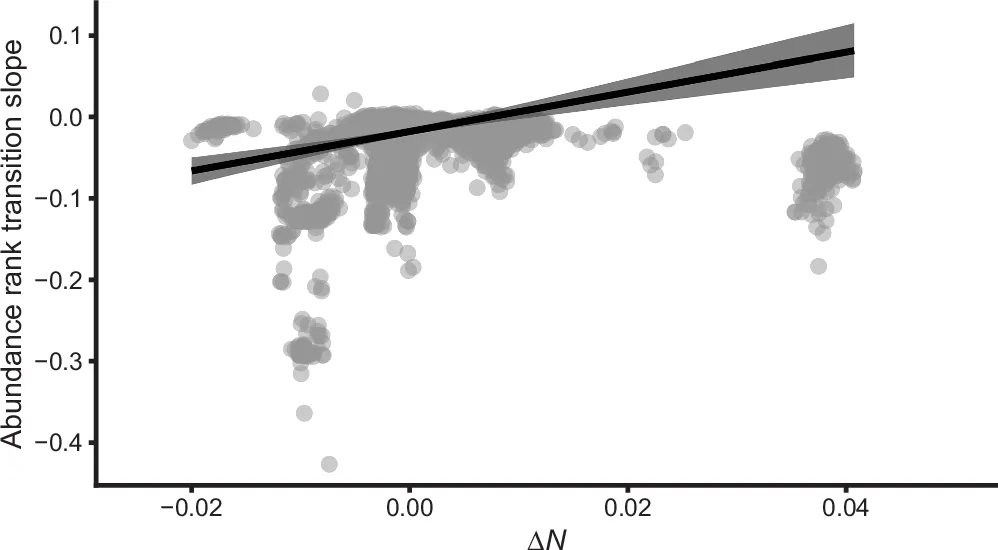
Relationship between community-level abundance change and disproportionate species losses. Each point represents an ecological assemblage (*n* = 4501), showing the abundance rank transition slope (*y*-axis; more negative = stronger disproportionate loss of initially common species) against net change in total community abundance (Δ*N*, *x*-axis). The fitted line shows the posterior mean fit (slope = 2.436, 95% CRI: 1.618, 3.255), with shading indicating the 95% credible interval from the hierarchical Bayesian model, indicating that communities experiencing net declines exhibit steeper negative trend-abundance associations, while those experiencing net gains show positive but less pronounced associations. This suggests that shifts in species trends are not uniform, and that the magnitude of these species-time series level changes collectively determines whether the total assemblage gains or loses individuals.

We next evaluated whether the observed shifts in species’ population trends depended on overall change in the total abundance (Δ*N*) of the assemblage through time. For each assemblage, we quantified the linear association between a species’ population trend and its log_10_-transformed initial abundance, which we refer to as the abundance rank transition slope. Assemblages with more negative abundance-rank transition slopes exhibited stronger disproportionate losses of the initially most common species. Across assemblages, we found a clear positive relationship between the abundance rank transition slope and the assemblage-level abundance trend (*β* = 2.436, 95% CRI: 1.618, 3.255; Fig. 3). In communities that were losing total abundance of individuals overall, we found that species that were formerly more abundant declined disproportionately more than initially rarer species. This is consistent with the clear trends of strong losses in both the total abundance and the abundance of the most common species observed in North American and European bird communities^7,17,33^, as well as terrestrial insect communities^12,34^. In contrast, assemblages that were either stable or increasing in total abundance over time showed weaker-to-positive trend-abundance associations (though less pronounced than the negative associations observed in declining communities). This asymmetry implies that common species may be especially vulnerable to losses in shrinking communities, which might be expected, for example, in the face of environmental change that disrupts the conditions favored by species that previously were able to become more common, or in the face of overexploitation by people or natural enemies^50,61^.

### Environmental drivers modulate abundance trends

To determine to what degree patterns of the trends we observed might be associated with potential drivers of change, we first determined whether trends in the changes of total assemblage abundance were influenced by (1) changing temperatures at the site over the course of the observation period, or (2) the degree to which non-climatic anthropogenic drivers influence that site (Supplementary Fig. 7). We found that sites that experienced higher levels of temperature change were associated with more positive abundance trends in both marine and terrestrial systems, while freshwater assemblages showed no consistent relationship (likely owing to too few studies in this realm). In contrast, non-climatic anthropogenic drivers were positively associated with abundance trends in marine systems, possibly because these drivers can disrupt food webs and can indirectly lead to release of smaller, more abundant, species^23^. However, non-climatic anthropogenic drivers were slightly negatively associated with abundance trends in terrestrial systems, perhaps because land-use change and other pressures can directly influence habitat availability. However, because our data are disproportionately drawn from western and northern countries where ecosystems are often highly modified (Supplementary Fig. 1), their recovery trajectories may differ from less-sampled regions. The positive relationships in marine systems could plausibly reflect recent population recoveries following historical declines, recoveries that co-occur with ongoing warming and human modification (Supplementary Fig. 7), rather than direct positive responses to contemporary change. These time series are subject to a shifting baseline, where the most severe impacts likely took place before monitoring started.

**Fig. 4:**
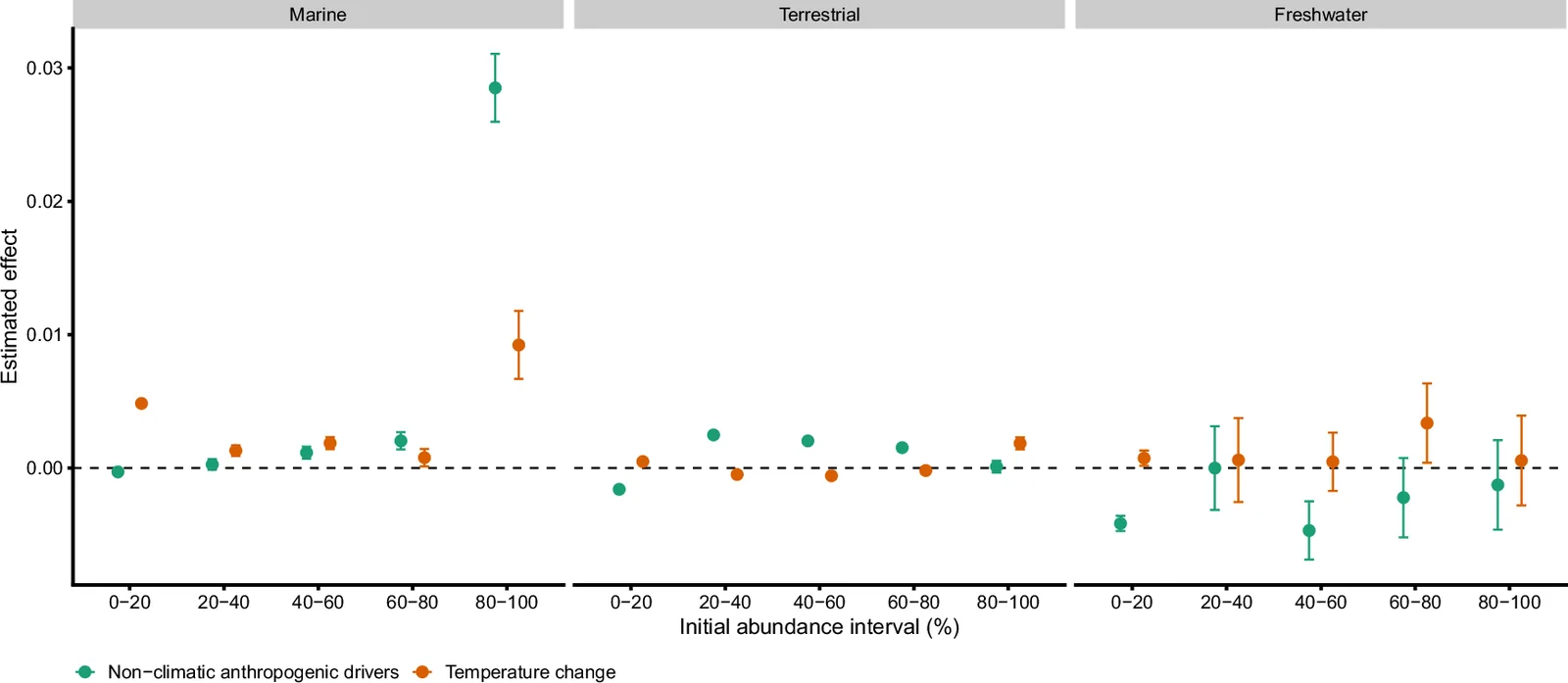
Effects of temperature change and non-climatic anthropogenic drivers on abundance trends across rank intervals. Standardized OLS regression coefficients relating environmental variables to species-level abundance change rates grouped by initial abundance interval. Points represent coefficient estimates; error bars show 95% confidence intervals. Burnt-orange points indicate temperature change; teal-green points indicate non-climatic anthropogenic drivers. Results are presented separately for marine, terrestrial, and freshwater realms. Sample sizes (number of species) per realm and abundance interval are provided in Supplementary Data 7.

We next evaluated whether these environmental drivers could explain the observed tendency for formerly common species to decline more than rare ones. To do so, we tested whether temperature change or non-climatic anthropogenic drivers predicted species-time series level abundance trends across initial abundance classes (Fig. 4 and Supplementary Data 7). In marine systems, both climatic warming and other anthropogenic drivers were most strongly associated with attenuated declines in the most common species, while rarer species showed weaker or negative responses. This suggests that warming may attenuate the decline of common marine species or amplify gains in rarer ones, consistent with climate-driven redistributions and biomass turnover in marine ecosystems^54,55^. Non-climatic anthropogenic drivers had a weak negative effect on the rarest species but increasingly positive associations for common species, suggesting that these pressures may stabilize more common species while suppressing gains in rarer ones. That is, both of these drivers appear to push against the main pattern we observed of disproportionate losses of initially more common species. In terrestrial systems, the effects did not show a clear, consistent pattern. In freshwater systems, non-climatic anthropogenic drivers were associated with attenuated gains in rarer species. However, the small sample size (*n* = 66) and wide confidence intervals across most abundance classes limit the strength of broader conclusions for this realm. Collectively, these results suggest that while both climatic and non-climatic changes can shape species trajectories, neither fully accounts for the broader pattern we found, in which initially common species declined disproportionately over time. Instead, this pattern may also reflect the influence of additional drivers that are incompletely captured by currently available indices or quantified at spatial scales too coarse to resolve local variation, and which may act independently or synergistically with climate and land-use change. Environmental drivers may help determine which species initially become common or rare, but they do not appear to be the primary cause of species' population trajectories across different abundance classes.

Although our findings reveal a consistent pattern of systematic declines in the population trajectories of species that were formerly common across marine and terrestrial realms and taxa, several uncertainties remain. First, our results here are based on data on local abundances, and definitions of initial commonness and rarity come from local assemblages from a snapshot at the start of the time series. Our assemblage-specific abundance classification means that species with similar absolute abundances could be categorized differently across assemblages, depending on local dominance hierarchies. This focus on within-assemblage relative abundance is central to our question, but it is clear that other definitions can address related aspects of rarity and biodiversity change^51,52^. For example, rarity can be quantified by spatial distributions (i.e., broad versus narrow geographic ranges), and there is evidence that more narrowly distributed species may be more vulnerable to anthropogenic changes^32,63^. The discrepancy between results from rarity defined as distributional range and rarity defined by local abundance may arise because the often expected positive relationship between local abundance and regional occupancy^64^ is weaker when there is spatial variation in habitat quality^65^. Instead, many species that are regionally rare in their distributions (i.e., habitat specialists) can be quite common locally in their favored habitats, which can buffer them from local extinctions so long as those habitats remain intact^66^.

We also must clearly state several limitations of our analyses. Foremost, despite the considerable expansion of the BioTIME 2.0 database in geographical and taxonomic scope^38^, the data are clearly underrepresented in tropical and other poorly sampled regions like all global syntheses, and also have other potential biases^14,67^. For example, long-time series data can come only from those assemblages that were continually monitored through time, which might be biased towards those systems that are less strongly impacted by anthropogenic drivers. Likewise, the data are disproportionately drawn from western and northern countries (Supplementary Fig. 1), where ecosystems are often highly modified and where monitoring intensity, management histories, and recovery trajectories may differ from less-sampled regions. Finally, we acknowledge potential limitations of community-level time series that largely span the past 50 years, given the shifting baselines these communities have experienced long after much anthropogenic change had already occurred. Collectively, these limitations mean that our estimates characterize the assemblages represented in our sample, and should not be taken as statistically representative of the full global population of local assemblages across taxa and ecosystems. Resolving these uncertainties will require both better characterization of the global representativeness of existing time-series data with respect to environmental gradients^14^, and integrating long-term time series with spatial monitoring frameworks that track biodiversity change across gradients of habitat change^68^.

Beyond these representational issues, variation in sampling site fidelity (e.g., fixed plots vs. variable trawls) could introduce noise from spatial heterogeneity in monitoring. While such variation could introduce noise rather than capture genuine temporal change, we analyzed data using only high-fidelity studies (those with explicitly fixed sampling locations) and found qualitatively similar results (Methods; Supplementary Fig. 8). Finally, our main models did not explicitly account for phylogenetic relatedness among species or residual spatial autocorrelation among time series. While we do not expect these omissions to substantially alter the direction of our trend estimates, they may lead to underestimated uncertainty in the credible intervals, as has been shown with part of the dataset we used^13^. However, we performed a sensitivity analysis that incorporated both a phylogenetic correlation matrix and a spatial autocorrelation structure within the high-fidelity subset of data, and found patterns largely consistent with the overall result that common species are declining disproportionately faster than rare species in marine and terrestrial realms, although credible intervals were wider with reduced strength of some evidence from individual estimates (Supplementary Fig. 9). Nonetheless, readers should interpret the strength of evidence for individual trend estimates in the full-dataset results with appropriate caution.

The primary results from our study highlight a fundamental tension in how biodiversity change unfolds. Most efforts in conservation biology have focused on the vulnerability of rare species to ongoing anthropogenic changes and the need to mitigate these losses through targeted conservation and restoration strategies. However, there has been growing evidence that it is often the common and dominant species that are disproportionately declining^7,12,17,50^. Our global synthesis of community assemblage time series shows that this is a predominant phenomenon across many taxa, ecological realms, and assemblages. We further show that this decay of more common species is more abrupt in systems that are showing overall decreases in their total abundances. These formerly common species are those that shape biodiversity and ecosystems through sheer abundance, driving energy flow, nutrient cycling, and ecological interactions, and their decline may leave ecosystems less predictable, even if the fate of rarer species is more variable^50,61,69^.

## Methods

### Data collection and selection

We started with the BioTIME 2.0 database^38^, which compiled 708 studies (Study IDs) encompassing 1,603,067 sampling events, across 553,253 locations, leading to a total of 11,989,233 records from 56,400 species from across marine, terrestrial, and freshwater realms. To obtain reliable abundance trend estimates of taxa from different parts of the distribution from common to rare species, we applied the following data filtering criteria: (1) time series with at least 10 samped years; (2) abundance measurements recorded as integers; (3) studies with species-time series level identification; (4) reptiles (*n* = 2 time series) and amphibians (*n* = 1 time series) were reclassified into the “Multiple” taxonomic group category due to limited data availability (where “Multiple” refers to Study IDs that collected multiple taxonomic groups); additionally, we removed multiple-taxa data from the freshwater realm (*n* = 1 time series) and marine plant data (*n* = 1 time series), as well as time series where species abundance remained constant at 1 throughout the entire period (*n* = 56 time series; likely real observations but excluded as constant singleton abundances prevent meaningful calculation of relative abundance distributions and classification into abundance intervals). To address convergence issues in subsequent Negative Binomial models and ensure data quality, we further excluded time series containing extreme abundance values (any data point exceeding the 99.5th percentile of ~67,000 individuals, primarily affecting marine plankton studies, *n* = 520 time series) and time series with potentially erroneous geographic coordinates (*n* = 4 time series). For studies with multiple sampling locations spanning large spatial areas, we used an approach described in previous work^3,38^ to generate unique time series identifiers (Study ID + grid cells) based on geographic coordinates using regular hexagonal grids of 96 km^2^. Prior to estimating abundance trends and calculating diversity metrics, we used a sample-based rarefaction procedure with 199 iterations for each time series, using the minimum sample size across all years as the standardized sampling effort. In each iteration, every year was independently and randomly subsampled to this minimum, with all subsampling confined to samples from the same study and the same 96-km^2^ grid cell. The median value of each biodiversity metric across iterations was recorded, which minimizes the influence of anomalous samples and helps average out any within-cell spatial heterogeneity arising from independent random draws across years. After filtering, our final dataset comprised 4748 time series from 129 studies, including birds, invertebrates, fish, mammals, plants, and one multiple-taxa group. The temporal coverage spans from 1910 to 2023, encompassing marine (3457), terrestrial (1225), and freshwater (66) realms.

### Species abundance data processing

For each of the 199 rarefied samples, we calculated total abundance, species richness (*q* = 0), Hill number for Shannon (*q* = 1) and Simpson (*q* = 2), and Pielou’s evenness, and then recorded the median value of each metric across iterations. In our species-level time-series population trend analyses, we first calculated the median abundance for each species from rarefied data. We then log_10_-transformed each species’ first-year abundance and created five equal-width intervals (0–20%, 20–40%, 40–60%, 60–80%, and 80–100%) between 0 and the maximum transformed value within each assemblage. Each species was assigned to the appropriate interval according to its transformed first-year abundance. We used first-year abundance rather than maximum abundance across the time series to avoid circularity, as species with early abundance peaks would otherwise be disproportionately classified as common and show artifactual declines. To test robustness to the binning scheme, we repeated all trend analyses using an alternative division based on quartiles of initial abundances (see Sensitivity analyses). In addition, species absent in the first year but detected subsequently may represent colonization events or previously undetected extremely rare residents. To include the full rarity continuum and avoid biasing estimates by exclusion, we assigned these species an initial abundance of zero and placed them in the lowest class. All diversity metrics were calculated using the vegan R package^70^.

To control for regression-to-the-mean (RtM) effects on population trend estimates, we implemented a correction-factor methodology. The RtM effect can produce artificial trends for species with extreme initial abundances—species with very high initial abundances may show exaggerated declines due to random fluctuations, while those with very low initial abundances may exhibit exaggerated increases^36,37^. Our approach consisted of the following steps (following ref. ^12^): we selected time series with at least 20 sampling time-points to model each initial abundance group; we then left-truncated the data by one year (and three years as a sensitivity test, Supplementary Fig. 5 and Supplementary Data 4), and re-estimated population trends using the truncated time series; finally, we calculated the difference in slopes between complete and truncated time series as a correction factor, with separate factors calculated for each realm (all taxonomic groups within a realm shared the same correction factor). This factor was added to the estimates from the complete analysis, thereby shrinking the final results toward zero and mitigating the influence of regression-to-the-mean effects.

### Species-time series level trend estimation

To quantify population trends across species of varying initial abundances, we employed an autoregressive hierarchical Bayesian modeling approach. Species abundance was modeled using a Negative Binomial distribution to appropriately handle count data with overdispersion. We also fitted Poisson models as an alternative, but adopted the Negative Binomial framework for all main results based on substantially lower Watanabe Akaike Information Criterion (WAIC) values (Supplementary Data 8). The model included time (year), ecological realm, and their interaction as fixed effects.

We addressed data non-independence and temporal dependencies using a nested random effects structure, incorporating random intercepts for Study IDs, assemblages, taxonomic group, and species. To capture differential temporal responses across these hierarchical levels, we specified corresponding random slopes. We further subdivided the taxonomic group into study-realm-taxa combinations (e.g., marine versus terrestrial birds) to account for potential differences when the same taxonomic group appeared across realms. This nested random-effects structure is commonly used to account for the dominant source of spatial clustering in BioTIME (i.e., sampling units grouped within Study IDs and assemblages^71^), thereby reducing pseudo-replication and inflated effective sample sizes. However, we acknowledge that this grouping structure may not fully capture fine-scale spatial dependence, if present. In addition, while population dynamics may exhibit phylogenetic signal that could influence inferred temporal trends^13^, empirical evidence for its influence on abundance-related attributes and responses to environmental change is mixed and strongly context dependent across taxa and spatial scales^13,72,73^, so incorporating phylogeny was not expected to alter our results in a consistent or readily interpretable direction and was therefore not explicitly modelled in the present analysis. However, unmodelled phylogenetic relatedness and residual spatial autocorrelation may lead to underestimated uncertainty in the presented credible intervals; we evaluate this potential consequence in a dedicated sensitivity analysis (see Sensitivity analyses; Supplementary Fig. 9). We therefore caution that credible intervals reported throughout this study should be interpreted with this limitation in mind, and acknowledge that fully incorporating phylogenetic and spatial correlation structures in population trend analyses remains an important direction for future work.

We explicitly modelled temporal autocorrelation using an autoregressive term of order 1 (AR1) at the time series level. We implemented this model using Integrated Nested Laplace Approximation (INLA)^74^, which efficiently approximates Bayesian posterior distributions while accommodating complex hierarchical structures. The model used penalized complexity priors with hyperparameters set to reflect one standard deviation of the data (*α* = 0.01). The complete model specification was:

$${Abun}{dance} \sim {Realm}\times {Year}+(1|{Stud}{y}_{{ID}})+(1|{Taxon})+(1|{Assemblage})$$

$$+(1|{Assemblag}{e}_{{Species}})+(0+{Year}|{Stud}{y}_{{ID}})+(0+{Year}|{Taxon})$$

$$+(0+{Year}|{Assemblage})+(0+{Year}|{Assemblag}{e}_{{Species}})$$

$$+{AR}1({Year}|{Assemblage})$$

where $(1{|\cdot })$ denotes random intercepts, $(0\,+{Year|\cdot })$ denotes random slopes for year effects, and ${AR}1({Year|Assemblage})$ specifies the temporal autocorrelation structure nested within each assemblage.

For community-level diversity metrics (total abundance, species richness, Hill numbers and Pielou’s evenness), we applied a similar hierarchical framework but removed species-time series level random effects. All diversity metrics were log_10_-transformed and analyzed using Gaussian distributions, except Pielou’s evenness, which was modeled using a beta distribution with logit link function given its bounded nature (0–1).

### Abundance bin transition analysis

To quantify within-assemblage reorganization across abundance intervals over time, we constructed realm-specific transition matrices comparing each species’ bin assignment between the start and end of each time series. For each assemblage, species abundances were extracted separately from the first and last survey years. Within each year, abundances were log_10_-transformed, and each species’ transformed abundance was normalized by the maximum log_10_-transformed abundance recorded across all species in that assemblage in the same year; this normalization was performed independently for the first and last survey years, so that bin assignments reflect each species’ relative position within the assemblage hierarchy at each respective time point. Each species was then assigned to one of six abundance intervals based on this normalized score: 0 (absent), >0–20%, 20–40%, 40–60%, 60–80%, and 80–100%. The five non-zero intervals follow the same equal-width classification scheme used in the main population trend analysis; however, we additionally separated zero-abundance observations into a distinct interval to distinguish species absent in a given survey year that later colonized the assemblage (colonists) from those that were present but rare (initial residents), as these two groups differ fundamentally in expected trend direction. We then calculated, separately for each realm, the percentage of species–assemblage combinations transitioning from each initial interval to each final interval. Results are presented as realm-specific heatmaps (Fig. 1c), with initial abundance intervals on the *x*-axis, final abundance intervals on the *y*-axis, and cell values denoting transition percentages.

### Scaling of trends with initial abundance

To examine whether the pattern of declining common species varied with overall community abundance dynamics, we quantified the relationship between abundance rank transition slope and total abundance change. For each assemblage, we calculated an abundance rank transition slope by regressing species’ population trends against their initial abundance (log_10_-transformed) using ordinary least squares (OLS). This slope quantifies the strength of the abundance-trend relationship, with more negative values indicating steeper disproportionate losses of the initially most common species. We then analyzed how these slope values related to the corresponding assemblage-level abundance trends (Δ*N*) using a hierarchical Bayesian model with the same structure as our earlier analyses of community-level diversity metrics. This approach allowed us to test whether the abundance rank transition slope is a consistent phenomenon across assemblages or varies systematically with overall abundance trajectories. We excluded assemblages where species had identical abundance ranks (*n* = 247), which prevented meaningful regression fitting, resulting in 4,501 assemblages for this analysis. The relationship was visualized with standardized regression coefficients and 95% credible intervals to assess the strength of evidence (Fig. 3).

### Environmental driver analysis

To assess relationships between the external environment—specifically, temperature change and an index of non-climatic anthropogenic drivers—and the relative abundance change rates, we used estimated abundance change slopes at the time-series level as response variables. We constructed separate models for total abundance changes (Supplementary Fig. 7) and for changes within each initial abundance interval (Fig. 4). Using OLS, we independently analyzed each realm (marine, terrestrial, freshwater) with standardized temperature change slopes and non-climatic anthropogenic driver indices as explanatory variables. We evaluated the impact of environmental drivers by visualizing standardized regression coefficients and their 95% confidence intervals.

We obtained temperature data from CRU TS (terrestrial, 0.5° spatial resolution)^40^ and HadISST (marine, 1° spatial resolution)^39^. For each time series, we extracted monthly mean temperature records from the start year to the end year of the observation period (Year_start_: Year_end_), converted them to annual averages, and estimated annual temperature change rates using OLS. We sourced the non-climatic anthropogenic driver index from ref. ^41^, which integrates data on climate change, land transformation, resource exploitation, population density, pollution, and invasive species introduction to comprehensively quantify human modification of terrestrial, freshwater and marine landscapes through multiple pathways. To avoid double-counting with climate change variables, we excluded climate change-related metrics from this index.

### Sensitivity analyses

We conducted four sensitivity analyses to assess the robustness of our population trend estimates to methodological choices regarding abundance interval classification, initial abundance definitions, sampling site fidelity, and unmodelled correlation structures.

1. Quartile-based binning: To determine whether the choice of abundance interval classification had any qualitative influence on our results, we examined the effect of using quartile-based rather than equal-width bins. To do so, we ranked all species within each realm by their log_10_-transformed abundance in the initial survey year and assigned them to four quartile intervals, each containing approximately equal numbers of species–assemblage combinations. Species not detected in the first year but recorded later in the time series were included in the lowest quartile alongside the rarest detected species. We then re-ran our standard hierarchical model separately for each quartile interval (Supplementary Fig. 3).
2. Multi-year baseline for initial abundance definition: To determine whether using only the first survey year to classify species into abundance intervals had any qualitative influence on our results, we examined the effect of using a multi-year baseline to reduce the influence of year-to-year stochastic variation. To do so, we calculated each species’ initial abundance as the mean of its log_10_-transformed abundances over the first three (or five) survey years of each assemblage time series. Species were then classified into five equal-width abundance intervals based on these multi-year baseline values, and trend estimates were re-calculated for each interval (Supplementary Fig. 4).
3. Sampling site fidelity: To determine whether inconsistent sampling locations had any qualitative influence on our results, we examined the effect of restricting analyses to studies with confirmed fixed sampling sites. To do so, we classified all 129 studies into three fidelity levels based on study metadata: high fidelity (studies with explicit permanent or fixed sampling locations; *n* = 76 studies, 59% of total), medium fidelity (studies with consistent sampling domains but without explicit permanent markers), and low fidelity (studies with variable or moving sampling locations; Supplementary Data 9). We then re-ran population trend analyses using only the high-fidelity subset (Supplementary Fig. 8).
4. Phylogenetic and spatial autocorrelation: To determine whether unmodelled phylogenetic relatedness and spatial autocorrelation had any qualitative influence on our uncertainty estimates, we examined the effect of explicitly incorporating these correlation structures into the model. To do so, we added a phylogenetic correlation matrix and a spatial autocorrelation term to the hierarchical Bayesian model, following the approach of ref. ^13^. This analysis was restricted to the high-fidelity subset of studies described in (3) above (Supplementary Fig. 9).

### Reporting summary

Further information on research design is available in the Nature Portfolio Reporting Summary linked to this article.

## Supplementary information

Supplementary Information

Description of Additional Supplementary Information

Supplementary Data 1

Supplementary Data 2

Supplementary Data 3

Supplementary Data 4

Supplementary Data 5

Supplementary Data 6

Supplementary Data 7

Supplementary Data 8

Supplementary Data 9

Reporting Summary

Transparent Peer Review file

## Data Availability

Biodiversity time-series data were obtained from the BioTIME 2.0 database (ref. ^38^), which is publicly available via Zenodo at https://doi.org/10.5281/zenodo.10932823 or the BioTIME 2.0 website (http://biotime.st-andrews.ac.uk/). Terrestrial temperature records (CRU TS v4) are available from the Climatic Research Unit, University of East Anglia (https://crudata.uea.ac.uk/cru/data/hrg/; ref. ^40^). Marine sea surface temperature records (HadISST) are available from the Met Office Hadley Centre (https://www.metoffice.gov.uk/hadobs/hadisst/; ref. ^39^). The non-climatic anthropogenic driver index was obtained from the Supplementary Information of ref. ^41^ (https://doi.org/10.1002/pan3.10071). The processed datasets underlying all figures in this study are available at figshare https://doi.org/10.6084/m9.figshare.29974405. Supplementary Data are provided as Supplementary Data files 1–9.

## Code availability

All analyses were conducted in R version 4.5.0 (ref. ^75^). The R code used to reproduce all analyses and figures is available at figshare https://doi.org/10.6084/m9.figshare.29974405.
