# Supplementary material for: Widespread declines of formerly common species reshape local assemblages over time
Source: Nat Commun. 2026 Sep 12;17:10769. doi: 10.1038/s41467-026-77501-4 (PMC13661514; doi:10.1038/s41467-026-77501-4)
Supplement: Supplementary file 1 [file 41467_2026_77501_MOESM12_ESM.pdf]

nature portfolio

Corresponding author(s): Qiang Su

Last updated by author(s): Aug 5, 2026

Reporting Summary

Nature Portfolio wishes to improve the reproducibility of the work that we publish. This form provides structure for consistency and transparency in reporting. For further information on Nature Portfolio policies, see our Editorial Policies and the Editorial Policy Checklist.

Statistics

For all statistical analyses, confirm that the following items are present in the figure legend, table legend, main text, or Methods section.

n/a	Confirmed	
☐	☒	The exact sample size (n) for each experimental group/condition, given as a discrete number and unit of measurement
☐	☒	A statement on whether measurements were taken from distinct samples or whether the same sample was measured repeatedly
☒	☐	The statistical test(s) used AND whether they are one- or two-sided<br>*Only common tests should be described solely by name; describe more complex techniques in the Methods section.*
☐	☒	A description of all covariates tested
☐	☒	A description of any assumptions or corrections, such as tests of normality and adjustment for multiple comparisons
☐	☒	A full description of the statistical parameters including central tendency (e.g. means) or other basic estimates (e.g. regression coefficient) AND variation (e.g. standard deviation) or associated estimates of uncertainty (e.g. confidence intervals)
☒	☐	For null hypothesis testing, the test statistic (e.g. F, t, r) with confidence intervals, effect sizes, degrees of freedom and P value noted<br>*Give P values as exact values whenever suitable.*
☐	☒	For Bayesian analysis, information on the choice of priors and Markov chain Monte Carlo settings
☐	☒	For hierarchical and complex designs, identification of the appropriate level for tests and full reporting of outcomes
☒	☐	Estimates of effect sizes (e.g. Cohen's d, Pearson's r), indicating how they were calculated

Our web collection on statistics for biologists contains articles on many of the points above.

Software and code

Policy information about availability of computer code

Data collection	No custom software was used for data collection. All data were obtained from publicly available databases and repositories.
Data analysis	All analyses were performed in R v4.5.0 (R Core Team, 2025). Statistical modelling: INLA v25.10.19 (Rue et al., 2009) for hierarchical Bayesian models, vegan v2.8-0 (Oksanen et al., 2025) for diversity metrics, ape, phytools, rotl (phylogenetic analyses; sensitivity analysis only), spdep (spatial autocorrelation; sensitivity analysis only). Data processing: dplyr, tidyr, purrr, BioTIMEr, readr, tibble (tidyverse suite), data.table, reshape2, pbapply, geosphere, raster, ncdf4. Visualisation: ggplot2, cowplot, ggridges, ggdist, broom. Custom analysis code is available at figshare https://doi.org/10.6084/m9.figshare.29974405.

For manuscripts utilizing custom algorithms or software that are central to the research but not yet described in published literature, software must be made available to editors and reviewers. We strongly encourage code deposition in a community repository (e.g. GitHub). See the Nature Portfolio guidelines for submitting code & software for further information.

Data

Policy information about availability of data

All manuscripts must include a data availability statement. This statement should provide the following information, where applicable:

- Accession codes, unique identifiers, or web links for publicly available datasets
- A description of any restrictions on data availability
- For clinical datasets or third party data, please ensure that the statement adheres to our policy

Biodiversity time-series data were obtained from the BioTIME 2.0 database (ref. 38), which is publicly available via Zenodo at https://doi.org/10.5281/

zenodo.10932823 or the BioTIME 2.0 website (http://biotime.st-andrews.ac.uk/). Terrestrial temperature records (CRU TS v4) are available from the Climatic Research Unit, University of East Anglia (https://crudata.uea.ac.uk/cru/data/hrg/; ref. 40). Marine sea surface temperature records (HadISST) are available from the Met Office Hadley Centre (https://www.metoffice.gov.uk/hadobs/hadisst/; ref. 39). The non-climatic anthropogenic driver index was obtained from the Supplementary Information of ref. 41 (https://doi.org/10.1002/pan3.10071).
The processed datasets and source data underlying all figures in this study are available at figshare https://doi.org/10.6084/m9.figshare.29974405.

Research involving human participants, their data, or biological material

Policy information about studies with human participants or human data. See also policy information about sex, gender (identity/presentation), and sexual orientation and race, ethnicity and racism.

Reporting on sex and gender	NA
Reporting on race, ethnicity, or other socially relevant groupings	NA
Population characteristics	NA
Recruitment	NA
Ethics oversight	NA

Note that full information on the approval of the study protocol must also be provided in the manuscript.

Field-specific reporting

Please select the one below that is the best fit for your research. If you are not sure, read the appropriate sections before making your selection.

☐ Life sciences ☐ Behavioural & social sciences ☒ Ecological, evolutionary & environmental sciences

For a reference copy of the document with all sections, see nature.com/documents/nr-reporting-summary-flat.pdf

Ecological, evolutionary & environmental sciences study design

All studies must disclose on these points even when the disclosure is negative.

Study description	We synthesized temporal population trends from 4,748 sites within 129 studies spanning terrestrial, freshwater, and marine ecosystems to evaluate whether initially common or rare species experienced greater changes through time. The design is observational and hierarchically nested: species–time series nested within assemblages, assemblages within study IDs, and study IDs within taxonomic group–realm combinations. Fixed effects include ecological realm, year, and their interaction. Random intercepts and slopes are specified at the study, assemblage, taxonomic group, and species levels.Temporal autocorrelation is modelled with an AR(1) structure. Environmental covariates (temperature change rate, non-climatic anthropogenic drivers) are analyzed using OLS regression.
Research sample	Our dataset comprised 4,748 time series from 129 studies sourced from the BioTIME 2.0 database: 3,457 marine, 1,225 terrestrial, and 66 freshwater assemblages. Time series ranged from 10 to 97 years (mean = 15.9 years), spanning 1910–2023. Taxa included birds, invertebrates, fish, mammals, plants, and multi-taxa assemblages. No sex, age, or individual-level information is available or relevant, as data consist of species-level abundance counts. No experimental manipulations were performed. The sample is intended to represent globally distributed, long-term-monitored assemblages, though coverage is biased toward temperate regions of Europe and North America.
Sampling strategy	We selected time series with ≥10 sampling years, integer abundance data, and species-level resolution. Extreme values (>99.5th percentile) were excluded. Rarefaction standardized sampling effort to the minimum annual sample size per time series. For large-scale studies, distinct assemblages were defined using 96km2 hexagonal grids. No a priori sample size calculation was performed; sample size was determined by the number of time series in BioTIME 2.0 meeting the inclusion criteria. The hierarchical Bayesian framework accommodates unequal sample sizes across nested levels, making it appropriate for the available data structure.
Data collection	This study is a secondary analysis; no original field data collection was conducted by the authors. Primary data were collected by independent research teams associated with each constituent study in BioTIME 2.0, using taxon-appropriate protocols (standardized plot surveys, point counts, trawl surveys, aquatic sampling, etc.). Temperature data were extracted from gridded products: CRU TS v4 (terrestrial, 0.5° resolution) and HadISST (marine, 1° resolution). Anthropogenic driver data were obtained from the published global index (https://doi.org/10.1002/pan3.10071).
Timing and spatial scale	The time series included in the analysis span from 1910 to 2023 (mean duration = 15.9 years, range 10–97 years). Sampling frequency varies by study but is predominantly annual. No systematic gap periods are defined at the database level; each constituent study follows its own sampling schedule. Spatial scale was standardized using96km2 hexagonal grids for large, multi-site studies. Data are distributed globally but are biased toward Europe and North America.
Data exclusions	From BioTIME 2.0, we excluded time series shorter than 10 years, non-integer abundance data, and records without species-leve resolution. We further excluded taxa groups with insufficient sample sizes (reptiles/amphibians, freshwater multi-taxa, and marine plants), and extreme abundance values (primarily marine plankton). The final dataset comprised 4,748 time series from 129 studies.

Reproducibility	All analytical code and processed data available at figshare (https://doi.org/10.6084/m9.figshare.29974405). Raw data accessible via BioTIME 2.0 (http://biotime.st-andrews.ac.uk/). Code includes data filtering, rarefaction, model specifications, and all analyses.
Randomization	This study involves no experimental groups or random allocation, as it is an observational secondary analysis. Species were assigned to abundance intervals (0–20%, 20–40%, 40–60%, 60–80%, 80–100%) based on their log10-transformed initial abundance relative to the assemblage maximum in the first survey year. This assignment is deterministic. Potential confounding from regression-to-the-mean bias was addressed using a truncation-based correction factor calculated separately for each realm.
Blinding	NA (observational study using publicly available data)

Did the study involve field work? ☐ Yes ☒ No

Reporting for specific materials, systems and methods

We require information from authors about some types of materials, experimental systems and methods used in many studies. Here, indicate whether each material, system or method listed is relevant to your study. If you are not sure if a list item applies to your research, read the appropriate section before selecting a response.

Materials & experimental systems

n/a	Involved in the study
☒	☐ Antibodies
☒	☐ Eukaryotic cell lines
☒	☐ Palaeontology and archaeology
☒	☐ Animals and other organisms
☒	☐ Clinical data
☒	☐ Dual use research of concern
☒	☐ Plants

Methods

n/a	Involved in the study
☒	☐ ChIP-seq
☒	☐ Flow cytometry
☒	☐ MRI-based neuroimaging

Plants

Seed stocks	*Report on the source of all seed stocks or other plant material used. If applicable, state the seed stock centre and catalogue number. If plant specimens were collected from the field, describe the collection location, date and sampling procedures.*
Novel plant genotypes	*Describe the methods by which all novel plant genotypes were produced. This includes those generated by transgenic approaches, gene editing, chemical/radiation-based mutagenesis and hybridization. For transgenic lines, describe the transformation method, the number of independent lines analyzed and the generation upon which experiments were performed. For gene-edited lines, describe the editor used, the endogenous sequence targeted for editing, the targeting guide RNA sequence (if applicable) and how the editor was applied.*
Authentication	*Describe any authentication procedures for each seed stock used or novel genotype generated. Describe any experiments used to assess the effect of a mutation and, where applicable, how potential secondary effects (e.g. second site T-DNA insertions, mosiacism, off-target gene editing) were examined.*
