# Supplementary material for: Widespread declines of formerly common species reshape local assemblages over time
Source: Nat Commun. 2026 Sep 12;17:10769. doi: 10.1038/s41467-026-77501-4 (PMC13661514; doi:10.1038/s41467-026-77501-4)
Supplement: Supplementary file 2 [file 41467_2026_77501_MOESM13_ESM.pdf]

nature portfolio

Peer Review File

Widespread declines of formerly common species reshape local assemblages over time

Corresponding Author: Dr Qiang Su

This file contains all reviewer reports in order by version, followed by all author rebuttals in order by version.

Version 0:

Reviewer comments:

Reviewer #1

(Remarks to the Author)
In this manuscript the authors test if initial abundance is related to abundance trends across a large number of time series. This work builds well on emerging evidence that common rather than rare species may be those experiencing the greatest declines. Such a large-scale cross taxa synthesis is vital in furthering this line of inquiry. The manuscript is well written, and the study well conducted.

I would however raise two important issues followed by a number of more minor suggestions.

Firstly, it is great that the authors clearly present effect sizes throughout. It would however be more useful if these could also be presented in a more ecologically meaningful fashion to help the reader understand what this mean in terms of species and populations. For example, what does a slope estimate of 0.0038 mean in the context of total abundance through time in marine systems for the average assemblage or species?

Secondly, although I understand the reasons for much of the data treatment and standardisation processes such as rarefaction, I would want to be assured of the effects (or lack of) on site fidelity. Given, as discussed by the authors local abundance may be very spatially heterogeneous for some species and that some sampling designs are more stable that others in location, I think this is important. For example, marine trawl data may not be repeated in the exact same area. This has different location fidelity to a marked plant plot visited yearly.

Line 60 – delete of

Figure 1 – (a) does not appear to have a common axis with (b) and (c). I think it therefore requires a separate axis label or at least more spacing to make this clear.

Figure 1 – the 80% and 95% confidence intervals are very hard to see in black. As this is a Bayesian model should these be labelled credible intervals (CRI) and not confidence intervals (CI)? They are not the same thing. As INLA is using approximate Bayesian inference I am not completely sure which is being used here. If so, this will need changing throughout the text.

Line 145 – worth clarifying in the main text that 0-20% is a bin

Line 167 to 168 – I don't quite follow this sentence can you please reword.

Line 237 to 239 – I had to read this a few times. You may need to spell this out rather than using "this slope". The other option would be to assign the slope a parameter and refer to this.

Line 250 to 251 – I think this point needs highlighting more explicitly in lines 161 to 173. I was thinking this when reading it but didn't get this point coming through explicitly until now.

Line 342 – I think it should be "where the" rather than "where there"

Line 343 to 345 – Although I agree with this, I think an equally important point is that more work is needed to understand existing data. In many cases we do not know enough about the gradients it spans and how these relate to a globally representative sample.

Line 613 – can the model formula be given rather than the inla code (which is available with other code). This will mean that non-inla users can still follow. Also, what model fit and specification checks were conducted – posterior predictive checks?

(Remarks on code availability)

Reviewer #2

(Remarks to the Author)
This manuscript reports on an analysis of changes in (local) abundance across a relatively large number of taxonomic groups, with good temporal coverage and including marine, terrestrial, and freshwater systems. The major finding reported is a consistent decline across taxonomic group and realm (i.e., marine, terrestrial, freshwater) of common species, whereas rare species show increasing or flat trends.

The scope of the dataset is impressive, and the major conclusions are interesting and topical. The quantitative analysis is also sophisticated. However, I have several major concerns about the analysis, in particular in the way that rare species were chosen and in the interpretation of the Bayesian models. Taken together, I am not convinced that the analysis supports the conclusions, and I would need to see a significant reworking before I am convinced that the results are not an artifact of the methodology.

MAJOR CONCERNS

1. The analyses include time-series with leading zeros. The abundance trends of these 'populations' are therefore expected to be non-negative, i.e., worst case scenario, stable, no trend. At the same time, these 'populations' with leading zeros are categorised as rare species. I worry that this methodological decision artificially inflates the trend estimates for 'rare' species. These species definitionally cannot decline, and therefore will bias the estimation for rare species towards positive slopes.

Extended Data Fig 7 highlights this point – when removing species with leading zeros, rare species population trends are negative (although still less negative than those for common species). Van Klink et al 2023, which the current paper draws on heavily, excluded species-assemblage time-series with leading zeros for the reasons highlighted above. Unless the authors can strongly justify the inclusion of 'colonising' species (or missed detections), I would be in favour of presenting results excluding time-series with leading zeros, due to their more conservative nature. Further, the interpretation of these leading zeros has important ecological consequences. If they are 'true' zeros, such species represent colonising (not rare) species, whereas 'false' zeros or missed detections do suggest rare species. Linking back to Ext Fig 7, when ignoring initial zeros (and assuming these represent colonisation) rare species do decline. The paper could then conclude that colonising species increases are outweighing declines in 'native', rare species.

2. Using the log(y + 1) as the response variable is a potentially problematic choice, as is the inclusion of species with zero initial abundance as "rare" species. These species are not rare at the local scale, they are absent, and they can only increase from there. But your statistical model assumes they are rare by adding one for statistical convenience. On the log scale this is an infinite increase with no meaningful interpretation! Although it is a common practise to add a small number to log-gaussian models in order to account for observed zeros, in a dataset with many zeros this has non-trivial implications for estimation (adding one to an observed zero is much more impactful than adding one to a large number). It is more common in Bayesian models to incorporate a likelihood structure that more closely reflects the statistical process generating the data. In this case, a Poisson or Negative Bionomial likelihood function would seem to be appropriate for abundance data, possibly with a finite mixture to account for many processes that can generate zeros (e.g., true absence, low abundance leading to nondetection, etc).

3. The paper's current approach to assigning species to 'initial abundance bins' per site is based on the maximum (log) abundance value across the entire time-series. I believe this will lead to an artificial reduction in the trends of the most common 'assigned' species – if their abundance is >80% of the time-series max in the first year of the time-series, they are likely to subsequently decline. Again, Extended Data Fig 4 suggests this occurs, left truncation (of even 1 year) reduces Marine population trends to ~0 for all abundance bins, with the most 'common' species displaying positive trends, on average after left truncation. I would favour a more conservative approach for assigning species to abundance bins. Specifically, either i) using (log) abundance values for the initial year, and creating bins based on the maximum of these values (Van Klink et al, 2023), ii) using average (log) abundance values for the first 3 to 5 years (per species), and creating bins based on the maximum of these values, or iii) most conservatively, using average (log) abundance values for the entire time-series (per species), and creating bins based on the maximum of these values.

A further concern, although hard to remedy, is that allocations of abundance classes are based on per assemblage relative abundances. It's possible that in two different assemblages, a species with similar (or even the same) absolute abundances could be categorised as rare in one but common in the other – depending on the other species present. Alternatively, if an

assemblage consists of only objectively (low absolute abundance) rare species, all could be categorised as common. Is there an alternative approach, based on regional, or study-level measure of rarity or similar, that could be used to test the robustness of these abundance-class assignments?

4. I am concerned about the precision and accuracy of the tails of the posterior distributions, and in general about the influence of extreme values on estimation and inference. INLA estimates an approximate posterior distribution, and such approximations will necessarily be more accurate near the centre of the distribution. Moreover, using only 1000 samples from the posterior distribution means that only 50 samples are available for estimating the tails of a 95% credible interval, and only 25 for determining whether a given interval overlaps zero, which is critical to the interpretation of a "strong" effect in the ms. Given the importance of tail estimation to the conclusions, at a minimum, the authors should attempt to quantify tail uncertainty, and to refine the methods to improve tail sample size and precision.

Moreover, the figures in the ms consistently exclude extreme values. While this can improve visualisation by reducing the scale on the axes, it also makes it impossible to evaluate whether such extremes might influence parameter estimates. I find myself wondering what values are being excluded from the figures, and in what direction they influence the slopes. I think it is essential to include figures with all data points, if not in the main ms then at least in the SI.

MINOR/SPECIFIC COMMENTS

Line 2: The title emphasises declines in common species, yet Fig 1c indicates that rare species contribute most to the assemblage level changes in total abundance (for Marine populations at least). I think maintaining consistency in your narrative is important for the reader to follow your arguments/conclusions.

L35: Although the data are impressive, I think the geographic biases prevent statements about 'global erosion of formerly common species'. 'Widespread declines in common species' or similar may be more appropriate.
I also think the term 'erosion' is very strong, given the small-ish number of 'common' species (relative to rare) in this analysis (e.g., 295 common v 5625 rare for Terrestrial).

L52: Do you mean abundances of individual species, or total assemblage abundances?

L60: 'of across' – I think a word is missing. Abundance?

Line 62: Temperature is not a resource. Heat or energy perhaps, but not temperature.

Fig 1: I found the lack of x-axis labels in row b confusing -- rows b and c share an x-axis scale, but not row a, and this was not obvious. I suggest repeating the axis labels in row b for clarity.

L64-66: The final sentence of this paragraph largely repeats lines 50-54.

L69-70: Which populations are emphasised in the mentioned indicators/data? An indication of some of the biases, currently not stated, would be useful for the reader to know what specifically you are commenting on. Geographic bias? Taxonomic focus on vertebrates?

L97: What do you mean by 'species-level'? The model estimates overall trends by realm, and for each random effect, but there isn't a 'species-level' random effect, only a 'species-time series' one. This is finer that species-level, in my opinion.

L102-103: The inclusion of taxonomic group as a random effect is already stated on L98.

L105: I would consider climate/temperature change a human impact. And the reference used for human impacts incorporates climate change (although I know you remove it). Clearer distinction/naming between the two data types would be useful, e.g. 'Climate change' and separately, 'non-climatic drivers'.

L111: 0.0038 is a small number, even if the CIs do not overlap 0. Could you contextualise this number? e.g. % population change, expected loss/gain in a population of 100 individuals over a given time-period (one year, or maybe a decade). Van Klink et al. 2023 present these values a both 'trends', and % population change per year, which I think helps the reader better interpret the results.

L131: There also appear to be some negative peaks in the distribution for marine total abundance trends. Given this work focuses on the nuances of biodiversity change, it would be nice to acknowledge these apparent declines in assemblage-level abundance – and potentially discuss them and their possible causes.

L135: I think '90%' is missing in the list of CI widths.

L136-141: 'Common species contribute most…' – I am a bit lost as to where this statement should go/what it relates to. Total abundance? In Fig 1c, and the below sentence, you state that "Rare species contribute more than expected on a per-abundance basis". Although the largest (absolute) contribution in freshwater are the two most common abundance classes.

In this context, what would the expected contribution be? 20%? But see a later comment about accounting for number of species present per abundance class.

L154: What are the scales of abundance values involved in defining rare to common species? E.g. average initial abundance per 'bin'. Do these distributions shift substantially across Realms? Across time-series (sites), studies?

L158: 'due' – I am not sure this word is needed.

L166-168: Why would species near the centre of their range be more exposed to environmental pressure?

L183: I would interpret Ext Data Fig 5 as suggesting weak support (80% CI) for changes to Hill numbers and Evenness in Marine and Terrestrial realms.

L215-216: I'm not sure your results do suggest that 'most assemblages were previously structured around some formerly common species that are no longer so'. They suggest common species have declined, whilst rarer species have increased, but the magnitude of these changes appears small. It is possible that formerly abundant species are still the most abundant within that assemblage. I think context relating to the initial abundance values, and % change through time would help the interpretation of this. As a further test you could categorise species as rare/common based on their end/last 5 abundance values. If these categories differ from their initial abundance assignments, you would have more support for your conclusion of substantial changes in rank abundance. However, if they remain largely unchanged, your statement would need to be adjusted accordingly.

L228-229: The declines in marine species presented here (Fig 2b) are substantially greater than those in Fig 1b? Why? How? Aren't these outputs from the same model, but re-organised to reflect taxonomic variation? Have the estimates been corrected for regression to the mean?

L236: Should this read 'steeper negative slopes'? Steep positive slopes would suggest initially common species doing proportionately better.

L245-247 and L261-262: The positive relationship in Fig 3 also suggests that communities experiencing net gains in abundance exhibit steeper (positive) trend-abundance relationships (although less substantial).

L264-265: Would the reasoning not be the other way around? i.e., trends in total assemblage are contingent on species-level changes.

L282-283: Fig 4 suggests that warming and human impact are associated with 'more positive' trends in common species, not necessarily actual positive trends. Fig 2 suggests most/all common species declined in the marine realm, an association of warming with actual positive trends seems unlikely.

L316: Your results indicate a strong signal of change, however the magnitude of change appears small, rather than strong. Again, clarity regarding the interpretation of population trend estimate magnitudes would be useful.

L331-334: Equivalently, regionally widespread species, can be locally rare – e.g. large carnivores. I wonder if a comparison between range-based commonness and your abundance-based allocation could be done? Would they align?

L556: How many time-series contain constant species abundance? Is this a data error?

L569: Do 'rarefied samples' refer to the median of rarefaction samples? Or individual samples?

L587: Does '20 years of sampling data' refer to 20 sampling time-points, or a total time-series length of 20 years?

L590: Clearer description of your correction factor approach would be helpful. Is there a correction factor per realm:taxon combination?
Does using long time-series under-estimate potential correction factors? Long time-series are typically more stable than shorter ones.

L610: Do you test model residuals for spatial/phylogenetic correlation?

L627: Is a gaussian error distribution suitable for Evenness (bounded 0-1)? Did you check model residuals?

L636-641: Are abundance change slopes (s) in log-10 space? Do these need to be transformed before estimating N. Or is N in log-10 space?
Your code suggests the use of values on the log10 scale, but this is not clear in the methods.
How does use of log-space affect weighting of rare vs common species? I suspect it (relatively) upweights the contributions of rare species. Are you interested in additive abundance change, or relative/multiplicative abundance change? I thought the former, given your description of N etc, but the use of log10 space would suggest the latter.

Your N calculation accounts for the initial abundance of a species, which you then sum within each abundance interval to generate Ci. Unless I have misunderstood, your values of Ci are therefore affected by the total number of species in each

interval – and this is highly uneven, there are many more rare species than common ones and so their expected contribution would be higher (e.g., 3162 rare Marine species v 207 common ones). I think you either need to define an expected contribution per abundance class, accounting for initial abundance and number of species per class, or estimate the 'average' contribution of each abundance bin (i.e., controlling for number of species in the calculation of Ci). I think both would be more informative than the current measure.

Sites also vary in their time-series lengths which affects their N value. The relative distribution of abundance intervals across time-series lengths can therefore also affect N and Ci values. For example, a particularly long time-series may, by chance have many, or few rare species. I would assume these influences average out, but it is possible that they don't. Maybe using a standard time-series duration would better control for this?

L671: I can't see baseline temperature in Extended Fig 6.

L694-695: 'applied the same methodology as above' – Two alternative methodologies for assigning abundance intervals (five evenly space bins v quartiles) have been described. Which one is this referring to?

L742/Ext Fig 1: Data is mostly from 'western'/northern countries – highly representative of modified ecosystems. How is this likely to affect your conclusions? Could the positive relationship between abundance trends and climate change/human impacts (Ext Dat Fig 6, Marine) be due to recent population recoveries in these regions (post historical declines) co-occurring with ongoing global warming/human modification?

L756: There don't appear to be any neutral trends presented in this figure, no credible intervals overlap 0.

L760/Ext Fig 3: I think the approach here for assigning abundance classes is more robust than the one presented in the main text.

L782: You highlight a more conservative correction approach, but do not use it. Why not? In many realm:taxon combinations, greater regression to the mean is seen under 3-year truncation than 1-year. Does this not suggest that using correction factors based on 1-year truncation is insufficient to address the issues of regression to the mean?

L808/Ext Fig 7: Excluding species with 0s in the start year completely removes the increases of rare species presented in the main results. I think this undermines the robustness of your main conclusions – at least with regards to rare species.

(Remarks on code availability)

Reviewer #3

(Remarks to the Author)
I co-reviewed this manuscript with one of the reviewers who provided the listed reports. This is part of the Nature Communications initiative to facilitate training in peer review and to provide appropriate recognition for Early Career Researchers who co-review manuscripts.

(Remarks on code availability)

Version 1:

Reviewer comments:

Reviewer #1

(Remarks to the Author)
The manuscript is much improved, and the authors have conducted very comprehensive sensitivity analyses to address the reviewer comments.
I just have a few remaining queries below.
Line 38 – maybe change to "Rapid biodiversity change" as biodiversity change in some form is a constant.
Line 344 – I can only see "non-climatic anthropogenic drivers" defined in the method section. It needs to be explained on first use. Here it makes it sound like they are basically land-use change but line 774 lists a far longer array. Many of these are hard to measure and the data used is derived from relatively broad scales in some cases. This may be another reason for the discrepancies reported. This is already alluded to by "unmeasured pressures" in line 343. It may not be that they are unmeasured as such, but the data quality available is limited.
Line 382 – BioTIME 2.0
Line 627 to 629 – Sorry, I am still not completely following the rarefaction. If the minimum annual sampling effort was three plots for example does this mean that for a given iteration the same three plots are used in each year? But then I do not understand how multiple iterations can be performed as at least 1 year only has three plots (which must be used for each year). If, however, three random plots are chosen each year then this seems counter productive as it is adding to spatial variability. 96km2 is huge for many of the taxa under consideration. Is this only applied in situations when site fidelity is already low?

Line 695 – I think the main caveat of not including spatial and phylogenetic structures into the analysis is that the results are only representative of the sample not the statistical population (global local assemblages across taxa) that you are aiming to estimate for. This now appears to be clearly mentioned in the text with the discussion of data representativeness, and I can see the computational and technical challenges of including space and phylogeny.
Line 855 to 856 _ I am not sure that point size is really visible here given the overlap of points in most regions. Showing this in another way or another panel may be better.
Line 914 – could the original (all time series boxes) be added in a different colour to make it easy to quickly see the difference.

(Remarks on code availability)

Reviewer #2

(Remarks to the Author)
This review was written by the co-reviewer, the other co-reviewer was unable to read the manuscript.

I thank the authors for taking the time to respond to our concerns and comments. I believe the authors adequately address most of our concerns (e.g. allocation of abundance bins and statistical models), and adequately present the alternative considerations of initial zeros. However, there are still a few points that I would like to see addressed.

On the title
These analyses are of local assemblages, I would use that in the title rather than global. As the authors state, their analysis is on 'within-assemblage dominance hierarchies and community reorganisation', this feels like a local (assemblage-level), rather than global process. I would also be cautious that this process does not seem to be occurring in the freshwater realm, with stable trends across all abundance bins in Fig 1.
A title more accurately representing the results would be desirable.
e.g. "Widespread declines of formerly common species reshape local assemblages over time".

I think a heatmap/Sankey plot or similar presenting abundance bin transitions for each realm would be a nice addition to one of the main figures, this is a key result relating to 'reorganisation' of local assemblages. Currently Table S7 presents an overall transition matrix, however I believe a realm-specific version is needed to support Fig 1. You also do not describe your methods for this table. I realise it is a fairly straightforward approach, but I think including the methods is needed. In particular, your description of how you calculated abundance bins (based on your response, average relative abundance in first 5 v last 5 years) differs from the approach used to generate abundance bins in your main results (log(abundance + 1)) – ignoring the 5-year v 1-year component. It's hard to know how much this would influence abundance bin allocation, but I think being consistent in the use of linear v log scales would be preferable.

I am not sure the authors responses regarding not checking residuals for spatial and phylogenetic correlation stand up to scrutiny. Whilst I agree that such residual correlation structure may not affect the direction of their trend estimates, it could be affecting the uncertainty. As noted in Johnson et al., 2024, and in this paper's introduction (L 50-52) accounting for spatial and phylogenetic structure in population abundance datasets, including BioTIME, can substantially increase uncertainty in trend estimates. Without an assessment of model residuals, readers cannot usefully conclude how valid the presented models are.
Furthermore, the authors claim in their response that within the main document (on L690) they acknowledge "that unmodelled phylogenetic dependence—where present—could lead to underestimated uncertainty and should be considered when interpreting our inference". However, such an acknowledgment is missing from the main text.

Further line-specific comments

L 28: Initially common freshwater species do not demonstrate declining trends.

L 30: I think a "," after included would be needed to make the sentence clearer.

L 140-141 (Fig 1c): Based on your updated methodology for calculating Pi (Lines 720-741), I don't think this is estimating contribution to 'total abundance change'. Your estimates and calculations remain on the log-scale, thus your measures, as stated in the methods correspond to the log of relative abundance change. Given that you then standardise this by time-series length, and average over time-series per bin, I don't fully see the value of this figure – doesn't it just largely reproduce your estimated average trends in Fig 1b but on a different scale (and including time-series-species random effects)?

L 238-9: Initially common freshwater species do not demonstrate declining trends. Rephrase to reflect "marine and terrestrial taxa" or similar.

L 243-248: This is not linked to the taxa-specific results and seems a bit out of place here. It relates to overall results.

L 720: I'm not sure your calculations for deltaN, Ci, Pi, relate to total abundance change. See previous comment relating to L140/Fig 1c.

Extended data fig 6: Without censoring, large uncertainty in trends of rare species is consistently present. This uncertainty substantially declines under 1-, or 3-year censoring. Why does this happen? I know these are longer time-series (20+ years) than in the main results, but the substantial change in model uncertainty seems surprising, especially when similar patterns are not seen to the same extent for the other abundance intervals.

Finally, in their response, the authors claim that under-correction of regression to the mean is conservative. Unless I have misunderstood the methodology, I believe the opposite is true in relation to their results. A smaller magnitude of adjustment would, I expect, lead to the retention of trend estimates that are further from zero than they should be if properly accounting for regression to the mean, not the other way around.

(Remarks on code availability)

Reviewer #3

(Remarks to the Author)
I co-reviewed this manuscript with one of the reviewers who provided the listed reports. This is part of the Nature Communications initiative to facilitate training in peer review and to provide appropriate recognition for Early Career Researchers who co-review manuscripts.

(Remarks on code availability)

Version 2:

Reviewer comments:

Reviewer #1

(Remarks to the Author)
I agree that the revisions and response from the authors have now adequately addressed the issues I previously raised. I therefore now have no further suggestions or comments.

(Remarks on code availability)

Reviewer #2

(Remarks to the Author)
I would like to thank the authors for their attention to detail in revising the manuscript. I am satisfied that my concerns have been addressed and have no further feedback at this time.

(Remarks on code availability)

Reviewer #3

(Remarks to the Author)
I co-reviewed this manuscript with one of the reviewers who provided the listed reports. This is part of the Nature Communications initiative to facilitate training in peer review and to provide appropriate recognition for Early Career Researchers who co-review manuscripts.

(Remarks on code availability)

Open Access This Peer Review File is licensed under a Creative Commons Attribution 4.0 International License, which permits use, sharing, adaptation, distribution and reproduction in any medium or format, as long as you give appropriate credit to the original author(s) and the source, provide a link to the Creative Commons license, and indicate if changes were made.
In cases where reviewers are anonymous, credit should be given to 'Anonymous Referee' and the source.
The images or other third party material in this Peer Review File are included in the article's Creative Commons license, unless indicated otherwise in a credit line to the material. If material is not included in the article's Creative Commons license and your intended use is not permitted by statutory regulation or exceeds the permitted use, you will need to obtain permission directly from the copyright holder.
To view a copy of this license, visit https://creativecommons.org/licenses/by/4.0/

Manuscript Title: The decline of formerly common species reshapes global biodiversity over time

Reviewer Comments & Author Response

Reviewer Reports on the Initial Version:

Referees' comments:

Reviewer #1 (Remarks to the Author):

In this manuscript the authors test if initial abundance is related to abundance trends across a large number of time series. This work builds well on emerging evidence that common rather than rare species may be those experiencing the greatest declines. Such a large-scale cross taxa synthesis is vital in furthering this line of inquiry. The manuscript is well written, and the study well conducted.

I would however raise two important issues followed by a number of more minor suggestions.

Firstly, it is great that the authors clearly present effect sizes throughout. It would however be more useful if these could also be presented in a more ecologically meaningful fashion to help the reader understand what this mean in terms of species and populations. For example, what does a slope estimate of 0.0038 mean in the context of total abundance through time in marine systems for the average assemblage or species?

Secondly, although I understand the reasons for much of the data treatment and standardisation processes such as rarefaction, I would want to be assured of the effects (or lack of) on site fidelity. Given, as discussed by the authors local abundance may be very spatially heterogeneous for some species and that some sampling designs are more stable that others in location, I think this is important. For example, marine trawl data may not be repeated in the exact same area. This has different location fidelity to a marked plant plot visited yearly.

Line 60 – delete of

Figure 1 – (a) does not appear to have a common axis with (b) and (c). I think it therefore requires a separate axis label or at least more spacing to make this clear.

Figure 1 – the 80% and 95% confidence intervals are very hard to see in black. As this is a Bayesian model should these be labelled credible intervals (CRI) and not confidence intervals (CI)? They are not the same thing. As INLA is using approximate

Bayesian inference I am not completely sure which is being used here. If so, this will need changing throughout the text.

Line 145 – worth clarifying in the main text that 0-20% is a bin

Line 167 to 168 – I don't quite follow this sentence can you please reword.

Line 237 to 239 – I had to read this a few times. You may need to spell this out rather than using "this slope". The other option would be to assign the slope a parameter and refer to this.

Line 250 to 251 – I think this point needs highlighting more explicitly in lines 161 to 173. I was thinking this when reading it but didn't get this point coming through explicitly until now.

Line 342 – I think it should be "where the" rather than "where there"

Line 343 to 345 – Although I agree with this, I think an equally important point is that more work is needed to understand existing data. In many cases we do not know enough about the gradients it spans and how these relate to a globally representative sample.

Line 613 – can the model formula be given rather than the inla code (which is available with other code). This will mean that non-inla users can still follow. Also, what model fit and specification checks were conducted – posterior predictive checks?

Reviewer #2 (Remarks to the Author):

This manuscript reports on an analysis of changes in (local) abundance across a relatively large number of taxonomic groups, with good temporal coverage and including marine, terrestrial, and freshwater systems. The major finding reported is a consistent decline across taxonomic group and realm (i.e., marine, terrestrial, freshwater) of common species, whereas rare species show increasing or flat trends.

The scope of the dataset is impressive, and the major conclusions are interesting and topical. The quantitative analysis is also sophisticated. However, I have several major concerns about the analysis, in particular in the way that rare species were chosen and in the interpretation of the Bayesian models. Taken together, I am not convinced that the analysis supports the conclusions, and I would need to see a significant reworking before I am convinced that the results are not an artifact of the methodology.

MAJOR CONCERNS

1. The analyses include time-series with leading zeros. The abundance trends of these ‘populations’ are therefore expected to be non-negative, i.e., worst case scenario, stable, no trend. At the same time, these ‘populations’ with leading zeros are categorised as rare species. I worry that this methodological decision artificially inflates the trend estimates for ‘rare’ species. These species definitionally cannot decline, and therefore will bias the estimation for rare species towards positive slopes.

Extended Data Fig 7 highlights this point – when removing species with leading zeros, rare species population trends are negative (although still less negative than those for common species). Van Klink et al 2023, which the current paper draws on heavily, excluded species-assemblage time-series with leading zeros for the reasons highlighted above. Unless the authors can strongly justify the inclusion of ‘colonising’ species (or missed detections), I would be in favour of presenting results excluding time-series with leading zeros, due to their more conservative nature. Further, the interpretation of these leading zeros has important ecological consequences. If they are ‘true’ zeros, such species represent colonising (not rare) species, whereas ‘false’ zeros or missed detections do suggest rare species. Linking back to Ext Fig 7, when ignoring initial zeros (and assuming these represent colonisation) rare species do decline. The paper could then conclude that colonising species increases are outweighing declines in ‘native’, rare species.

2. Using the log(y + 1) as the response variable is a potentially problematic choice, as is the inclusion of species with zero initial abundance as "rare" species. These species are not rare at the local scale, they are absent, and they can only increase from there. But your statistical model assumes they are rare by adding one for statistical convenience. On the log scale this is an infinite increase with no meaningful interpretation! Although it is a common practise to add a small number to log-gaussian models in order to account for observed zeros, in a dataset with many zeros this has non-trivial implications for estimation (adding one to an observed zero is much more impactful than adding one to a large number). It is more common in Bayesian models to incorporate a likelihood structure that more closely reflects the statistical process generating the data. In this case, a Poisson or Negative Bionomial likelihood function would seem to be appropriate for abundance data, possibly with a finite mixture to account for many processes that can generate zeros (e.g., true absence, low abundance leading to nondetection, etc).

3. The paper's current approach to assigning species to 'initial abundance bins' per site is based on the maximum (log) abundance value across the entire time-series. I believe this will lead to an artificial reduction in the trends of the most common 'assigned' species – if their abundance is >80% of the time-series max in the first year of the time-series, they are likely to subsequently decline. Again, Extended Data Fig 4 suggests this occurs, left truncation (of even 1 year) reduces Marine population trends to ~0 for all abundance bins, with the most 'common' species displaying positive trends, on average after left truncation. I would favour a more conservative approach for assigning species to abundance bins. Specifically, either i) using (log) abundance values for the initial year, and creating bins based on the maximum of these values (Van Klink et al, 2023), ii) using average (log) abundance values for the first 3 to 5 years (per species), and creating bins based on the maximum of these values, or iii) most conservatively, using average (log) abundance values for the entire time-series (per species), and creating bins based on the maximum of these values.

A further concern, although hard to remedy, is that allocations of abundance classes are based on per assemblage relative abundances. It's possible that in two different assemblages, a species with similar (or even the same) absolute abundances could be categorised as rare in one but common in the other – depending on the other species present. Alternatively, if an assemblage consists of only objectively (low absolute abundance) rare species, all could be categorised as common. Is there an alternative approach, based on regional, or study-level measure of rarity or similar, that could be used to test the robustness of these abundance-class assignments?

4. I am concerned about the precision and accuracy of the tails of the posterior distributions, and in general about the influence of extreme values on estimation and inference. INLA estimates an approximate posterior distribution, and such approximations will necessarily be more accurate near the centre of the distribution. Moreover, using only 1000 samples from the posterior distribution means that only 50 samples are available for estimating the tails of a 95% credible interval, and only 25 for determining whether a given interval overlaps zero, which is critical to the interpretation of a "strong" effect in the ms. Given the importance of tail estimation to the conclusions, at a minimum, the authors should attempt to quantify tail uncertainty, and to refine the methods to improve tail sample size and precision.

Moreover, the figures in the ms consistently exclude extreme values. While this can improve visualisation by reducing the scale on the axes, it also makes it impossible to evaluate whether such extremes might influence parameter estimates. I find myself wondering what values are being excluded from the figures, and in what direction

they influence the slopes. I think it is essential to include figures with all data points, if not in the main ms then at least in the SI.

Line 2: The title emphasises declines in common species, yet Fig 1c indicates that rare species contribute most to the assemblage level changes in total abundance (for Marine populations at least). I think maintaining consistency in your narrative is important for the reader to follow your arguments/conclusions.

L35: Although the data are impressive, I think the geographic biases prevent statements about ‘global erosion of formerly common species’. ‘Widespread declines in common species’ or similar may be more appropriate.

I also think the term ‘erosion’ is very strong, given the small-ish number of ‘common’ species (relative to rare) in this analysis (e.g., 295 common v 5625 rare for Terrestrial).

L52: Do you mean abundances of individual species, or total assemblage abundances?

L60: ‘of across’ – I think a word is missing. Abundance?

Line 62: Temperature is not a resource. Heat or energy perhaps, but not temperature.

Fig 1: I found the lack of x-axis labels in row b confusing -- rows b and c share an x-axis scale, but not row a, and this was not obvious. I suggest repeating the axis labels in row b for clarity.

L64-66: The final sentence of this paragraph largely repeats lines 50-54.

L69-70: Which populations are emphasised in the mentioned indicators/data? An indication of some of the biases, currently not stated, would be useful for the reader to know what specifically you are commenting on. Geographic bias? Taxonomic focus on vertebrates?

L97: What do you mean by ‘species-level’? The model estimates overall trends by realm, and for each random effect, but there isn’t a ‘species-level’ random effect, only a ‘species-time series’ one. This is finer that species-level, in my opinion.

L102-103: The inclusion of taxonomic group as a random effect is already stated on L98.

L105: I would consider climate/temperature change a human impact. And the reference used for human impacts incorporates climate change (although I know you remove it). Clearer distinction/naming between the two data types would be useful,

e.g. ‘Climate change’ and separately, ‘non-climatic drivers’.

L111: 0.0038 is a small number, even if the CIs do not overlap 0. Could you contextualise this number? e.g. % population change, expected loss/gain in a population of 100 individuals over a given time-period (one year, or maybe a decade). Van Klink et al. 2023 present these values a both ‘trends’, and % population change per year, which I think helps the reader better interpret the results.

L131: There also appear to be some negative peaks in the distribution for marine total abundance trends. Given this work focuses on the nuances of biodiversity change, it would be nice to acknowledge these apparent declines in assemblage-level abundance – and potentially discuss them and their possible causes.

L135: I think ‘90%’ is missing in the list of CI widths.

L136-141: ‘Common species contribute most…’ – I am a bit lost as to where this statement should go/what it relates to. Total abundance? In Fig 1c, and the below sentence, you state that “Rare species contribute more than expected on a per-abundance basis”. Although the largest (absolute) contribution in freshwater are the two most common abundance classes. In this context, what would the expected contribution be? 20%? But see a later comment about accounting for number of species present per abundance class.

L154: What are the scales of abundance values involved in defining rare to common species? E.g. average initial abundance per ‘bin’. Do these distributions shift substantially across Realms? Across time-series (sites), studies?

L158: ‘due’ – I am not sure this word is needed.

L166-168: Why would species near the centre of their range be more exposed to environmental pressure?

L183: I would interpret Ext Data Fig 5 as suggesting weak support (80% CI) for changes to Hill numbers and Evenness in Marine and Terrestrial realms.

L215-216: I’m not sure your results do suggest that ‘most assemblages were previously structured around some formerly common species that are no longer so’. They suggest common species have declined, whilst rarer species have increased, but the magnitude of these changes appears small. It is possible that formerly abundant species are still the most abundant within that assemblage. I think context relating to the initial abundance values, and % change through time would help the interpretation of this. As a further test you could categorise species as rare/common based on their end/last 5 abundance values. If these categories differ from their initial

abundance assignments, you would have more support for your conclusion of substantial changes in rank abundance. However, if they remain largely unchanged, your statement would need to be adjusted accordingly.

L228-229: The declines in marine species presented here (Fig 2b) are substantially greater than those in Fig 1b? Why? How? Aren't these outputs from the same model, but re-organised to reflect taxonomic variation? Have the estimates been corrected for regression to the mean?

L236: Should this read 'steeper negative slopes'? Steep positive slopes would suggest initially common species doing proportionately better.

L245-247 and L261-262: The positive relationship in Fig 3 also suggests that communities experiencing net gains in abundance exhibit steeper (positive) trend-abundance relationships (although less substantial).

L264-265: Would the reasoning not be the other way around? i.e., trends in total assemblage are contingent on species-level changes.

L282-283: Fig 4 suggests that warming and human impact are associated with 'more positive' trends in common species, not necessarily actual positive trends. Fig 2 suggests most/all common species declined in the marine realm, an association of warming with actual positive trends seems unlikely.

L316: Your results indicate a strong signal of change, however the magnitude of change appears small, rather than strong. Again, clarity regarding the interpretation of population trend estimate magnitudes would be useful.

L331-334: Equivalently, regionally widespread species, can be locally rare – e.g. large carnivores. I wonder if a comparison between range-based commonness and your abundance-based allocation could be done? Would they align?

L556: How many time-series contain constant species abundance? Is this a data error?

L569: Do 'rarefied samples' refer to the median of rarefaction samples? Or individual samples?

L587: Does '20 years of sampling data' refer to 20 sampling time-points, or a total time-series length of 20 years?

L590: Clearer description of your correction factor approach would be helpful. Is there a correction factor per realm:taxon combination?

Does using long time-series under-estimate potential correction factors? Long time-

series are typically more stable than shorter ones.

L610: Do you test model residuals for spatial/phylogenetic correlation?

L627: Is a gaussian error distribution suitable for Evenness (bounded 0-1)? Did you check model residuals?

L636-641: Are abundance change slopes (s) in log-10 space? Do these need to be transformed before estimating N. Or is N in log-10 space?

Your code suggests the use of values on the log10 scale, but this is not clear in the methods.

How does use of log-space affect weighting of rare vs common species? I suspect it (relatively) upweights the contributions of rare species. Are you interested in additive abundance change, or relative/multiplicative abundance change? I thought the former, given your description of N etc, but the use of log10 space would suggest the latter.

Your N calculation accounts for the initial abundance of a species, which you then sum within each abundance interval to generate Ci. Unless I have misunderstood, your values of Ci are therefore affected by the total number of species in each interval – and this is highly uneven, there are many more rare species than common ones and so their expected contribution would be higher (e.g., 3162 rare Marine species v 207 common ones). I think you either need to define an expected contribution per abundance class, accounting for initial abundance and number of species per class, or estimate the 'average' contribution of each abundance bin (i.e., controlling for number of species in the calculation of Ci). I think both would be more informative than the current measure.

Sites also vary in their time-series lengths which affects their N value. The relative distribution of abundance intervals across time-series lengths can therefore also affect N and Ci values. For example, a particularly long time-series may, by chance have many, or few rare species. I would assume these influences average out, but it is possible that they don't. Maybe using a standard time-series duration would better control for this?

L671: I can't see baseline temperature in Extended Fig 6.

L694-695: 'applied the same methodology as above' – Two alternative methodologies for assigning abundance intervals (five evenly space bins v quartiles) have been described. Which one is this referring to?

L742/Ext Fig 1: Data is mostly from 'western'/northern countries – highly

representative of modified ecosystems. How is this likely to affect your conclusions? Could the positive relationship between abundance trends and climate change/human impacts (Ext Dat Fig 6, Marine) be due to recent population recoveries in these regions (post historical declines) co-occurring with ongoing global warming/human modification?

L756: There don't appear to be any neutral trends presented in this figure, no credible intervals overlap 0.

L760/Ext Fig 3: I think the approach here for assigning abundance classes is more robust than the one presented in the main text.

L782: You highlight a more conservative correction approach, but do not use it. Why not? In many realm: taxon combinations, greater regression to the mean is seen under 3-year truncation than 1-year. Does this not suggest that using correction factors based on 1-year truncation is insufficient to address the issues of regression to the mean?

L808/Ext Fig 7: Excluding species with 0s in the start year completely removes the increases of rare species presented in the main results. I think this undermines the robustness of your main conclusions – at least with regards to rare species.

Reviewer #3 (Remarks to the Author):

I co-reviewed this manuscript with one of the reviewers who provided the listed reports. This is part of the Nature Communications initiative to facilitate training in peer review and to provide appropriate recognition for Early Career Researchers who co-review manuscripts.

Author Response to Initial Comments:

Reviewer #1 (Remarks to the Author)

In this manuscript the authors test if initial abundance is related to abundance trends across a large number of time series. This work builds well on emerging evidence that common rather than rare species may be those experiencing the greatest declines. Such a large-scale cross taxa synthesis is vital in furthering this line of inquiry. The manuscript is well written, and the study well conducted.

RESPONSE: Thanks for your positive feedback.

I would however raise two important issues followed by a number of more minor suggestions.

Firstly, it is great that the authors clearly present effect sizes throughout. It would however be more useful if these could also be presented in a more ecologically meaningful fashion to help the reader understand what this mean in terms of species and populations. For example, what does a slope estimate of 0.0038 mean in the context of total abundance through time in marine systems for the average assemblage or species?

RESPONSE: We now report both the raw slope estimates and their corresponding percentage changes for Figures 1&2 throughout the manuscript. With the additional data-screening rules applied (see Reviewer 2's second Major Concern for details), the marine total-abundance slope is now 0.0032, corresponding to an annual increase of approximately 0.73% in expected total abundance. This approach allows readers to better understand what these effect sizes mean for actual species populations and assemblages.

Secondly, although I understand the reasons for much of the data treatment and standardisation processes such as rarefaction, I would want to be assured of the effects (or lack of) on site fidelity. Given, as discussed by the authors local abundance may be very spatially heterogeneous for some species and that some sampling designs are more stable that others in location, I think this is important. For example, marine trawl data may not be repeated in the exact same area. This has different location fidelity to a marked plant plot visited yearly.

RESPONSE: It is true that spatial consistency of sampling locations through time varies across studies—ranging from permanently marked plots (high fidelity) to surveys like marine trawls where exact positions may shift between years. Such variation could indeed introduce noise due to spatial heterogeneity rather than genuine temporal change.

In our original workflow, we implemented two complementary standardization procedures that address related—but not identical—sources of bias:

1. Spatial domain standardization (gridding): samples from large-extent studies were assigned to 96 km^2 hexagonal grid cells based on coordinates, ensuring temporal comparisons are made within consistent spatial domains and reducing bias from changes in study extent through time.
2. Temporal effort standardization (sample-based rarefaction): within each time step, we subsampled to the minimum annual sample size (199 iterations; median retained), controlling for inter-annual variation in sampling intensity and coverage.

However, we acknowledge that gridding and rarefaction do not, by themselves, recreate strict site fidelity equivalent to permanently marked sampling locations. To address the reviewer's specific concern directly, we classified all 136 studies using the metadata into three site-fidelity levels based on explicit evidence in the Methods fields:

High fidelity: explicit permanent/fixed/marked plots, transects, stations, routes, or grids repeatedly sampled at the same locations.

Median fidelity: repeated sampling within a consistent spatial domain (e.g., fixed area/defined transect or lake-center station) but without explicit permanent markers or with limited evidence of exact relocation.

Low fidelity: designs with explicit moving/variable locations (e.g., stratified random stations, trawl tows, voyages/track lines, opportunistic sightings, aggregated observer grids).

We then conducted a conservative sensitivity analysis by re-running the full modelling pipeline using only the High-fidelity subset (i.e., studies with explicit evidence of fixed sampling locations; n = 76 studies; 59% of all studies; see Supplementary Table 10). Results were qualitatively unchanged and key inferences remained consistent (see the Figure below). This suggests that our main conclusions are not driven by datasets with lower spatial location fidelity. Nevertheless, we acknowledge that the issue of spatial fidelity can be a mitigating factor that we now briefly discuss in the manuscript (Line 396–401) and while highlighting that it does not likely change the qualitative outcome of our results given in the sensitivity analysis

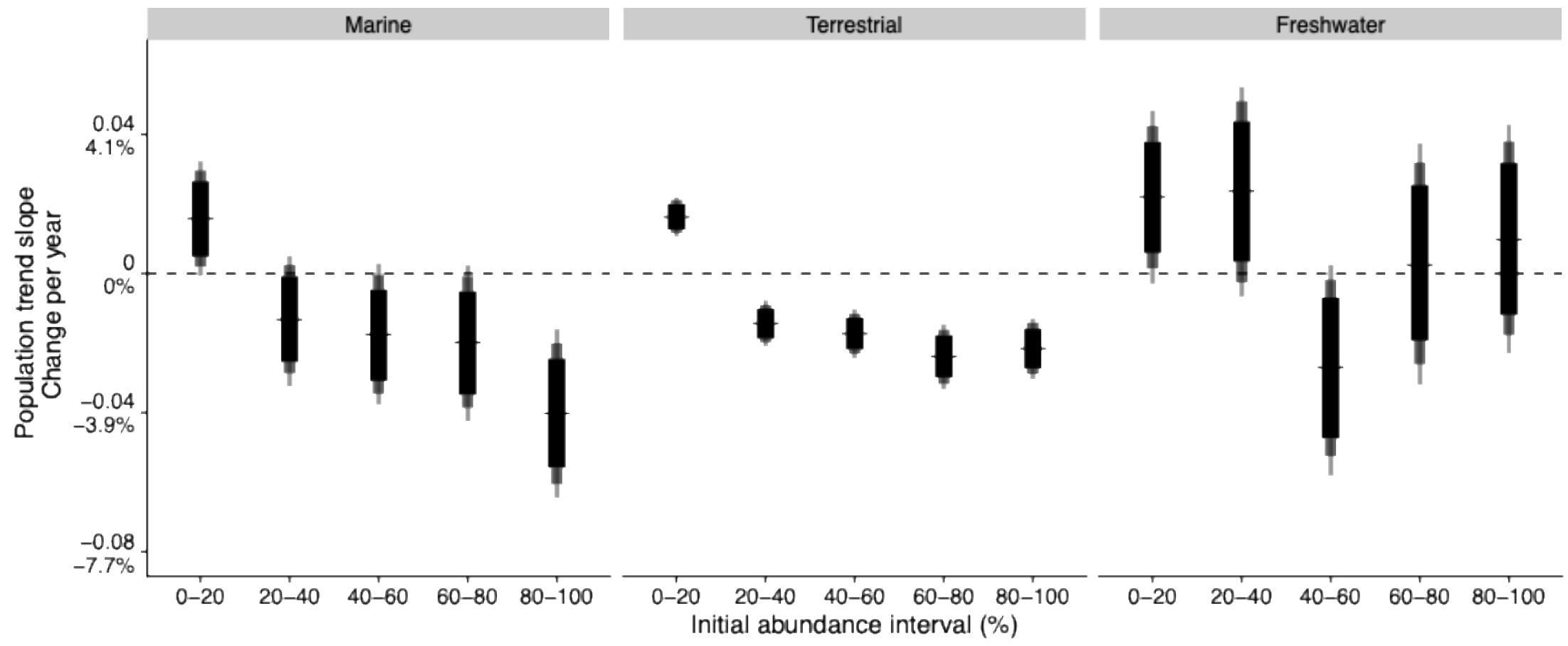

Line 60 – delete of

RESPONSE: Done

Figure 1 – (a) does not appear to have a common axis with (b) and (c). I think it therefore requires a separate axis label or at least more spacing to make this clear.

RESPONSE: We agree that the current layout could create ambiguity about the axis structure across panels. To address this, we have revised Figure 1 by adding explicit X-axis tick marks to panel (b) to match panel (c), making it clear that panels (b) and (c) share a common X-axis representing initial abundance intervals, and by increasing the vertical spacing between panel (a) and panels (b–c) to visually distinguish that panel (a) represents a different analytical unit from panels (b–c).

Figure 1 – the 80% and 95% confidence intervals are very hard to see in black. As this is a Bayesian model should these be labelled credible intervals (CRI) and not confidence intervals (CI)? They are not the same thing. As INLA is using approximate Bayesian inference I am not completely sure which is being used here. If so, this will need changing throughout the text.

RESPONSE: We have increased the height of Figure 1b and used lines with different transparency levels to represent different interval levels, making the distinction between credible intervals more apparent. We now use "credible intervals" for Bayesian model outputs throughout the manuscript where appropriate, while retaining "confidence intervals" for the OLS regression analyses in Figure 4 and Extended Data Figure 8, as these represent frequentist inference.

Line 145 – worth clarifying in the main text that 0-20% is a bin

RESPONSE: We have revised line 152 to read: "Species initially ranked in the rarest abundance bin (0–20%) showed strong evidence of increases..."

Line 167 to 168 – I don't quite follow this sentence can you please reword.

RESPONSE: We have revised lines 189–192 to read: "For example, if historically common species were living in more favorable conditions (as predicted by the abundance-center hypothesis), directional environmental change could disproportionately impact them by shifting to conditions that are less favorable, leading to steeper declines in previously abundant species."

Line 237 to 239 – I had to read this a few times. You may need to spell this out rather than using "this slope". The other option would be to assign the slope a parameter and refer to this.

RESPONSE: To avoid confusion, we have now explicitly defined the key slope in the text as the "abundance rank transition slope" and consistently used this term thereafter. The relevant sentences now read (Line 266–270):

"For each assemblage, we quantified the linear association between a species' population trend and its $\log_{10}$-transformed initial abundance, which we refer to as the "abundance rank transition slope". Assemblages with more negative abundance rank transition slopes had stronger disproportionate losses of the initially most common species"

Line 250 to 251 – I think this point needs highlighting more explicitly in lines 161 to 173. I was thinking this when reading it but didn't get this point coming through explicitly until now.

RESPONSE: We revised lines 184–200 to more clearly highlight this point as a central explanation for the observed asymmetry.

Specifically, we have strengthened the text to explicitly state that directional environmental change may systematically undermine the conditions that historically enabled more common species to be abundant.

Line 342 – I think it should be "where the" rather than "where there"

RESPONSE: We have corrected "where there" to "where the" in line 394

Line 343 to 345 – Although I agree with this, I think an equally important point is that more work is needed to understand existing data. In many cases we do not know enough about the gradients it spans and how these relate to a globally representative sample.

RESPONSE: We revised the text to emphasize the need to better understand both existing data and future monitoring frameworks. The sentence now reads (Line 401–

405):

"Resolving these uncertainties will require both better characterization of the global representativeness of existing time-series data with respect to environmental gradients, and integrating long-term time series with spatial monitoring frameworks that track biodiversity change across gradients of habitat change."

Line 613 – can the model formula be given rather than the inla code (which is available with other code). This will mean that non-inla users can still follow. Also, what model fit and specification checks were conducted – posterior predictive checks?

RESPONSE: We have substantially revised the model description section to improve clarity and accessibility. Specifically, we have made the following changes:

1. We have removed the INLA-specific code from the main text and replaced it with a clear, standard model specification using widely-recognized notation. The revised model for species-level trend estimation is now presented as:

Abundance ~ Realm × Year + (1|Study_ID) + (1|Taxon) + (1|Assemblage) + (1|Assemblage_Species) + (0 + Year|Study_ID) + (0 + Year|Taxon) + (0 + Year|Assemblage) + (0 + Year| Assemblage_Species) + AR1(Year|Assemblage)

This notation clearly indicates the fixed effects (Realm, Year, and their interaction), random intercepts (1|·), random slopes (0 + Year|·), and the temporal autocorrelation structure (AR1). We provide detailed explanations of each component immediately following the model specification.

2. We have explicitly stated that:
   Species abundance was modeled using a Negative Binomial distribution (rather than Gaussian with $\log_{10}$-transformation) to appropriately handle count data with overdispersion
   Community-level metrics (total abundance, species richness, Hill numbers) were modeled using Gaussian distributions with $\log_{10}$-transformation
   Pielou's evenness was modeled using a beta distribution with logit link function, given its bounded nature (0-1)

Regarding model diagnostics, we conducted residual checks for all five interval-specific models. We examined the distribution of Pearson residuals for each model, and all showed conformity to a normal distribution, indicating adequate model fit.

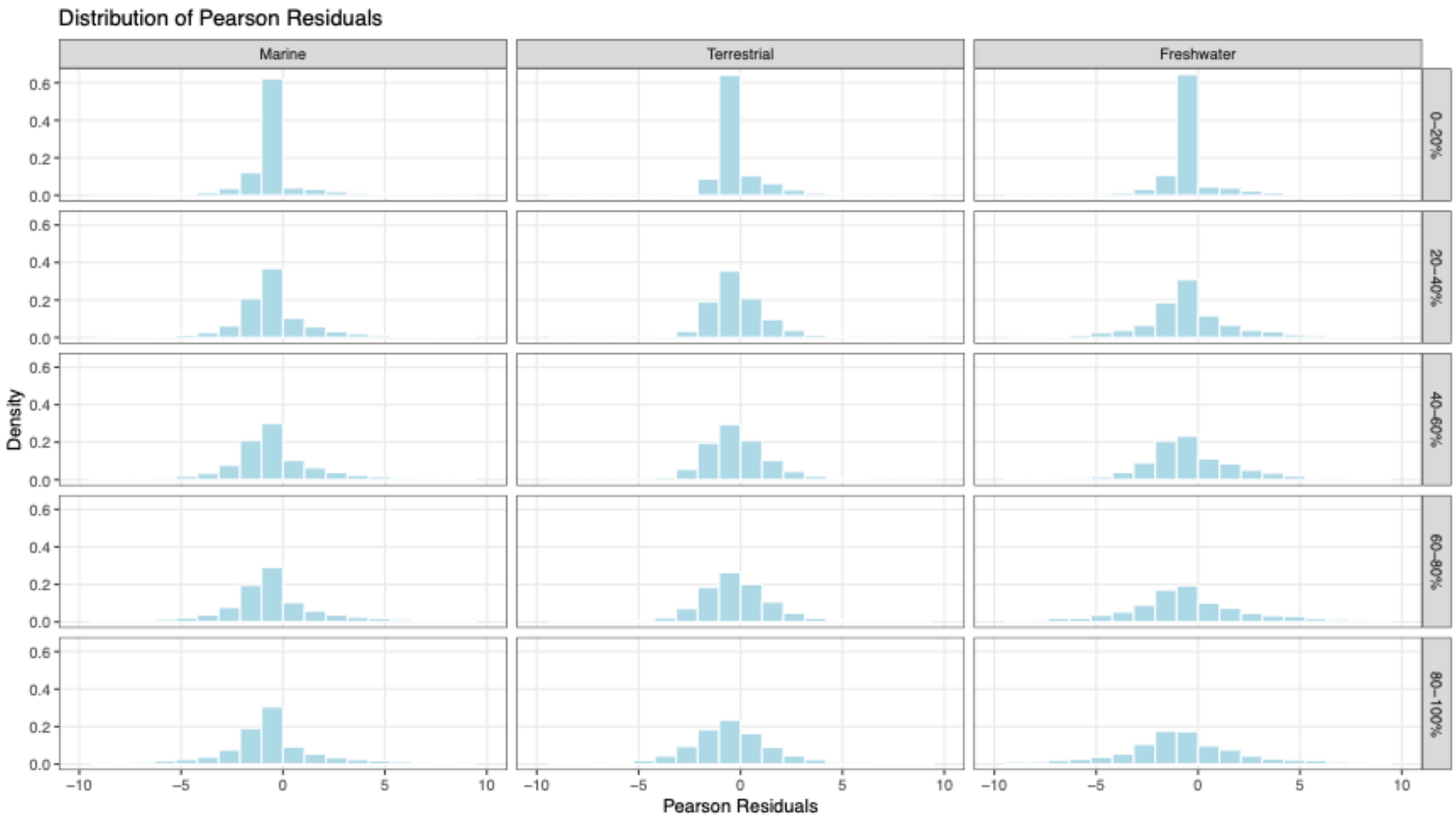

We believe these revisions improve the transparency and reproducibility of our analytical approach while making the methods more accessible to readers from diverse statistical backgrounds.

Reviewer #2 (Remarks to the Author)

This manuscript reports on an analysis of changes in (local) abundance across a relatively large number of taxonomic groups, with good temporal coverage and including marine, terrestrial, and freshwater systems. The major finding reported is a consistent decline across taxonomic group and realm (i.e., marine, terrestrial, freshwater) of common species, whereas rare species show increasing or flat trends.

The scope of the dataset is impressive, and the major conclusions are interesting and topical. The quantitative analysis is also sophisticated. However, I have several major concerns about the analysis, in particular in the way that rare species were chosen and in the interpretation of the Bayesian models. Taken together, I am not convinced that the analysis supports the conclusions, and I would need to see a significant reworking before I am convinced that the results are not an artifact of the methodology.

MAJOR CONCERNS

1. The analyses include time-series with leading zeros. The abundance trends of these 'populations' are therefore expected to be non-negative, i.e., worst case scenario, stable, no trend. At the same time, these 'populations' with leading zeros are categorised as rare species. I worry that this methodological decision artificially inflates the trend estimates for 'rare' species. These species definitionally cannot decline, and therefore will bias the estimation for rare species towards positive slopes.

Extended Data Fig 7 highlights this point – when removing species with leading zeros, rare species population trends are negative (although still less negative than those for common species). Van Klink et al 2023, which the current paper draws on heavily, excluded species-assemblage time-series with leading zeros for the reasons highlighted above. Unless the authors can strongly justify the inclusion of 'colonising' species (or missed detections), I would be in favour of presenting results excluding time-series with leading zeros, due to their more conservative nature. Further, the interpretation of these leading zeros has important ecological consequences. If they are 'true' zeros, such species represent colonising (not rare) species, whereas 'false' zeros or missed detections do suggest rare species. Linking back to Ext Fig 7, when ignoring initial zeros (and assuming these represent colonisation) rare species do decline. The paper could then conclude that colonising species increases are outweighing declines in 'native', rare species.

RESPONSE: We agree that the inclusion of time-series with leading zeros poses a methodological concern, as these species cannot exhibit declining trends by definition and may therefore bias rare species trend estimates upward. The reviewer points out that leading zeros could represent either “true” zeros (colonizing species) or “false” zeros (detection failures of rare resident species), which have fundamentally different ecological interpretations. Unfortunately, we cannot reliably distinguish between these two scenarios.

While one way to approach this is to exclude all time-series with leading zeros, as Van Klink et al. (2024), we feel that excluding these species biases the analyses away from the rarest species. In reality, the ‘truth’ of rare species is almost never possible to know. Indeed, one of the authors on this manuscript was senior author on van Klink et al. (2024), and those authors had the same discussion. While eventually agreeing to the final approach of removing rare species, it is clear that this does not eliminate bias, but simply puts it on the other side. So, while we appreciate the reviewers point and concerns, we do not agree that removing species with leading zeros is the most conservative approach. It is less biased in one way and more biased in another. These ‘rarest’ species could have been present in very low numbers, or only nearby but not present. However, mathematically, these are points on a continuum. If we had arbitrarily included all species that were zero as being present in very low numbers (i.e., one individual), marine rare species would still show a positive trend of 1.87% per year (see the Figure below).

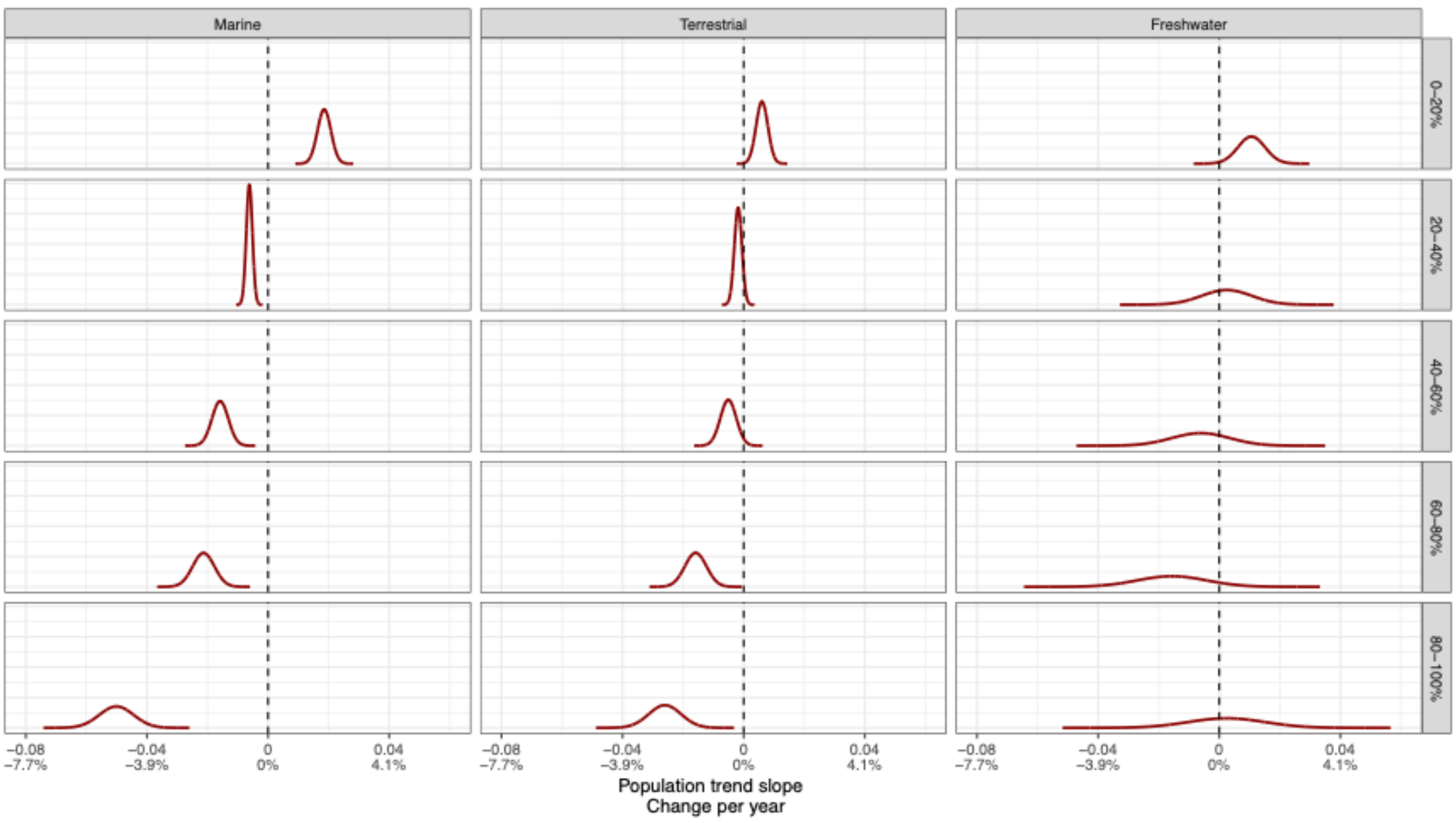

To acknowledge the potential influence of both sides of the potential bias, we have revised the manuscript to acknowledge both perspectives. Specifically, we now

explicitly state that leading zeros may represent either true colonization events or detection failures of rare resident species, and that these two interpretations have distinct ecological implications. We also clarify that excluding time-series with leading zeros, while addressing one source of bias, introduces another by systematically removing the rarest species from analysis. This balanced framing is now reflected in both the Methods and Discussion sections.

2. Using the log(y + 1) as the response variable is a potentially problematic choice, as is the inclusion of species with zero initial abundance as "rare" species. These species are not rare at the local scale, they are absent, and they can only increase from there. But your statistical model assumes they are rare by adding one for statistical convenience. On the log scale this is an infinite increase with no meaningful interpretation! Although it is a common practise to add a small number to log-gaussian models in order to account for observed zeros, in a dataset with many zeros this has non-trivial implications for estimation (adding one to an observed zero is much more impactful than adding one to a large number). It is more common in Bayesian models to incorporate a likelihood structure that more closely reflects the statistical process generating the data. In this case, a Poisson or Negative Bionomial likelihood function would seem to be appropriate for abundance data, possibly with a finite mixture to account for many processes that can generate zeros (e.g., true absence, low abundance leading to nondetection, etc).

RESPONSE: We agree that log(y + 1) transformation is potentially problematic for count data with many zeros. Following the reviewer's recommendation, we have re-analyzed our data using both Poisson and Negative Binomial distributions and adopted the Negative Binomial model as our primary analytical framework, as our data exhibit substantial overdispersion and the Negative Binomial model consistently shows lower WAIC values than the Poisson model. We present the results from the Poisson for completeness and transparency in the Supplementary Table 9.

Prior to model fitting, we addressed a convergence issue caused by extreme abundance values (maximum >70 million, primarily from marine plankton studies). We removed entire time-series if any data point exceeded the 99.5th percentile (~67,000) of all non-zero abundances, which excluded 520 time-series. Additionally, we excluded four marine time-series due to erroneous latitude and longitude coordinates identified during data validation, resulting in a final dataset of 4,748 time-series.

In all, the Negative Binomial model results are broadly consistent with our previous log(y + 1) Gaussian models. Common species show strong evidence of decline in marine (9.51% per year) and terrestrial (2.70% per year) realms. These revised

estimates, which incorporate a regression-to-the-mean correction factor, are presented in the updated Figure 1b. We have revised the Methods section accordingly and updated all Figures and tables to reflect the Negative Binomial modeling framework.

3. The paper's current approach to assigning species to 'initial abundance bins' per site is based on the maximum (log) abundance value across the entire time-series. I believe this will lead to an artificial reduction in the trends of the most common 'assigned' species – if their abundance is >80% of the time-series max in the first year of the time-series, they are likely to subsequently decline. Again, Extended Data Fig 4 suggests this occurs, left truncation (of even 1 year) reduces Marine population trends to ~0 for all abundance bins, with the most 'common' species displaying positive trends, on average after left truncation. I would favour a more conservative approach for assigning species to abundance bins. Specifically, either i) using (log) abundance values for the initial year, and creating bins based on the maximum of these values (Van Klink et al, 2023), ii) using average (log) abundance values for the first 3 to 5 years (per species), and creating bins based on the maximum of these values, or iii) most conservatively, using average (log) abundance values for the entire time-series (per species), and creating bins based on the maximum of these values.

RESPONSE: We agree that our previous classification method could artificially reduce the trends of common species, as the reviewer noted. Species with peak abundance early in the time-series would be disproportionately assigned to the "common" category, potentially biasing trend estimates for common species toward negative values.

Following the reviewer's recommendation, we have adopted option (i): classifying species based on initial-year abundance (Van Klink et al. 2024). Species are now categorized according to their abundance in the first year relative to the maximum first-year abundance within the same local assemblage.

The primary patterns remain largely consistent. In marine and terrestrial realms, common species continue to show stronger declines than rare species. In freshwater systems, the results shifted: neither common nor rare species show clear directional trends. This pattern in freshwater may reflect the smaller sample size in this realm compared to marine and terrestrial systems, though it may also correspond to a genuine ecological trend.

To demonstrate robustness, we conducted sensitivity analyses using the reviewer's other suggested classifications: classifying species based on (ii) mean abundance

over the first three years and (iii) mean abundance over the first five years, (Extended Data Fig. 5). Our main conclusions remain qualitatively consistent across all three classification schemes.

We have updated the Methods section accordingly, and revised all relevant figures and tables.

A further concern, although hard to remedy, is that allocations of abundance classes are based on per assemblage relative abundances. It's possible that in two different assemblages, a species with similar (or even the same) absolute abundances could be categorised as rare in one but common in the other – depending on the other species present. Alternatively, if an assemblage consists of only objectively (low absolute abundance) rare species, all could be categorised as common. Is there an alternative approach, based on regional, or study-level measure of rarity or similar, that could be used to test the robustness of these abundance-class assignments?

RESPONSE: We agree that a species with similar absolute abundances could be categorized differently across assemblages, or that assemblages composed entirely of objectively rare species could have all species classified as "common" within that assemblage. At the same time, however, we want to emphasize that our primary question is about local commonness and rarity (i.e., the shape of a local species abundance distribution; SAD), which is different than a question about changes of rare and/or common species regionally (or globally). That is, our focus is on within-assemblage dominance hierarchies and community reorganization, rather than tracking population trends based on species' absolute abundances or geographic distributions.

Regarding alternative approaches using cross-assemblage or regional-level measures of rarity, we note that Xu et al. (2023, Nature Communications; https://www.nature.com/articles/s41467-023-37127-2) conducted such an analysis using occupancy-based metrics across assemblages using a similar version of the dataset. Their approach, which defines rarity/commonness based on geographic range and cross-site occurrence frequency, complements our within-assemblage perspective and addresses a different ecological dimension.

While alternative definitions of commonness and rarity to analyze population trends would be a valuable complementary approach this is beyond the scope of the questions we set out to test. We believe this represents an important direction for future research using appropriately structured datasets.

4. I am concerned about the precision and accuracy of the tails of the posterior distributions, and in general about the influence of extreme values on estimation and

inference. INLA estimates an approximate posterior distribution, and such approximations will necessarily be more accurate near the centre of the distribution. Moreover, using only 1000 samples from the posterior distribution means that only 50 samples are available for estimating the tails of a 95% credible interval, and only 25 for determining whether a given interval overlaps zero, which is critical to the interpretation of a "strong" effect in the ms. Given the importance of tail estimation to the conclusions, at a minimum, the authors should attempt to quantify tail uncertainty, and to refine the methods to improve tail sample size and precision.

Moreover, the figures in the ms consistently exclude extreme values. While this can improve visualisation by reducing the scale on the axes, it also makes it impossible to evaluate whether such extremes might influence parameter estimates. I find myself wondering what values are being excluded from the figures, and in what direction they influence the slopes. I think it is essential to include figures with all data points, if not in the main ms then at least in the SI.

RESPONSE: We have addressed this concern through the following revisions:

(1) We increased the number of posterior samples from 1,000 to 4,000, providing 100 samples per tail (2.5% each side) of the 95% credible intervals.

(2) To assess tail behavior and the precision of posterior estimates, we compared the INLA marginal density curves with the empirical distributions derived from 4,000 posterior samples. For each model, we extracted the marginal distributions for fixed slopes across all three realms—marine (as the reference category), terrestrial, and freshwater (the latter two obtained via linear combinations specified in the model). As shown in the figure below, the density estimates from the 4,000 posterior samples (steel blue shading) generally align with the marginal density curves (dark red lines), with reasonable agreement in the tail regions. Based on this, we consider the tail estimates from 4,000 samples to be acceptable and sufficient to support the precision requirements for inference on the 95% credible intervals.

A more rigorous validation approach would involve comparing INLA results with those from Markov Chain Monte Carlo (MCMC) sampling. However, given the substantial size of our dataset and the complexity of the hierarchical model structure, MCMC estimation would require prohibitively long computation times, making this approach impractical for our analysis.

(3) We have now displayed all extreme values in the figures (previously truncated for visual clarity), ensuring complete transparency of the data distribution.

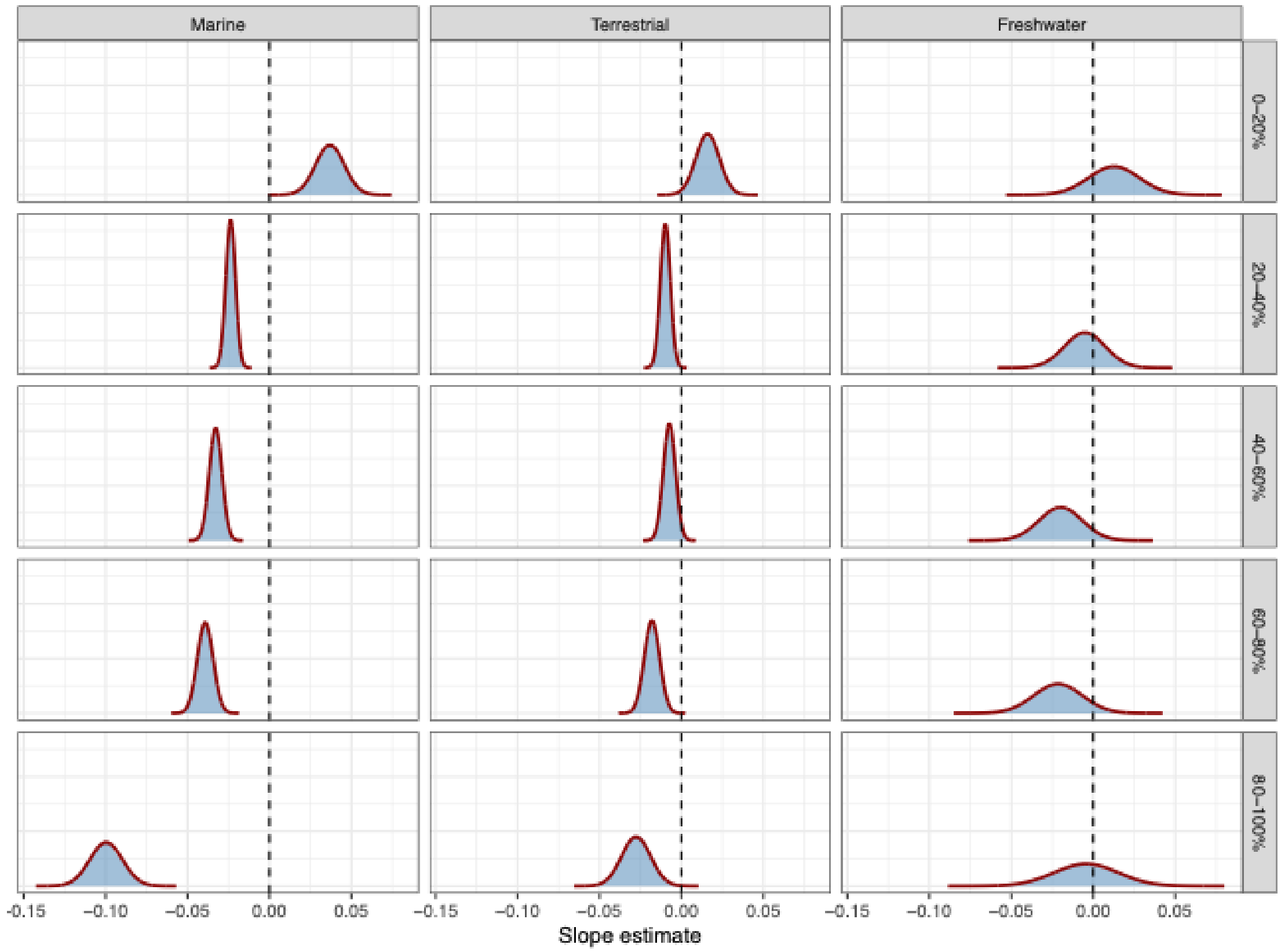

Line 2: The title emphasises declines in common species, yet Fig 1c indicates that rare species contribute most to the assemblage level changes in total abundance (for Marine populations at least). I think maintaining consistency in your narrative is important for the reader to follow your arguments/conclusions.

RESPONSE: We thank the reviewer for highlighting this inconsistency. As detailed in our response to a related comment below, we have revised the calculation method for Fig. 1c. With this correction, the results no longer indicate that rare species consistently contribute most to assemblage-level changes in total abundance. We have revised the corresponding text in the manuscript to ensure consistency between our narrative and the updated findings.

L35: Although the data are impressive, I think the geographic biases prevent statements about ‘global erosion of formerly common species’. ‘Widespread declines in common species’ or similar may be more appropriate.

RESPONSE: We have revised the statement as suggested: “global erosion of formerly common species” has been replaced with “widespread declines in common species” to avoid inaccuracies caused by geographic biases.

I also think the term ‘erosion’ is very strong, given the small-ish number of ‘common’ species (relative to rare) in this analysis (e.g., 295 common v 5625 rare for Terrestrial).

RESPONSE: We have removed all instances of "erosion" from the manuscript.

L52: Do you mean abundances of individual species, or total assemblage abundances?

RESPONSE: Here "abundances" refers to individual species abundances. We have revised the text to make this explicit.

L60: ‘of across’ – I think a word is missing. Abundance?

RESPONSE: We have revised the sentence to clarify the meaning.

Line 62: Temperature is not a resource. Heat or energy perhaps, but not temperature.

RESPONSE: We have revised "resources (including temperature)" to "environmental conditions (including temperature)".

Fig 1: I found the lack of x-axis labels in row b confusing -- rows b and c share an x-axis scale, but not row a, and this was not obvious. I suggest repeating the axis labels in row b for clarity.

RESPONSE: We have added x-axis labels to row b for clarity.

L64-66: The final sentence of this paragraph largely repeats lines 50-54.

RESPONSE: We have removed this sentence to avoid repetition.

L69-70: Which populations are emphasised in the mentioned indicators/data? An indication of some of the biases, currently not stated, would be useful for the reader to know what specifically you are commenting on. Geographic bias? Taxonomic focus on vertebrates?

RESPONSE: We have revised the sentence to specify the bias, noting the taxonomic focus on vertebrates: "Prominent indicators, such as the Living Planet Index and related analyses, emphasize trends in selected populations, but have a number of potential biases (e.g., taxonomic focus on vertebrates) that complicate inference about how biodiversity change is distributed across species ranks within an assemblage."

L97: What do you mean by ‘species-level’? The model estimates overall trends by realm, and for each random effect, but there isn’t a ‘species-level’ random effect, only a ‘species-time series’ one. This is finer that species-level, in my opinion.

RESPONSE: We thank the reviewer for this clarification. We have revised all instances of "species-level abundance trends" to "species-time series level" throughout the manuscript.

L102-103: The inclusion of taxonomic group as a random effect is already stated on L98.

RESPONSE: We have removed this sentence to avoid repetition.

L105: I would consider climate/temperature change a human impact. And the reference used for human impacts incorporates climate change (although I know you remove it). Clearer distinction/naming between the two data types would be useful, e.g. 'Climate change' and separately, 'non-climatic drivers'.

RESPONSE: We agree that climate change is also a form of human impact. We have revised the terminology throughout the manuscript, now distinguishing between "climate change" and "non-climatic anthropogenic drivers" to provide a clearer distinction between the two data types.

L111: 0.0038 is a small number, even if the CIs do not overlap 0. Could you contextualise this number? e.g. % population change, expected loss/gain in a population of 100 individuals over a given time-period (one year, or maybe a decade). Van Klink et al. 2023 present these values a both 'trends', and % population change per year, which I think helps the reader better interpret the results.

RESPONSE: We thank the reviewer for this suggestion, which was also raised by Reviewer #1. Following van Klink et al., we have added percentage change per year to help contextualize effect sizes. With the updated analysis (including additional data-screening rules; see second Major Concern for details), the marine total-abundance slope is now 0.0032, corresponding to an annual increase of approximately 0.73%.

L131: There also appear to be some negative peaks in the distribution for marine total abundance trends. Given this work focuses on the nuances of biodiversity change, it would be nice to acknowledge these apparent declines in assemblage-level abundance – and potentially discuss them and their possible causes.

RESPONSE: We disaggregated the time-series-level abundance trends by taxonomic group (see Extended Data Fig. 2 below). This analysis revealed that the negative peaks in marine systems are primarily attributable to the "Multiple" category—studies that simultaneously sampled several different taxonomic groups. Due to the composite nature of these multi-taxa studies, it is difficult to attribute the observed declines to any specific taxonomic group within them.

To address this point, we have added the following sentence to the main text following the description of Figure 1a (Line 108–112):

"Despite these overall positive mean trends in marine assemblages, the distribution of time-series-level changes revealed considerable heterogeneity. Negative trends were observed primarily in multi-taxa studies (Extended Data Fig. 2), suggesting that single-taxon surveys may miss broader community-level declines from multiple taxa."

Regarding the potential causes of these declines, we note that our central finding—the disproportionate decline of formerly common species—was observed consistently across assemblages regardless of whether total abundance was increasing or decreasing (Figure 1b). We have therefore chosen to focus our mechanistic discussion on this overarching pattern rather than on the specific causes of decline within particular subsets of time series, though we recognize this remains an interesting avenue for future investigation.

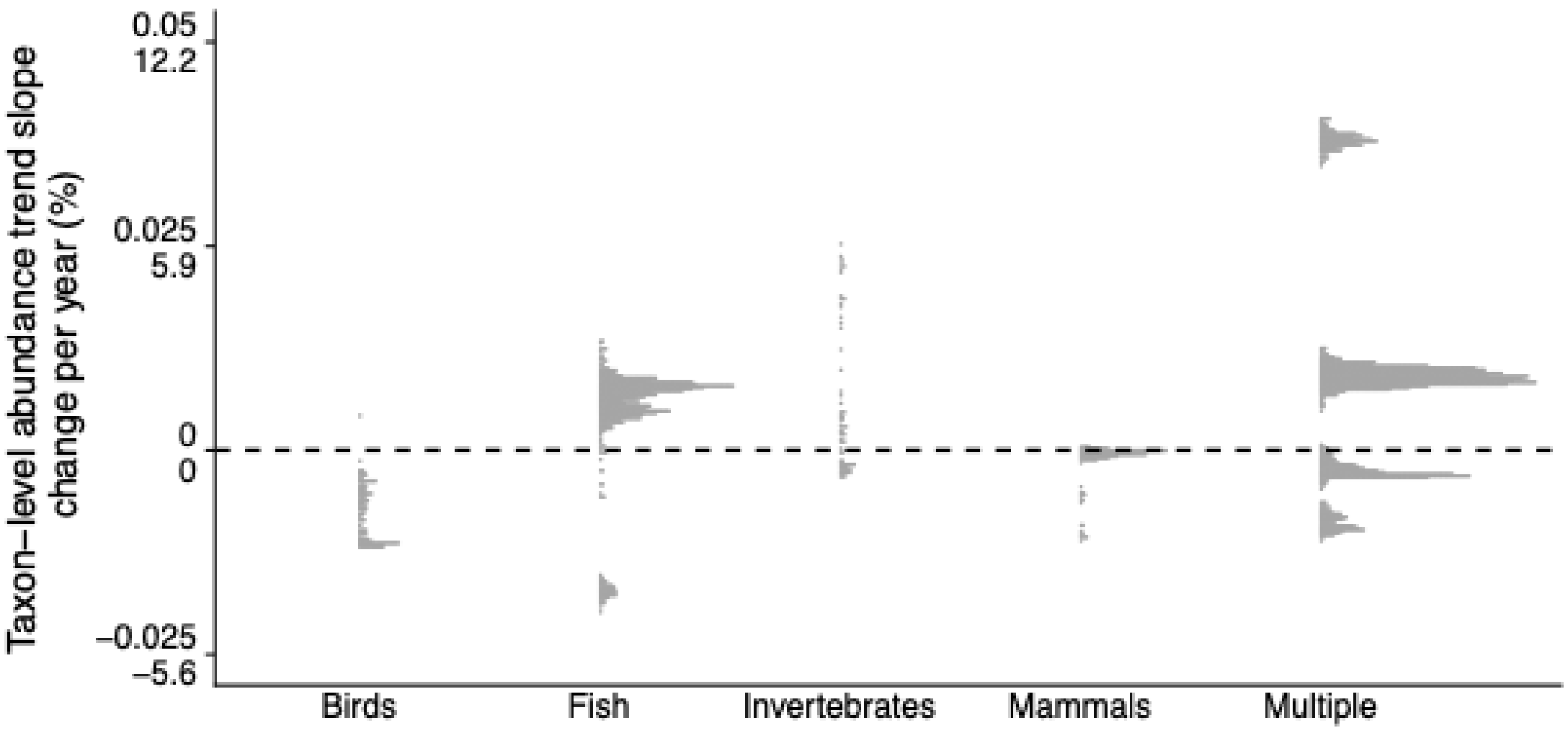

L135: I think '90%' is missing in the list of CI widths.

RESPONSE: We have supplemented "90%" to the list of CI widths as suggested.

L136-141: 'Common species contribute most…' – I am a bit lost as to where this statement should go/what it relates to. Total abundance? In Fig 1c, and the below sentence, you state that "Rare species contribute more than expected on a per-abundance basis". Although the largest (absolute) contribution in freshwater are the two most common abundance classes. In this context, what would the expected contribution be? 20%? But see a later comment about accounting for number of species present per abundance class.

RESPONSE: Our apologies that the original description was unclear. The statement

"Common species contribute most" in Figure 1b was intended to refer to the absolute contribution to total abundance change, but this was misplaced and poorly worded.

We have revised the figure legend as follows (Line 136–146):

"(b) Population trend estimates per initial abundance interval, corrected for regression-to-the-mean effects. Black horizontal bars and error bars represent the mean, 80%, 90% and 95% credible intervals from 4,000 posterior samples of fixed term. In marine and terrestrial realms, common species show more pronounced declining trends than rare species. (c) Proportional contribution of each abundance bin to total abundance change. Error bars represent 95% credible intervals of mean contribution. In marine systems, the most common species contribute more than the rarest ones; in terrestrial systems, the rarest species contribute more than the most common ones; and in freshwater systems, contributions from the rarest and most common species are nearly equivalent."

L154: What are the scales of abundance values involved in defining rare to common species? E.g. average initial abundance per 'bin'. Do these distributions shift substantially across Realms? Across time-series (sites), studies?

RESPONSE: We report the distribution of initial abundances for each abundance interval across realms, with mean and median values indicated (see figure below). Despite the diversity of taxa and sampling methods in our dataset, we find that absolute abundance scales remain broadly comparable across realms within the same abundance interval. For example, in the 80 – 100% interval, abundance values largely fall within the 10 – 1,000 individual range across all three realms, with median values showing even greater convergence. Mean values differ more substantially due to their sensitivity to extreme values, but the overall distributions largely overlap.

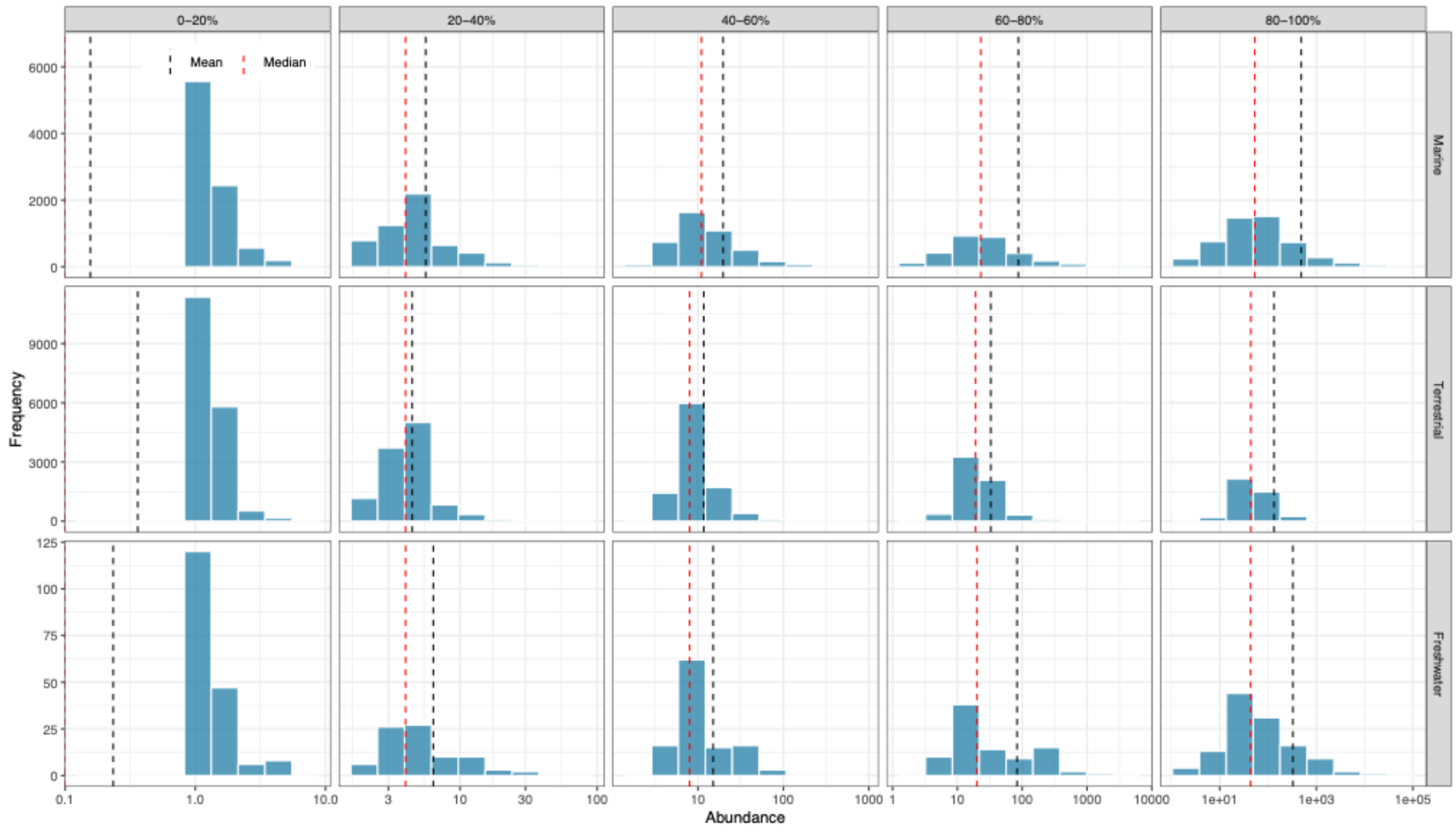

Regarding variation across time series and studies: differences in absolute abundance scales do exist, as some assemblages naturally support higher species abundances depending on taxa, sampling methods, and local environmental conditions. However, as we noted in our response to the reviewer's earlier comment (see MAJOR CONCERN #3), the core objective of this study is to examine whether species occupying the high-abundance end of the species abundance distribution within local assemblages exhibit different temporal dynamics compared to locally subordinate species. By using relative (percentile-based) classifications within each assemblage, we quantify each species' position in the local dominance hierarchy. We believe this approach enables meaningful comparisons of abundance-trend relationships across ecosystems that differ in their absolute abundance scales.

L158: ‘due’ – I am not sure this word is needed.

RESPONSE: We have removed the redundant word "due".

L166-168: Why would species near the center of their range be more exposed to environmental pressure?

RESPONSE: Our intended meaning is that if common species are concentrated in habitats that historically represented optimal conditions, directional environmental change may disproportionately impact these formerly favorable habitats, leading to steeper declines in previously abundant species. We have revised this sentence for clarity:

"For example, if historically common species were living in more favorable conditions (as predicted by the abundance-center hypothesis), directional environmental change

could disproportionately impact them by shifting to conditions that are less favorable, leading to steeper declines in previously abundant species."

L183: I would interpret Ext Data Fig 5 as suggesting weak support (80% CI) for changes to Hill numbers and Evenness in Marine and Terrestrial realms.

RESPONSE: This has been corrected.

L215-216: I'm not sure your results do suggest that 'most assemblages were previously structured around some formerly common species that are no longer so'. They suggest common species have declined, whilst rarer species have increased, but the magnitude of these changes appears small. It is possible that formerly abundant species are still the most abundant within that assemblage. I think context relating to the initial abundance values, and % change through time would help the interpretation of this. As a further test you could categorise species as rare/common based on their end/last 5 abundance values. If these categories differ from their initial abundance assignments, you would have more support for your conclusion of substantial changes in rank abundance. However, if they remain largely unchanged, your statement would need to be adjusted accordingly.

RESPONSE: Following the reviewer's recommendation, we classified species into abundance intervals based on their mean relative abundance during the first five years and the last five years of each time series, and examined transitions between intervals (see Supplementary Table 7 below).

We found substantial changes in species' positions within the abundance distribution. Among species initially in the 80 – 100% interval, only 54% remained in that interval by the end of the time series, with 46% transitioning to lower abundance intervals. Similarly, species in the 60 – 80% interval showed only 34% retention. In contrast, species initially in the 0 – 20% interval showed higher retention (83%), though 17% moved to higher intervals. We believe these results support the conclusion that community structure has undergone considerable reorganization over time.

	Final classification				
Initial classification	0 – 20%	20 – 40%	40 – 60%	60 – 80%	80 – 100%
0 – 20% (n = 139,815)	83.20%	11.32%	3.34%	1.16%	0.98%
20 – 40% (n = 31,534)	45.41%	31.60%	15.70%	4.77%	2.52%
40 – 60% (n = 18,600)	20.84%	23.96%	32.52%	16.69%	5.99%

60 – 80% (n = 11,223)	11.98%	11.98%	23.98%	33.89%	18.18%
80 – 100% (n = 10,180)	11.47%	7.99%	10.88%	18.02%	51.64%

Species-time series combinations were classified into abundance intervals based on their mean relative abundance during the first five years (rows) and the last five years (columns) of each time series. Values represent the percentage of species in each initial interval that transitioned to each final interval. Diagonal values indicate species that remained in the same interval.

L228-229: The declines in marine species presented here (Fig 2b) are substantially greater than those in Fig 1b? Why? How? Aren't these outputs from the same model, but re-organised to reflect taxonomic variation? Have the estimates been corrected for regression to the mean?

RESPONSE: As the reviewer noted, these results are from the same model but represent different hierarchical levels: Fig. 1b shows the fixed-effect slope posterior distribution, while Fig. 2b shows the combined posterior of fixed slopes + TAXA-level random slopes. The inconsistency in the previous version occurred because we inadvertently omitted the regression-to-the-mean correction factor for Fig. 2b, and we apologize for this oversight. We have now updated all results using the negative binomial model with an increased number of posterior samples. All estimates have been corrected for regression-to-the-mean effects, and the mean values are now on the same order of magnitude.

L236: Should this read 'steeper negative slopes'? Steep positive slopes would suggest initially common species doing proportionately better.

RESPONSE: The reviewer is correct. We have revised Line 269, replacing "steep slopes" with " more negative abundance rank transition slopes" to align with the intended meaning.

L245-247 and L261-262: The positive relationship in Fig 3 also suggests that communities experiencing net gains in abundance exhibit steeper (positive) trend-abundance relationships (although less substantial).

RESPONSE: The reviewer is correct that the positive relationship in Fig. 3 implies patterns on both sides of the relationship: communities experiencing net declines show steeper negative trend-abundance associations, while communities experiencing net gains show steeper positive trend-abundance associations (though less pronounced). We have revised the relevant sentences to more accurately reflect this bidirectional pattern:

Revised (Line 279–282): "In contrast, assemblages that were either stable or gaining total abundance through time showed weaker to positive trend-abundance associations (though less pronounced than the negative associations observed in declining communities)."

Revised (Line 293–296): "The fitted line shows a significant positive relationship (slope = 2.436, 95% CRI: 1.618, 3.255), indicating that communities experiencing net declines exhibit steeper negative trend-abundance associations, while those experiencing net gains show positive but less pronounced associations."

L264-265: Would the reasoning not be the other way around? i.e., trends in total assemblage are contingent on species-level changes.

RESPONSE: The reviewer is correct—total assemblage trends are the result of species-level changes, not the other way around. We have revised the sentence accordingly: "This suggests that shifts in species trends are not uniform, and the magnitude of these species-time series level changes collectively determines whether the total assemblage gains or loses individuals."

L282-283: Fig 4 suggests that warming and human impact are associated with 'more positive' trends in common species, not necessarily actual positive trends. Fig 2 suggests most/all common species declined in the marine realm, an association of warming with actual positive trends seems unlikely.

RESPONSE: We have revised "positive trends" to "attenuated declines" accordingly.

L316: Your results indicate a strong signal of change, however the magnitude of change appears small, rather than strong. Again, clarity regarding the interpretation of population trend estimate magnitudes would be useful.

RESPONSE: We have revised "strong declines" to "systematic declines" accordingly.

L331-334: Equivalently, regionally widespread species, can be locally rare – e.g. large carnivores. I wonder if a comparison between range-based commonness and your abundance-based allocation could be done? Would they align?

RESPONSE: We thank the reviewer for this suggestion. Xu et al. (2023, Nature Communications) examined range-based commonness and found that large-ranged species tended to increase in occupancy while small-ranged species tended to decrease. Our current study, based on local abundance, found the opposite pattern: initially common species declined while initially rare species increased. This contrast suggests that the two definitions of commonness can yield opposite temporal trends, likely because the relationship between local abundance and regional distribution is

complex and not consistently positive.

L556: How many time-series contain constant species abundance? Is this a data error?

RESPONSE: A total of 56 time series had species abundance constant at 1 throughout the entire period. We do not consider these to be data errors, as they likely reflect real observational cases where focal species maintained stable, low abundance over the monitoring period. However, these cases were excluded because constant singleton abundances prevent meaningful calculation of relative abundance distributions and classification into abundance intervals. We have added this clarification in the revised manuscript (Line 617).

L569: Do ‘rarefied samples’ refer to the median of rarefaction samples? Or individual samples?

RESPONSE: Biodiversity metrics were calculated for each of the 199 individual rarefied samples, and the median value across these iterations was then recorded. We have clarified this in the revised text:

Revised (Line 637): "For each of the 199 rarefied samples, we calculated total abundance, species richness (*q*=0), Hill number for Shannon (*q*=1) and Simpson (*q*=2), and Pielou's evenness, and then recorded the median value of each metric across iterations."

L587: Does ‘20 years of sampling data’ refer to 20 sampling time-points, or a total time-series length of 20 years?

RESPONSE: This refers to 20 sampling time-points. We have revised "20 years of sampling data" to "20 sampling time-points" accordingly (Line 664). We also corrected a similar ambiguity in the data filtering criteria (Line 610), revising "time series samples spanning at least 10 years" to "time series with at least 10 sampling time-points".

L590: Clearer description of your correction factor approach would be helpful. Is there a correction factor per realm:taxon combination?

Does using long time-series under-estimate potential correction factors? Long time-series are typically more stable than shorter ones.

RESPONSE: Correction factors were calculated separately for each realm, with all taxonomic groups within the same realm sharing the same correction factor. We have clarified this in the revised methods (Line 668).

Regarding the second point, the reviewer is correct that long time-series are typically more stable. We used long time-series following the methodology of Van Klink et al 2024, as they provide more reliable estimates of the correction factor. This approach is conservative: if long time-series yield smaller correction factors, we are under-correcting rather than over-correcting, meaning any significant patterns that persist after correction are more robust

L610: Do you test model residuals for spatial/phylogenetic correlation?

RESPONSE: We agree that testing model residuals for spatial and phylogenetic correlation is important, because unmodelled dependence can inflate effective sample size and lead to underestimated uncertainty. However, for the reasons below, we did not include these analyses in the current version.

Spatial correlation:

We addressed spatial non-independence by incorporating a hierarchical random-effects structure (Study ID and Assemblage) within our INLA models. This approach is specifically intended to account for the clustering of sampling units, which represents the dominant source of spatial autocorrelation in the BioTIME dataset (Antão et al., 2020). By fitting random intercepts and slopes for these spatial units, we capture shared environmental and methodological variation within localized regions, thereby reducing the risk of inflated effective sample sizes. Although this hierarchical random-effects framework effectively controls for the main within-group spatial clustering and substantially mitigates pseudo replication, we acknowledge that it may not fully eliminate all forms of residual, distance-based spatial autocorrelation. For this reason, we did not perform an explicit residual spatial correlation test, and we have added a sentence to the Methods (Line: 686) to clarify this modelling choice and its limitations.

Phylogenetic correlation:

We also agree that phylogenetic signal in population dynamics is plausible, and that explicitly modelling phylogenetic (as well as spatial/temporal) dependence can increase uncertainty and, in some cases, alter inferred trend direction (e.g., Johnson et al. 2024). At the same time, the magnitude—and even presence—of phylogenetic signal is context dependent, and does not appear to be consistent across response variables or scales.

In particular, (1) whether abundance/rarity (e.g., common vs. rare status) shows phylogenetic signal remains debated across systems (e.g., Pie et al. 2021; Loza et al. 2017; Veldhuisen et al. 2025), and (2) whether responses to environmental

change/disturbance or decline show phylogenetic signal is also not universally supported (e.g., Lavergne et al. 2013; Martínez-Blancas et al. 2018; Thomas 2008). Therefore, incorporating phylogeny would not be expected to shift our results in a single predictable or easily interpretable direction, although—where phylogenetic dependence is present—it could make inference more conservative by appropriately inflating uncertainty.

Based on the considerations above, we did not test model residuals for phylogenetic correlation in the current analysis, nor did we fit a phylogenetic covariance structure. We have now explicitly acknowledged this limitation in the manuscript (Line: 690) and clarified that unmodelled phylogenetic dependence—where present—could lead to underestimated uncertainty and should be considered when interpreting our inference.

L627: Is a gaussian error distribution suitable for Evenness (bounded 0-1)? Did you check model residuals?

RESPONSE: This is an important point. Gaussian distribution is not ideal for Pielou's evenness, which is bounded between 0 and 1. We have re-fitted the evenness model using a Beta distribution, which is theoretically appropriate for continuous proportional data. The results remained consistent with our original findings. We have updated the Methods section accordingly (Line 711).

L636-641: Are abundance change slopes (s) in log-10 space? Do these need to be transformed before estimating N. Or is N in log-10 space?

Your code suggests the use of values on the log10 scale, but this is not clear in the methods.

RESPONSE: Thank you for highlighting this lack of clarity. In the original manuscript, both the abundance change slopes (s) and abundance (N) were valued on the $\log_{10}$-scale. In the revised analysis we fit a negative binomial model with a log link, so the estimated temporal slopes (s) are on the natural-log (ln) scale of the expected abundance (i.e., slope of log μ per year). We have explicitly clarified this detail in the Methods section and revised the corresponding notation accordingly. Importantly, abundance changes (ΔN) are now uniformly calculated on the natural-log scale. Notably, log10(abundance+1) is used only for defining initial-abundance bins (for grouping), not for estimating ΔN.

How does use of log-space affect weighting of rare vs common species? I suspect it (relatively) upweights the contributions of rare species. Are you interested in additive abundance change, or relative/multiplicative abundance change? I thought the

former, given your description of N etc, but the use of log10 space would suggest the latter.

RESPONSE: Our objective is relative change in total abundance (i.e., the ratio or fold change in individual counts, for instance, a 50% increase from 100 to 150 individuals), rather than additive change. We think that additive change measured on the original count scale is not appropriate, as the absolute abundance changes of common species are inevitably much larger than those of rare species, which would obscure the contribution signals of rare species. We now directly use the predicted values output by the model for calculations. Specifically, for each species time series and posterior draw, we compute ΔN as the difference in expected abundance between the last and first observed years ($\Delta N = \mu_{last} - \mu_{first}$), and this calculation is performed entirely on the natural-log scale derived from the negative binomial model with a log link.

Your N calculation accounts for the initial abundance of a species, which you then sum within each abundance interval to generate Ci. Unless I have misunderstood, your values of Ci are therefore affected by the total number of species in each interval – and this is highly uneven, there are many more rare species than common ones and so their expected contribution would be higher (e.g., 3162 rare Marine species v 207 common ones). I think you either need to define an expected contribution per abundance class, accounting for initial abundance and number of species per class, or estimate the 'average' contribution of each abundance bin (i.e., controlling for number of species in the calculation of Ci). I think both would be more informative than the current measure.

Sites also vary in their time-series lengths which affects their N value. The relative distribution of abundance intervals across time-series lengths can therefore also affect N and Ci values. For example, a particularly long time-series may, by chance have many, or few rare species. I would assume these influences average out, but it is possible that they don't. Maybe using a standard time-series duration would better control for this?

RESPONSE: Thank you—this helped us improve both interpretation and robustness checks. We have revised the calculation of the contribution metric for each abundance interval. Specifically, we now compute C_i as a unified metric that simultaneously accounts for both the highly uneven number of species-time series across abundance bins and the heterogeneity in time-series duration, with the formula $\bar{C}_i = \frac{1}{n_i}\sum_{s \in i}(\frac{\Delta N_s}{T_s})$, where n_i is the number of species-time series in the

i-th abundance interval, ΔN_s is the abundance change of the s-th species-time series, and T_s is the duration of that corresponding time series.

The results after these modifications are as follows: In marine systems, the most common species contribute more than the rarest ones; in terrestrial systems, the rarest species contribute more than the most common ones; and in freshwater systems, contributions from the rarest and most common species are nearly equivalent.

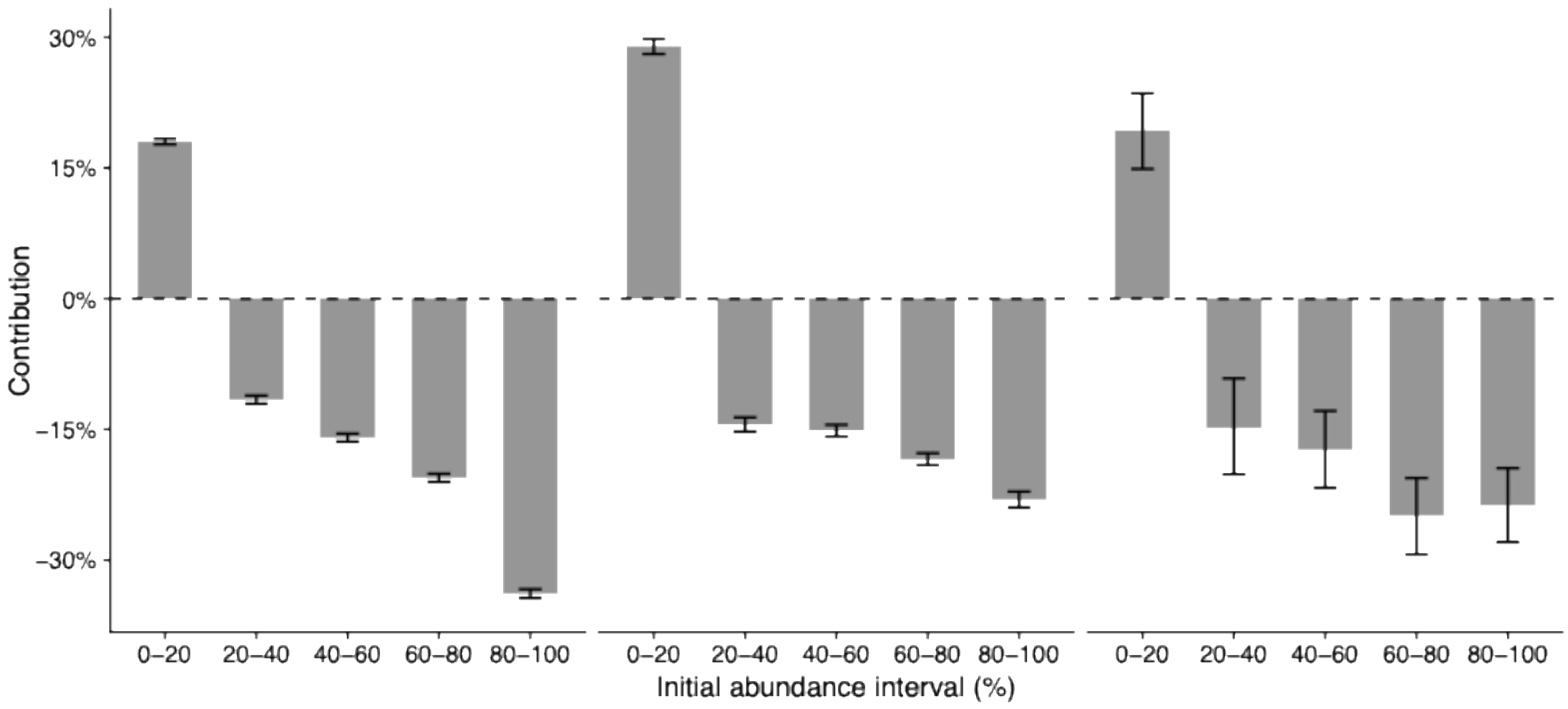

L671: I can’t see baseline temperature in Extended Fig 6.

RESPONSE: The reference to baseline temperature was a remnant from an earlier version of the analysis. We have removed this sentence from the revised manuscript, as Extended Fig. 6 does not include baseline temperature.

L694-695: ‘applied the same methodology as above’ – Two alternative methodologies for assigning abundance intervals (five evenly space bins v quartiles) have been described. Which one is this referring to?

RESPONSE: We clarify that “the same methodology as above” refers to the use of five evenly spaced bins. The revised sentence (Line 789–792) reads: “defined initial abundance as the mean log_{10}-transformed abundance over the first three/five years of each assemblage, then classified species into initial-abundance intervals using five evenly spaced bins to estimate trends (Extended Data Fig. 5).”

L742/Ext Fig 1: Data is mostly from ‘western’/northern countries – highly representative of modified ecosystems. How is this likely to affect your conclusions? Could the positive relationship between abundance trends and climate change/human impacts (Ext Dat Fig 6, Marine) be due to recent population recoveries in these regions (post historical declines) co-occurring with ongoing global

warming/human modification?

RESPONSE: We agree that the predominance of data from western/northern countries is an important limitation of the BioTIME dataset, and it may affect the generality and interpretation of our conclusions because these regions often represent highly modified ecosystems with monitoring histories and management contexts. We now state this explicitly in the Discussion (Line: 315–318 and 389–393).

We also agree that the reviewer's alternative interpretation is plausible: the positive relationship in the marine results between abundance trends and climate change/human impacts could reflect recent population recoveries in these regions following historical declines that co-occur in time with ongoing warming and human modification, rather than a direct positive response to contemporary change. We have added this as an alternative explanation in the Discussion (Line: 318–321 and 389–393).

L756: There don't appear to be any neutral trends presented in this figure, no credible intervals overlap 0.

RESPONSE: We have removed "neutral" to align with the figure' s results.

L760/Ext Fig 3: I think the approach here for assigning abundance classes is more robust than the one presented in the main text.

RESPONSE: We agree that there are good reasons to choose both the 1- and 5-year window for assigning abundance classes. Here, we decided to retain the first-year abundance approach in the main text because it provides a clear temporal baseline that is conceptually distinct from the trend data. Using a five-year average would incorporate part of the time series into the classification criterion, potentially introducing circularity. Nevertheless, the consistency between the two approaches (main text vs. Extended Fig. 3) demonstrates that our conclusions are robust to the choice of classification method.

L782: You highlight a more conservative correction approach, but do not use it. Why not? In many realm: taxon combinations, greater regression to the mean is seen under 3-year truncation than 1-year. Does this not suggest that using correction factors based on 1-year truncation is insufficient to address the issues of regression to the mean?

RESPONSE: We selected the 1-year truncation approach to balance the need to address regression to the mean effects while maintaining adequate sample sizes across realm-taxon combinations.

To directly address the reviewer's concern, we conducted analyses using the 3-year truncation approach. As shown in the figure provided below, our main conclusions remain consistent under this more conservative correction, suggesting that our findings are robust to the choice of truncation period. We have added the results of this 3-year truncation sensitivity analysis to the Supplementary Table (Line 665).

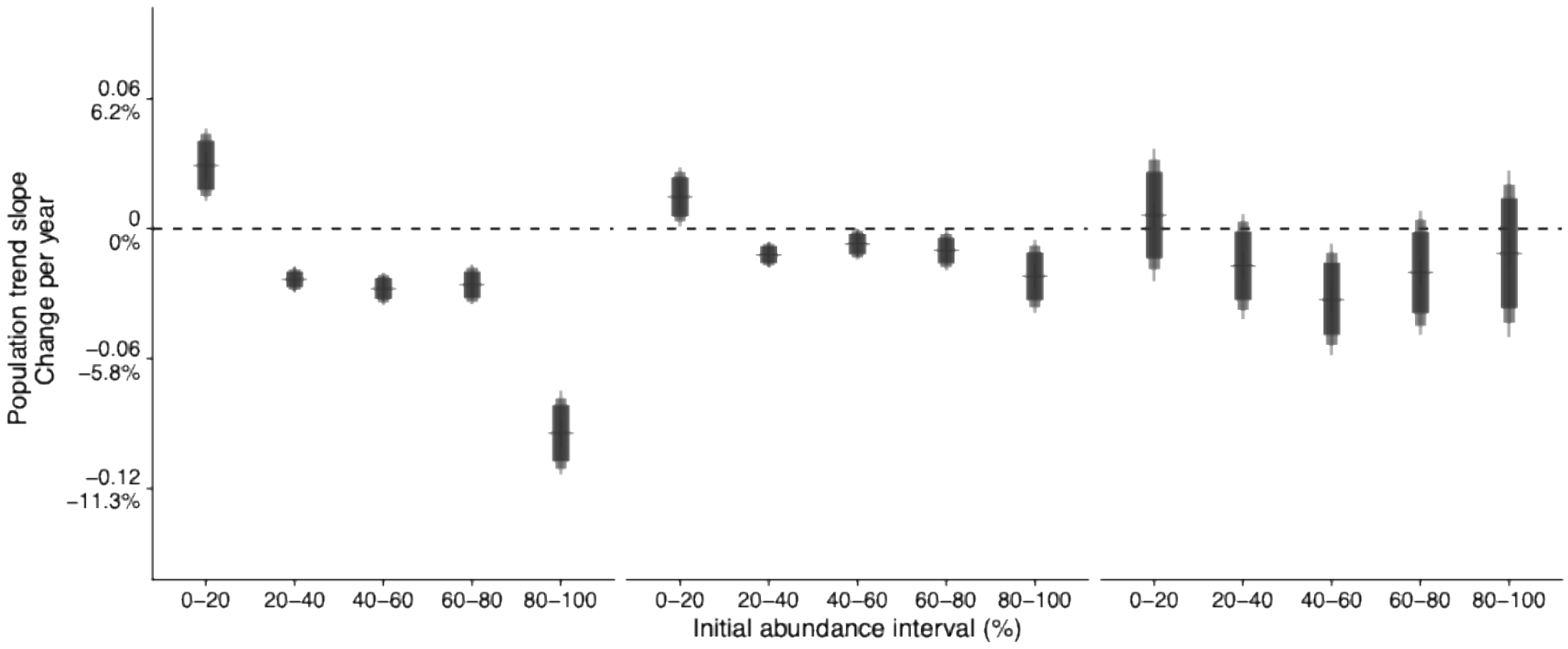

L808/Ext Fig 7: Excluding species with 0s in the start year completely removes the increases of rare species presented in the main results. I think this undermines the robustness of your main conclusions – at least with regards to rare species.

RESPONSE: We acknowledge that excluding species with leading zeros does remove the positive trend for rare species. However, we view this as demonstrating the transparency of our analytical approach rather than undermining our conclusions.

As discussed in our response to MAJOR CONCERNS 1, we recognize that both analytical approaches—including or excluding time-series with leading zeros—carry their own biases and represent valid methodological choices. Many studies would present only one approach that supports their preferred narrative. Instead, we deliberately present both analyses (main results and Extended Data Fig. 3) to provide full transparency about how this methodological decision influences the results, particularly for rare species.

We indicate our preferred approach (including leading zeros) with clear justification based on ecological reasoning, while also acknowledging that alternative perspectives exist. We believe this transparency allows readers and the scientific community to make informed interpretations of our findings and represents a more rigorous approach to addressing methodological uncertainty.

Reviewer #3 (Remarks to the Author)

I co-reviewed this manuscript with one of the reviewers who provided the listed reports. This is part of the Nature Communications initiative to facilitate training in peer review and to provide appropriate recognition for Early Career Researchers who co-review manuscripts.

RESPONSE: We thank Reviewer #3 for co-reviewing our manuscript. We appreciate your time and effort in evaluating our work, which has helped improve the quality of our manuscript.

Reviewer Comments & Author Response

Reviewer Reports for the First Version:

Reviewer #1 (Remarks to the Author):

The manuscript is much improved, and the authors have conducted very comprehensive sensitivity analyses to address the reviewer comments.

I just have a few remaining queries below.

Line 38 – maybe change to “Rapid biodiversity change” as biodiversity change in some form is a constant.

Line 344 – I can only see “non-climatic anthropogenic drivers” defined in the method section. It needs to be explained on first use. Here it makes it sound like they are basically land-use change but line 774 lists a far longer array. Many of these are hard to measure and the data used is derived from relatively broad scales in some cases. This may be another reason for the discrepancies reported. This is already alluded to by “unmeasured pressures” in line 343. It may not be that they are unmeasured as such, but the data quality available is limited.

Line 382 – BioTIME 2.0

Line 627 to 629 – Sorry, I am still not completely following the rarefaction. If the minimum annual sampling effort was three plots for example does this mean that for a given iteration the same three plots are used in each year? But then I do not understand how multiple iterations can be performed as at least 1 year only has three plots (which must be used for each year). If, however, three random plots are chosen each year then this seems counter productive as it is adding to spatial variability. 96km2 is huge for many of the taxa under consideration. Is this only applied in situations when site fidelity is already low?

Line 695 – I think the main caveat of not including spatial and phylogenetic structures into the analysis is that the results are only representative of the sample not the statistical population (global local assemblages across taxa) that you are aiming to estimate for. This now appears to be clearly mentioned in the text with the discussion of data representativeness, and I can see the computational and technical challenges of including space and phylogeny.

Line 855 to 856 _ I am not sure that point size is really visible here given the overlap of points in most regions. Showing this in another way or another panel may be better.

Line 914 – could the original (all time series boxes) be added in a different colour to make it easy to quickly see the difference.

Reviewer #2 (Remarks to the Author):

This review was written by the co-reviewer, the other co-reviewer was unable to read the manuscript.

I thank the authors for taking the time to respond to our concerns and comments. I believe the authors adequately address most of our concerns (e.g. allocation of abundance bins and statistical models), and adequately present the alternative considerations of initial zeros. However, there are still a few points that I would like to see addressed.

On the title

These analyses are of local assemblages, I would use that in the title rather than global. As the authors state, their analysis is on ‘within-assemblage dominance hierarchies and community reorganisation’, this feels like a local (assemblage-level), rather than global process. I would also be cautious that this process does not seem to be occurring in the freshwater realm, with stable trends across all abundance bins in Fig 1.

A title more accurately representing the results would be desirable.

e.g. “Widespread declines of formerly common species reshape local assemblages over time”.

I think a heatmap/Sankey plot or similar presenting abundance bin transitions for each realm would be a nice addition to one of the main figures, this is a key result relating to ‘reorganisation’ of local assemblages. Currently Table S7 presents an overall transition matrix, however I believe a realm-specific version is needed to support Fig 1. You also do not describe your methods for this table. I realise it is a fairly straightforward approach, but I think including the methods is needed. In particular, your description of how you calculated abundance bins (based on your response, average relative abundance in first 5 v last 5 years) differs from the approach used to generate abundance bins in your main results (log(abundance + 1)) – ignoring the 5-year v 1-year

component. It’s hard to know how much this would influence abundance bin allocation, but I think being consistent in the use of linear v log scales would be preferable.

I am not sure the authors responses regarding not checking residuals for spatial and phylogenetic correlation stand up to scrutiny. Whilst I agree that such residual correlation structure may not affect the direction of their trend estimates, it could be affecting the uncertainty. As noted in Johnson et al., 2024, and in this paper’s introduction (L 50-52) accounting for spatial and phylogenetic structure in population abundance datasets, including BioTIME, can substantially increase uncertainty in trend estimates. Without an assessment of model residuals, readers cannot usefully conclude how valid the presented models are.

Furthermore, the authors claim in their response that within the main document (on L690) they acknowledge “that unmodelled phylogenetic dependence—where present—could lead to underestimated uncertainty and should be considered when interpreting our inference”. However, such an acknowledgment is missing from the main text.

Further line-specific comments

L 28: Initially common freshwater species do not demonstrate declining trends.

L 30: I think a “,” after included would be needed to make the sentence clearer.

L 140-141 (Fig 1c): Based on your updated methodology for calculating Pi (Lines 720-741), I don’t think this is estimating contribution to ‘total abundance change’. Your estimates and calculations remain on the log-scale, thus your measures, as stated in the methods correspond to the log of relative abundance change. Given that you then standardise this by time-series length, and average over time-series per bin, I don’t fully see the value of this figure – doesn’t it just largely reproduce your estimated average trends in Fig 1b but on a different scale (and including time-series-species random effects)?

L 238-9: Initially common freshwater species do not demonstrate declining trends. Rephrase to reflect “marine and terrestrial taxa” or similar.

L 243-248: This is not linked to the taxa-specific results and seems a bit out of place here. It relates to overall results.

L 720: I’m not sure your calculations for deltaN, Ci, Pi, relate to total abundance change. See previous comment relating to L140/Fig 1c.

Extended data fig 6: Without censoring, large uncertainty in trends of rare species is consistently present. This uncertainty substantially declines under 1-, or 3-year censoring. Why does this happen? I know these are longer time-series (20+ years) than in the main results, but the substantial change in model uncertainty seems surprising, especially when similar patterns are not seen to the same extent for the other abundance intervals.

Finally, in their response, the authors claim that under-correction of regression to the mean is conservative. Unless I have misunderstood the methodology, I believe the opposite is true in relation to their results. A smaller magnitude of adjustment would, I expect, lead to the retention of trend estimates that are further from zero than they should be if properly accounting for regression to the mean, not the other way around.

Reviewer #3 (Remarks to the Author):

I co-reviewed this manuscript with one of the reviewers who provided the listed reports. This is part of the Nature Communications initiative to facilitate training in peer review and to provide appropriate recognition for Early Career Researchers who co-review manuscripts.

Author Response to First Version:

Reviewer #1 (Remarks to the Author):

The manuscript is much improved, and the authors have conducted very comprehensive sensitivity analyses to address the reviewer comments.

I just have a few remaining queries below.

Line 38 – maybe change to “Rapid biodiversity change” as biodiversity change in some form is a constant.

RESPONSE: We have revised this sentence as recommended, updating it to “Rapid biodiversity change is a defining feature of the Anthropocene[1]” (Line 43).

Line 344 – I can only see “non-climatic anthropogenic drivers” defined in the method section. It needs to be explained on first use. Here it makes it sound like they are basically land-use change but line 774 lists a far longer array. Many of these are hard to measure and the data used is derived from relatively broad scales in some cases. This may be another reason for the discrepancies reported. This is already alluded to by “unmeasured pressures” in line 343. It may not be that they are unmeasured as such, but the data quality available is limited.

RESPONSE: We agree with the reviewer’s comment. We have added a brief definition of “non-climatic anthropogenic drivers” at first mention (Line 112), and revised the wording from “unmeasured pressures” to accurately reflect limited data quality and coarse scale as the underlying reason for the observed discrepancies (Line 376).

Line 382 – BioTIME 2.0

RESPONSE: Done

Line 627 to 629 – Sorry, I am still not completely following the rarefaction. If the minimum annual sampling effort was three plots for example does this mean that for a given iteration the same three plots are used in each year? But then I do not understand how multiple iterations can be performed as at least 1 year only has three plots (which must be used for each year). If, however, three random plots are chosen each year then this seems counter productive as it is adding to spatial variability. 96km2 is huge for many of the taxa under consideration. Is this only applied in situations when site fidelity is already low?

RESPONSE: Thanks for your consideration. We have revised the Methods section (Line 711) accordingly and respond to each point below.

Regarding the rarefaction procedure, we follow the sample-based rarefaction workflow of Antão et al. (2020): in each of 199 iterations, every year within a time series is independently and randomly subsampled to the minimum annual sample size; the year already at the minimum is retained in full. Variation across iterations therefore arises solely from years exceeding the minimum, and the median across 199 iterations is taken for each metric. Because all subsampling is confined to the same study and same 96-km^2 grid cell, no cross-spatial variability is introduced; furthermore, taking the median across iterations averages out any within-cell spatial heterogeneity rather than amplifying it.

Regarding the 96-km^2 grid (established as the BioTIME standard by Blowes et al., 2019; Dornelas et al., 2025), its sole purpose is to standardize spatial extent across studies with highly variable original footprints. It is applied uniformly to all studies, but has no practical effect on fixed-site studies, whose samples already fall within a single cell by default.

Line 695 – I think the main caveat of not including spatial and phylogenetic structures into the analysis is that the results are only representative of the sample not the statistical population (global local assemblages across taxa) that you are aiming to estimate for. This now appears to be clearly mentioned in the text with the discussion of data representativeness, and I can see the computational and technical challenges of including space and phylogeny.

RESPONSE: We thank the reviewer for this precise framing of the issue. We agree that the primary limitation of omitting explicit spatial and phylogenetic structures is one of representativeness: our estimates describe the sample at hand rather than the broader statistical population of global local assemblages. We have strengthened the relevant passages in the Discussion to make this point more explicit (Line 428).

We further note that, as a supplementary check, a sensitivity analysis incorporating phylogenetic and spatial autocorrelation structures on a high-fidelity subset of the data confirmed that the main pattern of declining common species and increasing rare species remained consistent, albeit with wider credible intervals, which is consistent with the reviewer's characterization of the issue.

Line 855 to 856 – I am not sure that point size is really visible here given the

overlap of points in most regions. Showing this in another way or another panel may be better.

RESPONSE: We thank the reviewer for this suggestion. We have replaced the size aesthetic with a histogram inset in the bottom-left corner of the map, showing the distribution of time-series duration across all assemblages (see figure below).

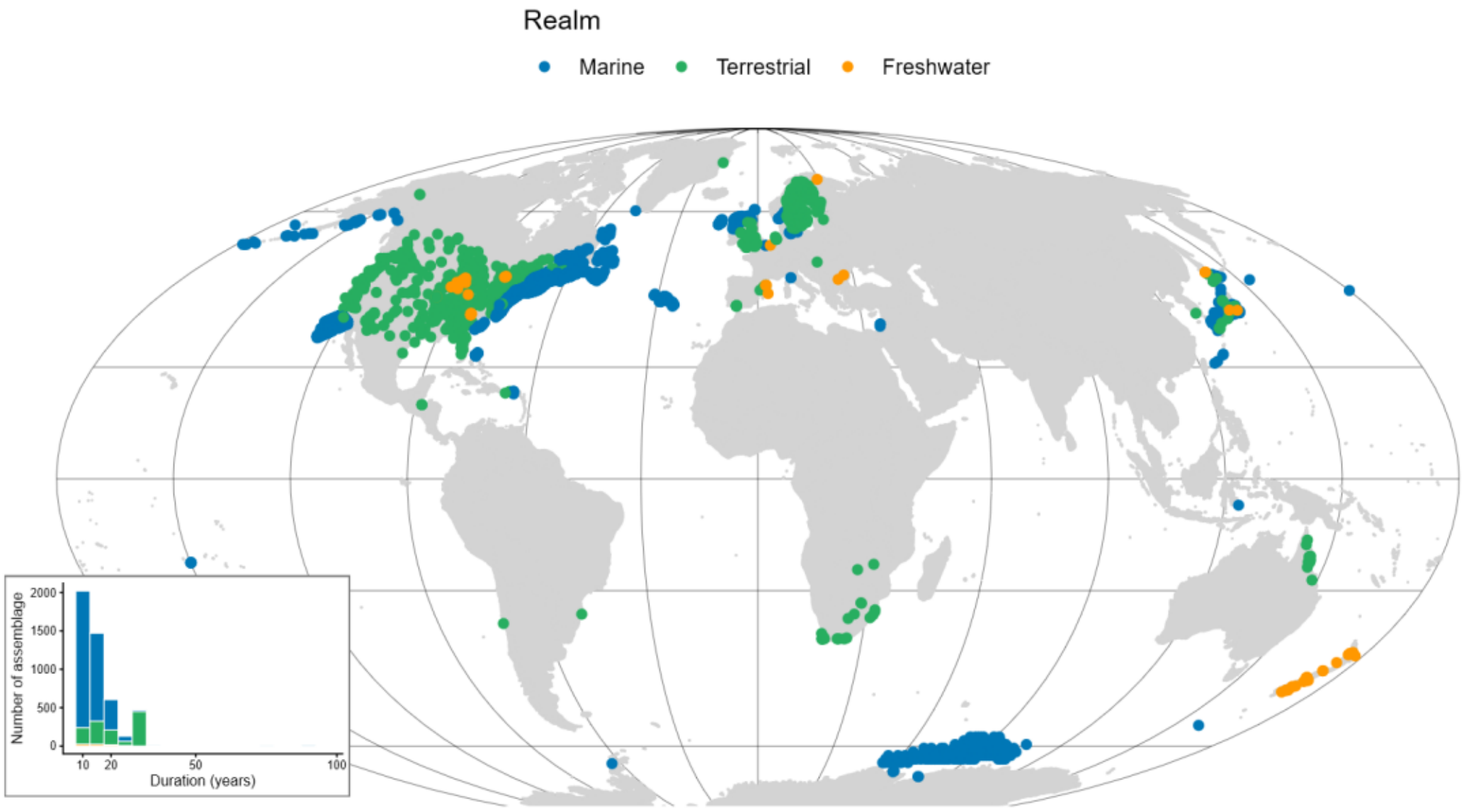

Line 914 – could the original (all time series boxes) be added in a different colour to make it easy to quickly see the difference.

RESPONSE: We thank the reviewer for this suggestion. We have added the full dataset results as an additional color category in Extended Data Fig. 8 (see figure below), enabling direct visual comparison with the high spatial-fidelity subset.

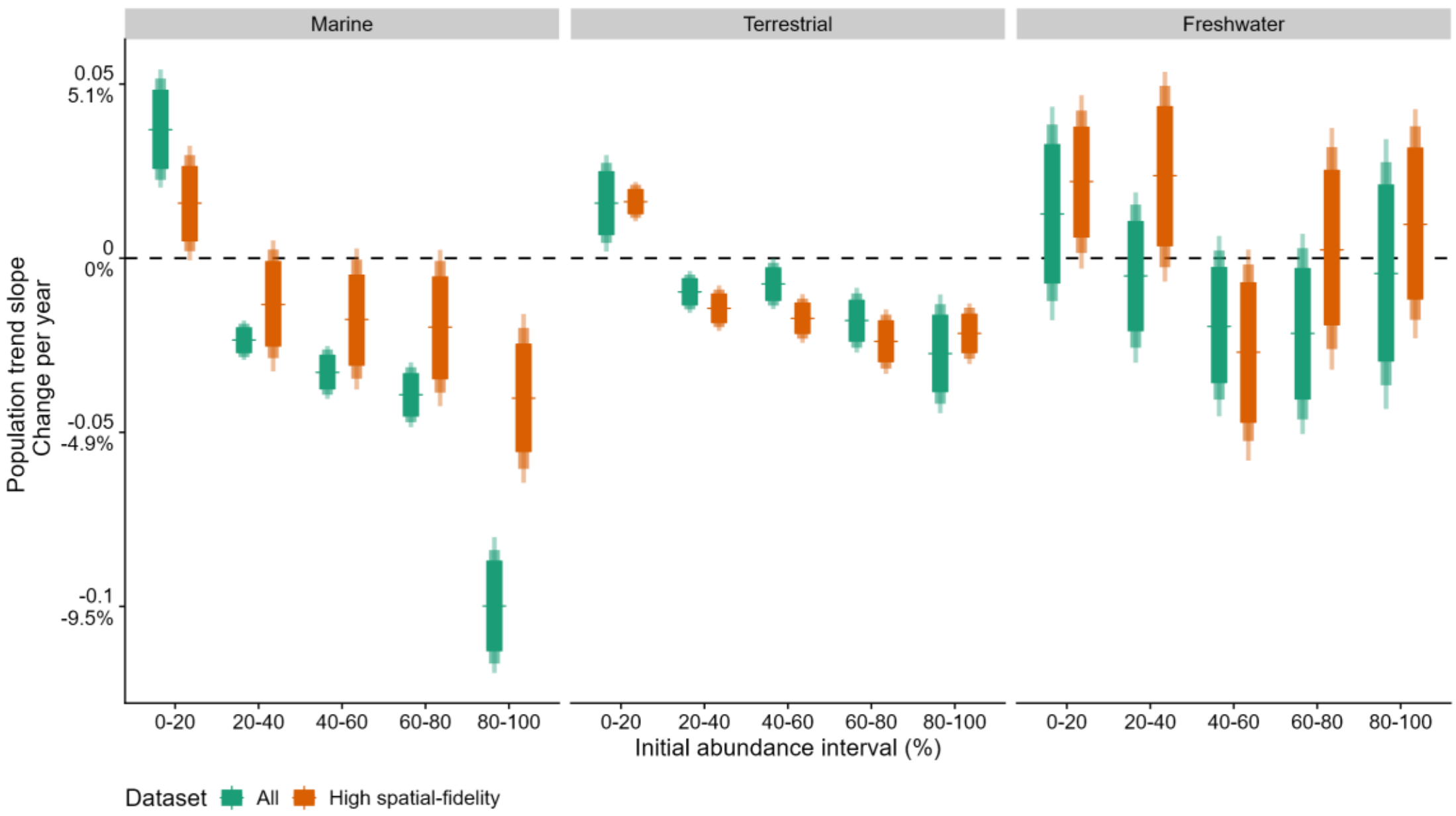

Marine
Terrestrial
Freshwater
Population trend slope
Change per year
0.05
5.1%
0
0%
-0.05
-4.9%
-0.1
-9.5%
0-20
20-40
40-60
60-80
80-100
Initial abundance interval (%)
Dataset
All
High spatial-fidelity

Reviewer #2 (Remarks to the Author):

This review was written by the co-reviewer, the other co-reviewer was unable to read the manuscript.

I thank the authors for taking the time to respond to our concerns and comments. I believe the authors adequately address most of our concerns (e.g. allocation of abundance bins and statistical models), and adequately present the alternative considerations of initial zeros. However, there are still a few points that I would like to see addressed.

On the title

These analyses are of local assemblages, I would use that in the title rather than global. As the authors state, their analysis is on 'within-assemblage dominance hierarchies and community reorganisation', this feels like a local (assemblage-level), rather than global process. I would also be cautious that this process does not seem to be occurring in the freshwater realm, with stable trends across all abundance bins in Fig 1.

A title more accurately representing the results would be desirable.

e.g. "Widespread declines of formerly common species reshape local assemblages over time".

RESPONSE: We thank the reviewer for these suggestions. We have made two revisions accordingly.

First, we have adopted the reviewer's suggested title.

Second, we acknowledge that the pattern of disproportionate common species decline was not evident in freshwater assemblages and have made this explicit in the manuscript. We revised the relevant sentences to read:

Line 30 (Abstract): We found consistently stronger declines among formerly common species through time in marine and terrestrial assemblages, while freshwater assemblages showed no clear directional pattern across abundance classes

Line 244 (Results): Despite these differences in total abundance trends among taxa, we found similar shifts in the strength of declines among species in different abundance categories for most taxa in marine and terrestrial realms (previously "across all realms and taxa").

Line 157 (Results): Both marine and terrestrial systems showed differences in

population change rates depending on initial abundance, while freshwater showed no such variation, though the limited number of freshwater studies (n = 66) may have constrained our ability to detect patterns.

I think a heatmap/Sankey plot or similar presenting abundance bin transitions for each realm would be a nice addition to one of the main figures, this is a key result relating to 'reorganisation' of local assemblages. Currently Table S7 presents an overall transition matrix, however I believe a realm-specific version is needed to support Fig 1. You also do not describe your methods for this table. I realise it is a fairly straightforward approach, but I think including the methods is needed. In particular, your description of how you calculated abundance bins (based on your response, average relative abundance in first 5 v last 5 years) differs from the approach used to generate abundance bins in your main results (log(abundance + 1)) – ignoring the 5-year v 1-year component. It's hard to know how much this would influence abundance bin allocation, but I think being consistent in the use of linear v log scales would be preferable.

RESPONSE: We thank the reviewer for these suggestions and have made the following revisions.

Following the reviewer's recommendation, we have replaced the original Table S7 with realm-specific abundance bin transition heatmaps, now presented as Fig. 1c. A detailed description of the procedure has been added to the Methods section ("Abundance bin transition analysis"). The abundance bin allocation has been revised to ensure full consistency with the main analysis: species abundances are now $\log_{10}$-transformed and normalized by the maximum $\log_{10}$-transformed abundance within each assemblage, using data from the first and last survey years rather than the averaged first and last five years used in the original table. In addition to the five equal-width non-zero intervals used in the main population trend analysis (>0–20%, 20–40%, 40–60%, 60–80%, and 80–100%), we introduced a sixth interval for zero-abundance observations (i.e., species absent in a given survey year). This distinction was necessary because species absent at the start that later colonized the assemblage differ fundamentally in expected trend direction from species that were present but rare, a point that proved central to interpreting trends in the rarest category (see Line 198–223).

The revised results are broadly consistent with the patterns reported in the original table but provide additional insight. Across all realms, initially common

species (80–100% bin) showed substantial displacement from their starting interval: only 23.0% of such species in marine, 55.8% in terrestrial, and 49.2% in freshwater assemblages remained in the highest abundance bin by the end of the time series. The inclusion of the zero-abundance interval also revealed that approximately 70–80% of colonizing species across realms were highly transient, going locally extinct before the final observation, consistent with the well-documented distinction between core and transient species in ecological assemblages.

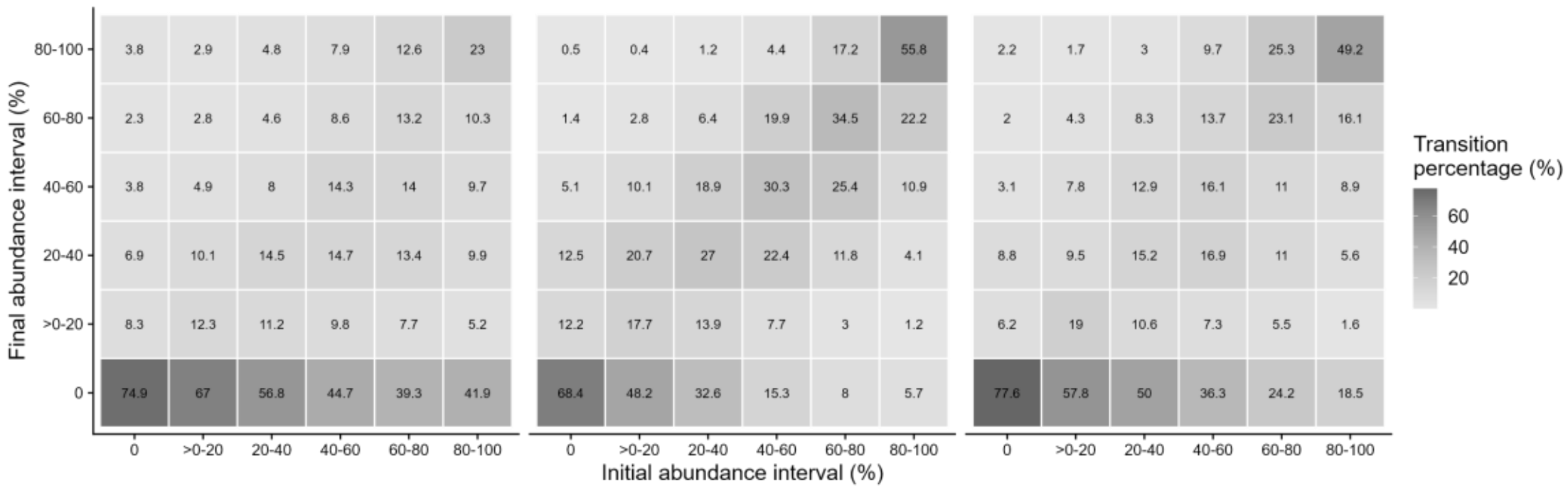

I am not sure the authors responses regarding not checking residuals for spatial and phylogenetic correlation stand up to scrutiny. Whilst I agree that such residual correlation structure may not affect the direction of their trend estimates, it could be affecting the uncertainty. As noted in Johnson et al., 2024, and in this paper's introduction (L 50-52) accounting for spatial and phylogenetic structure in population abundance datasets, including BioTIME, can substantially increase uncertainty in trend estimates. Without an assessment of model residuals, readers cannot usefully conclude how valid the presented models are.

Furthermore, the authors claim in their response that within the main document (on L690) they acknowledge "that unmodelled phylogenetic dependence—where present—could lead to underestimated uncertainty and should be considered when interpreting our inference". However, such an acknowledgment is missing from the main text.

RESPONSE: We thank the reviewer for this careful scrutiny and agree that the omission of spatial and phylogenetic correlation structures is more consequential for uncertainty estimation than for the direction of trend estimates. We also apologize for the inconsistency between our previous response and the main text: upon re-examination, we confirm that the acknowledgment referenced was not present in the submitted manuscript,

and we have now added it explicitly (Line 780).

To address the concern about underestimated uncertainty more directly than residual diagnostics alone would allow, we conducted a sensitivity analysis in which both a phylogenetic correlation matrix and a spatial autocorrelation structure were incorporated into the model, following the approach of Johnson et al. (2024). Given the substantial increase in Negative Binomial model complexity, this was feasible only for the high-fidelity subset of our dataset (76 studies, Supplementary Table 9), which spans comparable taxonomic groups and geographic regions to the full dataset.

The results are presented in Figure S9 below, and confirmed the reviewer's concern: credible intervals were appreciably wider under this specification. For instance, the terrestrial 0–20% abundance interval shifted from strong evidence of increase (1.63%, 95% CRI: 1.07, 2.14%) to weak evidence of increase (1.92%, 80% CRI: 0.27, 3.59%), and the marine 20–40% interval shifted from weak evidence of decline (−1.32%, 80% CRI: −2.51, −0.08%) to moderate evidence of decline (−1.99%, 90% CRI: −3.65, −0.31%). We therefore acknowledge that the strength of evidence for individual estimates in the full-dataset models should be interpreted with appropriate caution, and that the presented credible intervals likely understate true uncertainty to some degree.

Nevertheless, the central pattern of increasing abundance among rare species and declining abundance among common species remained consistent across realms under this more complete specification, supporting the robustness of our main conclusions. We have updated the Methods and Discussion to reflect these findings (Line 899 and 441), including an explicit statement that unmodelled spatial and phylogenetic dependence may lead to underestimated uncertainty in the full-dataset results.

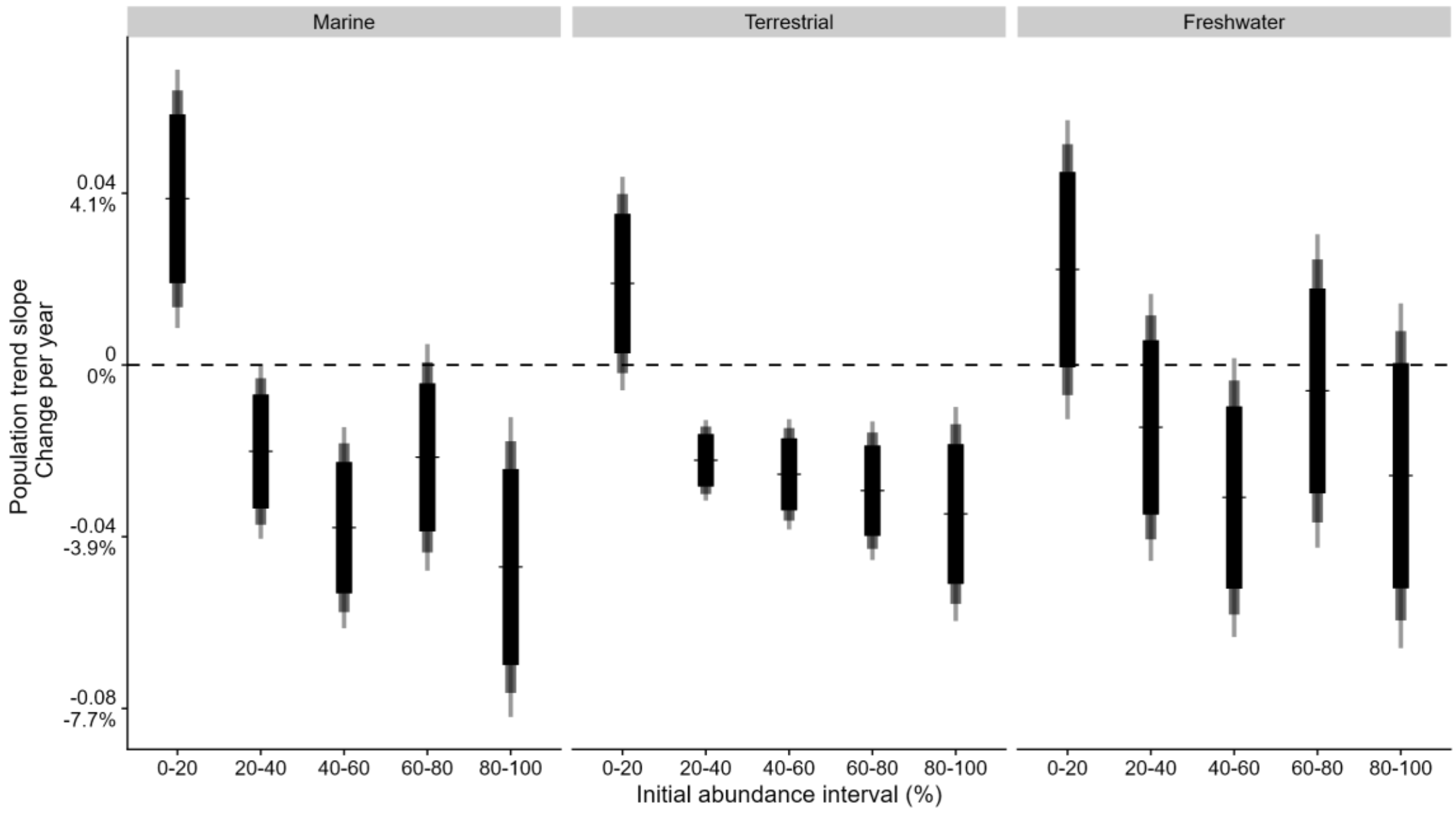

Further line-specific comments

L 28: Initially common freshwater species do not demonstrate declining trends.

RESPONSE: We agree. This has been addressed in our revision to the Abstract (Line 30), where we now read: "We found consistently stronger declines among formerly common species through time in marine and terrestrial assemblages, while freshwater assemblages showed no clear directional pattern across abundance classes."

L 30: I think a "," after included would be needed to make the sentence clearer.

RESPONSE: Done

L 140-141 (Fig 1c): Based on your updated methodology for calculating Pi (Lines 720-741), I don't think this is estimating contribution to 'total abundance change'. Your estimates and calculations remain on the log-scale, thus your measures, as stated in the methods correspond to the log of relative abundance change. Given that you then standardise this by time-series length, and average over time-series per bin, I don't fully see the value of this figure – doesn't it just largely reproduce your estimated average trends in Fig 1b but on a different scale (and including time-series-species random effects)?

RESPONSE: We thank the reviewer for this careful reading. We agree that

the label "contribution to total abundance change" was misleading given that all calculations remain on the log-scale, and that the figure largely reproduced the information already presented in Fig. 1b. We have therefore removed this figure entirely. In its place, the realm-specific abundance bin transition heatmaps (introduced in response to the previous comment) now occupy Fig. 1c, which we believe provides a more informative and complementary visualization of community reorganization alongside Fig. 1b.

L 238-9: Initially common freshwater species do not demonstrate declining trends. Rephrase to reflect "marine and terrestrial taxa" or similar.

RESPONSE: This has been revised to read: "Freshwater taxa again showed no clear increasing or decreasing patterns regardless of their initial abundance class." (Line 254), consistent with our earlier revisions clarifying that this pattern was not evident in freshwater assemblages.

L 243-248: This is not linked to the taxa-specific results and seems a bit out of place here. It relates to overall results.

RESPONSE: We agree that this text was misplaced within the taxa-specific results section. In the revised manuscript, we have relocated this content to follow the realm-level results in Fig. 1b, where it more naturally extends the discussion of community reorganization at the overall level (Line 198). Concurrently, as noted in our response to the previous comment, the underlying data have been updated to realm-specific figures derived from the log_{10}-transformed abundance bins consistent with the main analysis, and are now presented in the new Fig. 1c.

L 720: I'm not sure your calculations for deltaN, Ci, Pi, relate to total abundance change. See previous comment relating to L140/Fig 1c.

RESPONSE: As noted in our response to the earlier comment on L140/Fig. 1c, we agreed with the reviewer that these calculations did not appropriately represent total abundance change. The original Fig. 1c and all associated methods (the ΔN, C_i, and P_i calculations) have been removed from the manuscript entirely.

Extended data fig 6: Without censoring, large uncertainty in trends of rare species is consistently present. This uncertainty substantially declines under 1-, or 3-year censoring. Why does this happen? I know these are longer time-series (20+ years) than in the main results, but the substantial change in model uncertainty seems surprising, especially when similar patterns are not

seen to the same extent for the other abundance intervals.

RESPONSE: We apologize for this lack of clarity in the original manuscript. The unusually wide credible intervals for the 0–20% interval without censoring reflect instability in INLA model convergence. Unlike conventional MCMC approaches, INLA approximates posterior distributions without running chains, which makes standard convergence diagnostics unavailable. Following the approach of Johnson et al. (2024), we validated convergence by re-running each model 10 times, using the parameter modes from each prior run as initial values for the subsequent run. After applying this procedure, the models yielded stable estimates with substantially reduced uncertainty, consistent with the patterns observed across the other abundance intervals.

We also note that Extended Data Fig. 6 serves a specific and limited purpose in our analysis: these models are used solely to estimate the magnitude of the regression-to-the-mean (RtM) correction, for which only the mean trend estimates are required. The mean estimate for the 0–20% interval prior to re-running was 0.0403, compared with 0.0437 after obtaining stable convergence. This difference is relatively small, suggesting that this convergence issue likely had limited influence on the RtM correction factors applied in the main analysis.

Finally, in their response, the authors claim that under-correction of regression to the mean is conservative. Unless I have misunderstood the methodology, I believe the opposite is true in relation to their results. A smaller magnitude of adjustment would, I expect, lead to the retention of trend estimates that are further from zero than they should be if properly accounting for regression to the mean, not the other way around.

RESPONSE: We thank the reviewer for this important correction. We acknowledge that the reviewer is right: under-correction of RtM effects would result in trend estimates that remain more extreme than the true pattern, rather than being conservative with respect to our main conclusions. We apologize for the erroneous framing in our previous response.

Nonetheless, we maintain that using long time series (≥20 years) to derive correction factors is methodologically justified, following van Klink et al. (2024). While longer time series may yield smaller correction factors, they provide more stable and reliable estimates of the correction factor itself, as shorter series are subject to greater random fluctuation that would compromise the precision of the correction. The key trade-off is therefore

between reliability and potential underestimation of magnitude.

More substantively, Extended Data Fig. 6 provides evidence of the robustness of our conclusions to the magnitude of RtM correction. The progressive shrinkage of trend estimates toward zero with increasing truncation (0-, 1-, and 3-year censoring) demonstrates that stronger correction does attenuate the estimated declines of common species. Critically, however, the qualitative pattern—that initially common species decline more than rarer ones—persists consistently across all truncation scenarios. We therefore believe that while we cannot exclude the possibility that our estimates are slightly more extreme than the true effect, the core conclusion is unlikely to be an artifact of residual RtM effects.

Reviewer #3 (Remarks to the Author):

I co-reviewed this manuscript with one of the reviewers who provided the listed reports. This is part of the Nature Communications initiative to facilitate training in peer review and to provide appropriate recognition for Early Career Researchers who co-review manuscripts.

RESPONSE: We once again thank Reviewer #3 for their time and contribution to the peer review process. We appreciate their engagement with the manuscript as part of the Nature Communications Early Career Researcher co-review initiative, and are grateful for their role in helping to improve the quality of this work.
