# Supplementary material for: Widespread declines of formerly common species reshape local assemblages over time
Source: Nat Commun. 2026 Sep 12;17:10769. doi: 10.1038/s41467-026-77501-4 (PMC13661514; doi:10.1038/s41467-026-77501-4)
Supplement: Supplementary file 3 [file 41467_2026_77501_MOESM1_ESM.pdf]

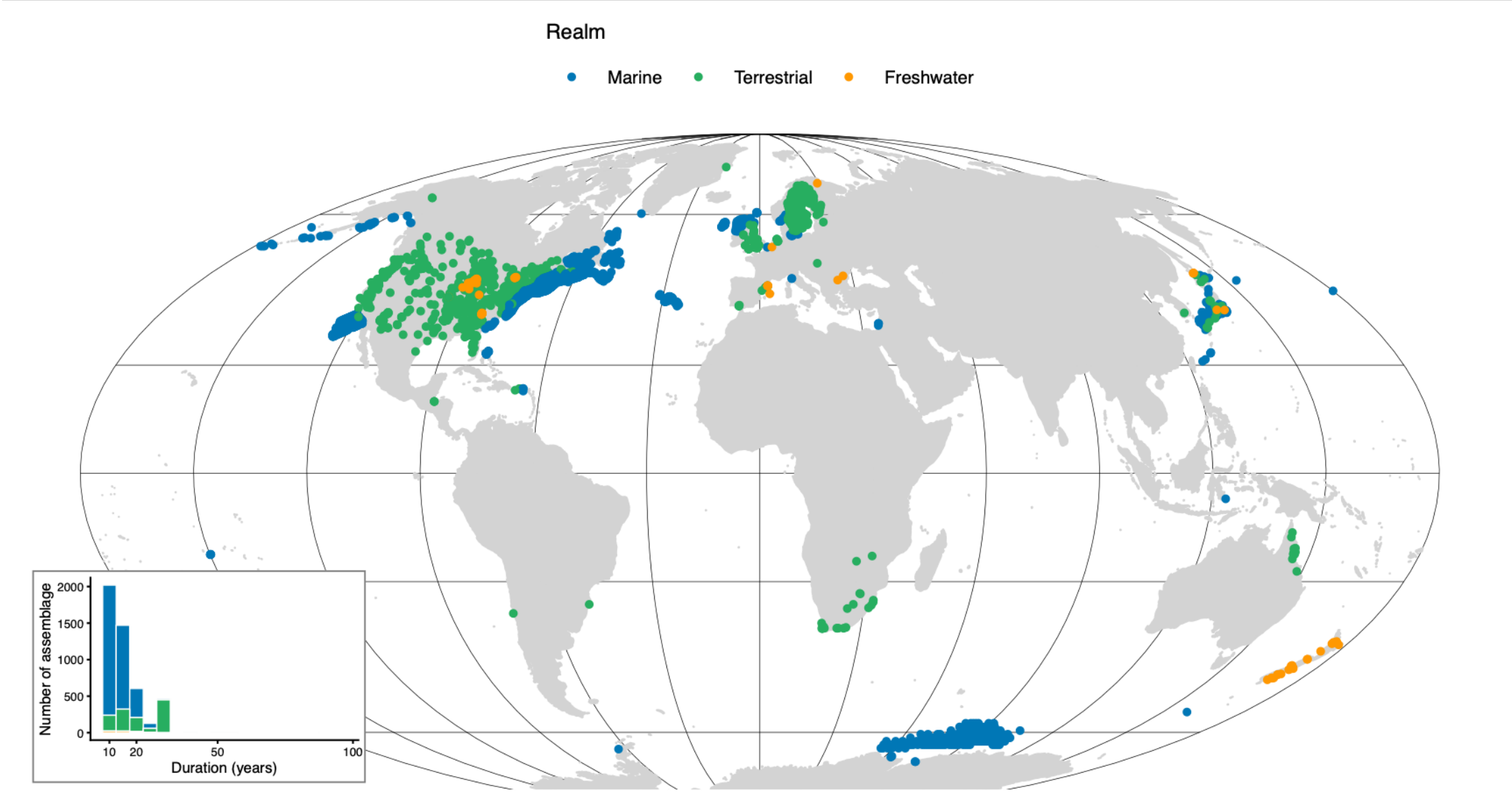

Supplementary Fig.1 | Geographic distribution of biodiversity time series. The map shows the locations of 4,748 assemblage time series across three realms (blue: Marine; green: Terrestrial; orange: Freshwater). The inset histogram (bottom left) shows the distribution of time-series duration (years) across all assemblages, colored by realm.

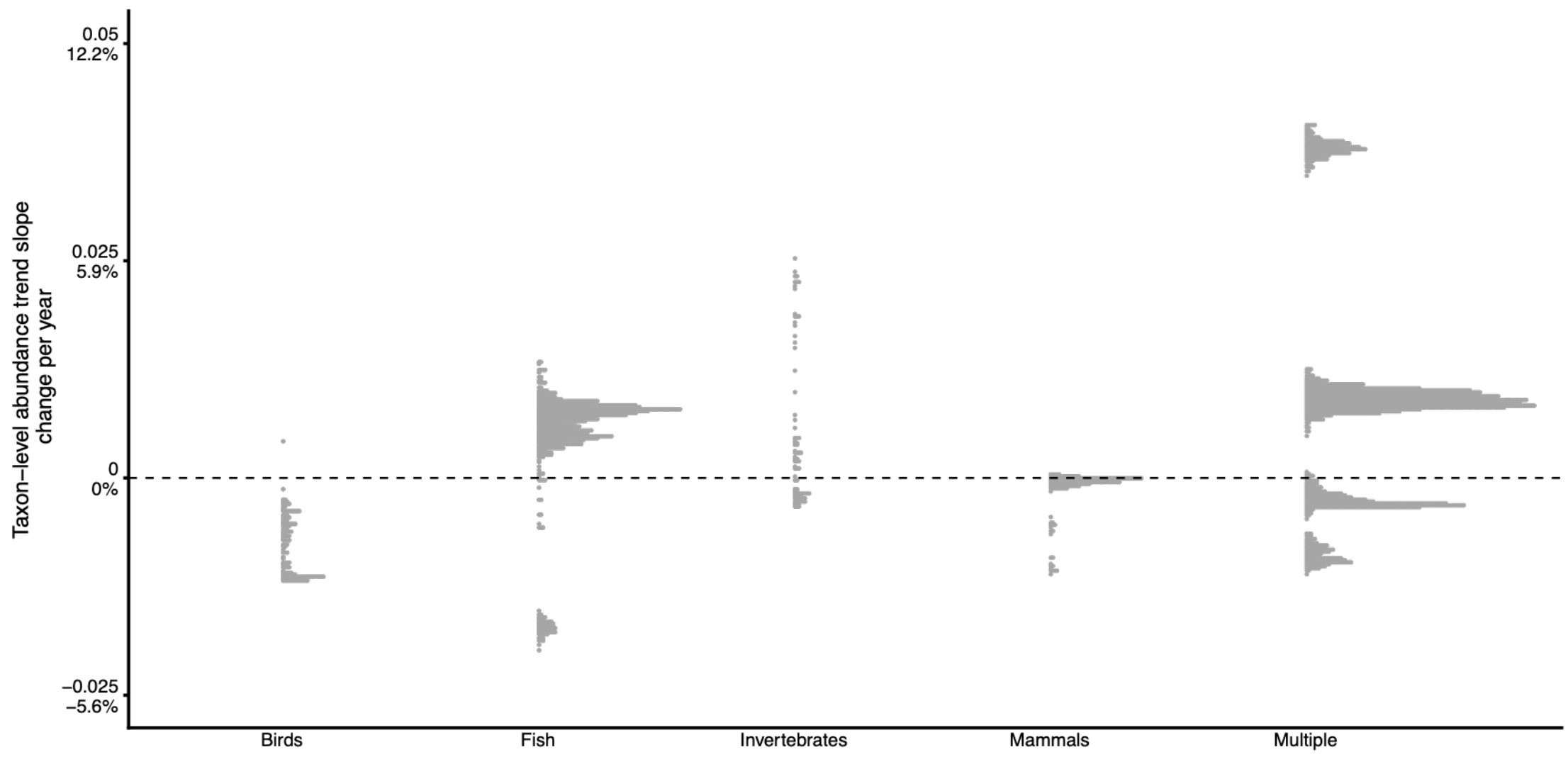

Supplementary Fig. 2 | Distribution of total abundance trend slopes by taxonomic group in marine assemblages. Each dot represents a single time series (*n* = 3,457 marine time series total); vertical position indicates the posterior mean abundance trend slope. Dashed line indicates zero trend. Negative slopes are most common in multi-taxa studies (Multiple category), which sample several taxonomic groups simultaneously within the same assemblage.

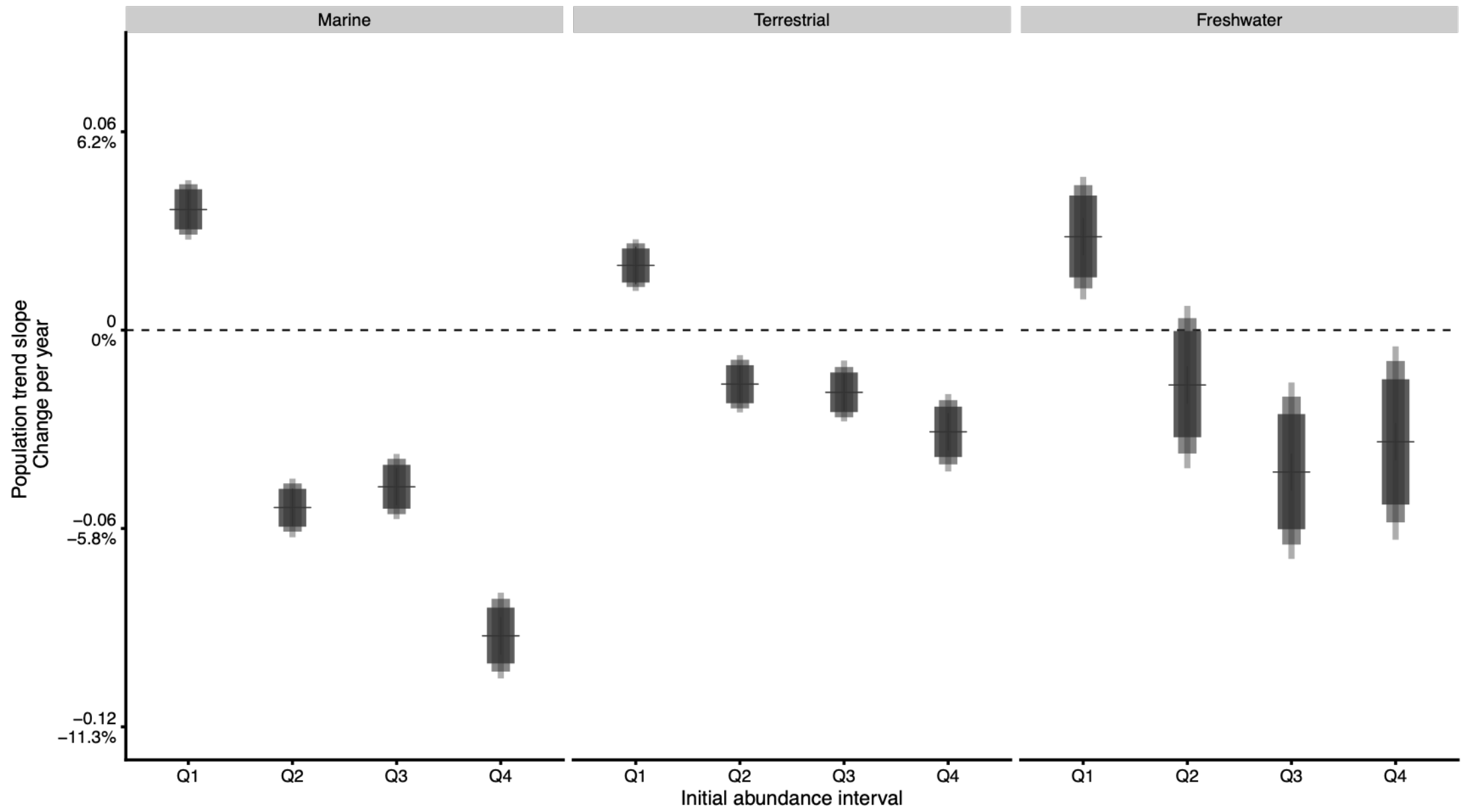

Supplementary Fig. 3 | Population trend estimates using quartile-based abundance classifications. Cross symbols indicate posterior means; thick, medium, and thin error bars represent 80%, 90%, and 95% CRI, respectively, for species grouped by abundance quartiles across marine, terrestrial, and freshwater realms. Dashed line indicates zero trend. Unlike the main analysis that used five equal-width intervals of $\log_{10}$-transformed abundance, this approach assigned species to four quartiles with approximately equal numbers of species–assemblage combinations. Results are qualitatively consistent with main analysis.

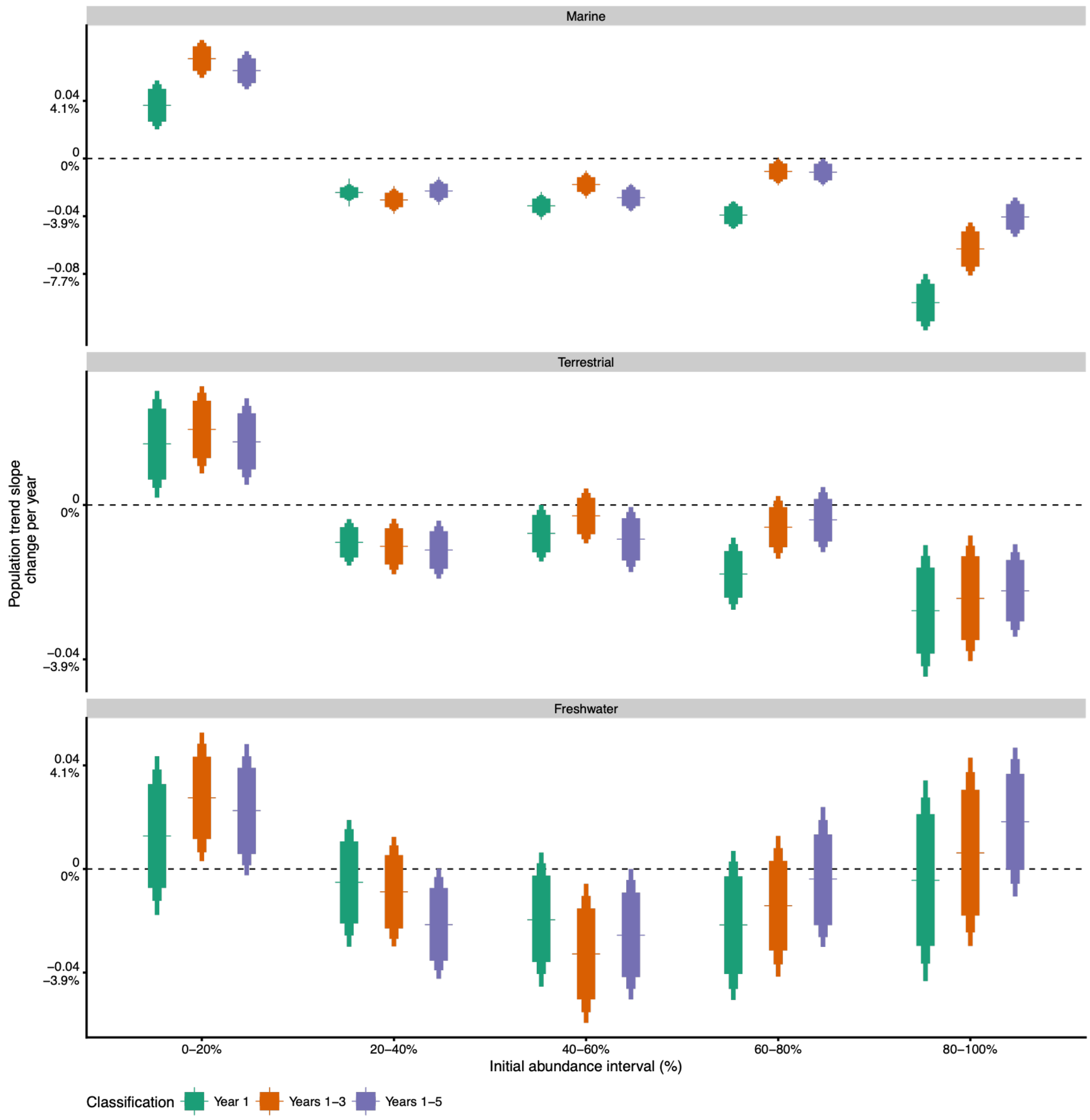

Supplementary Fig. 4 | Population trend estimates using multi-year baseline abundance definitions. Cross symbols indicate posterior means; thick, medium, and thin error bars represent 80%, 90%, and 95% CRI, respectively, for species grouped by initial abundance intervals across realms. Dashed line indicates zero trend. Species were classified based on mean log_{10}-transformed abundance from the first one, three, or five survey years. Colors indicate the baseline period used. Results are qualitatively consistent across all three definitions.

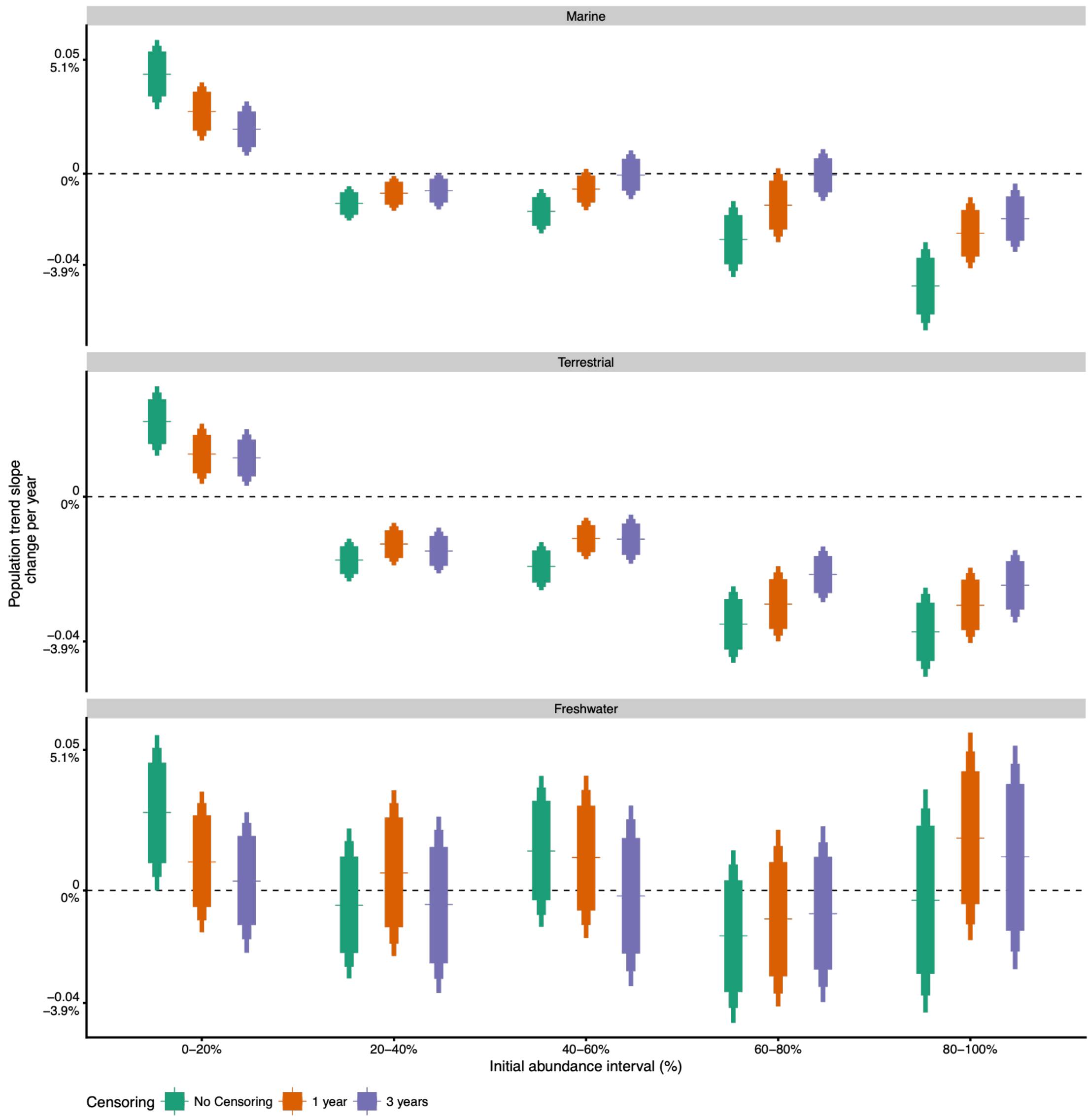

Supplementary Fig. 5 | Regression-to-the-mean correction under different time-series truncation scenarios. Cross symbols indicate posterior means; thick, medium, and thin error bars represent 80%, 90%, and 95% CRI, respectively. Colors indicate truncation scenario: no truncation, 1-year, and 3-year left-truncation. Dashed line indicates zero trend. Progressive shrinkage of estimates toward zero with increased truncation reflects the magnitude of regression-to-the-mean bias. Differences between the no-truncation and 1-year-truncation estimates were used as realm-specific correction factors in the main analysis. Numerical correction values are provided in Supplementary Data 4.

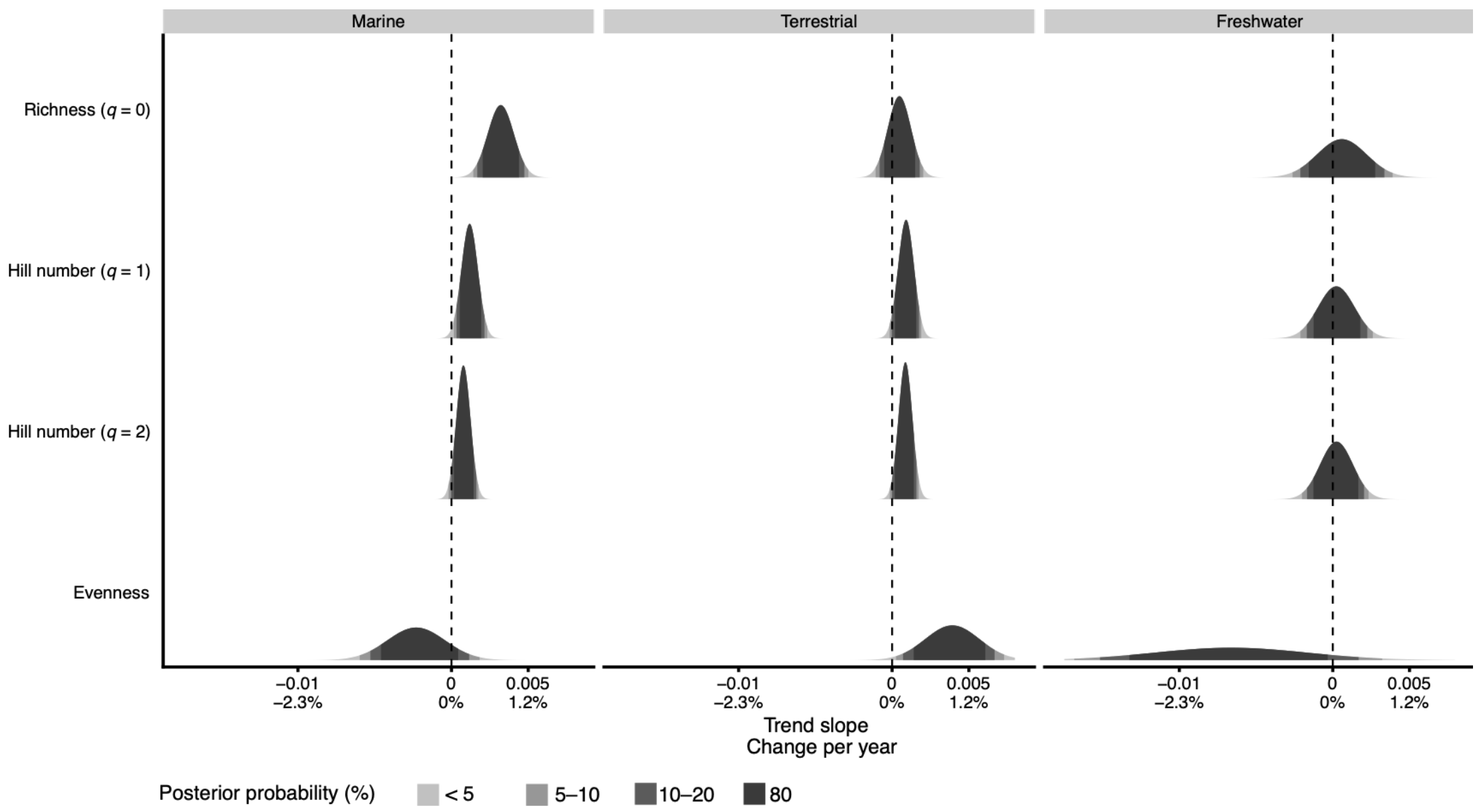

Supplementary Fig. 6 | Posterior distributions of biodiversity metric trends across realms. Density curves show the posterior probability distributions of annual trend slopes for species richness (q = 0), Hill number for Shannon diversity (q = 1), Hill number for Simpson diversity (q = 2), and Pielou's evenness, across marine, terrestrial, and freshwater realms. Shading represents 80%, 90%, and 95% CRI. Vertical dashed lines indicate zero change.

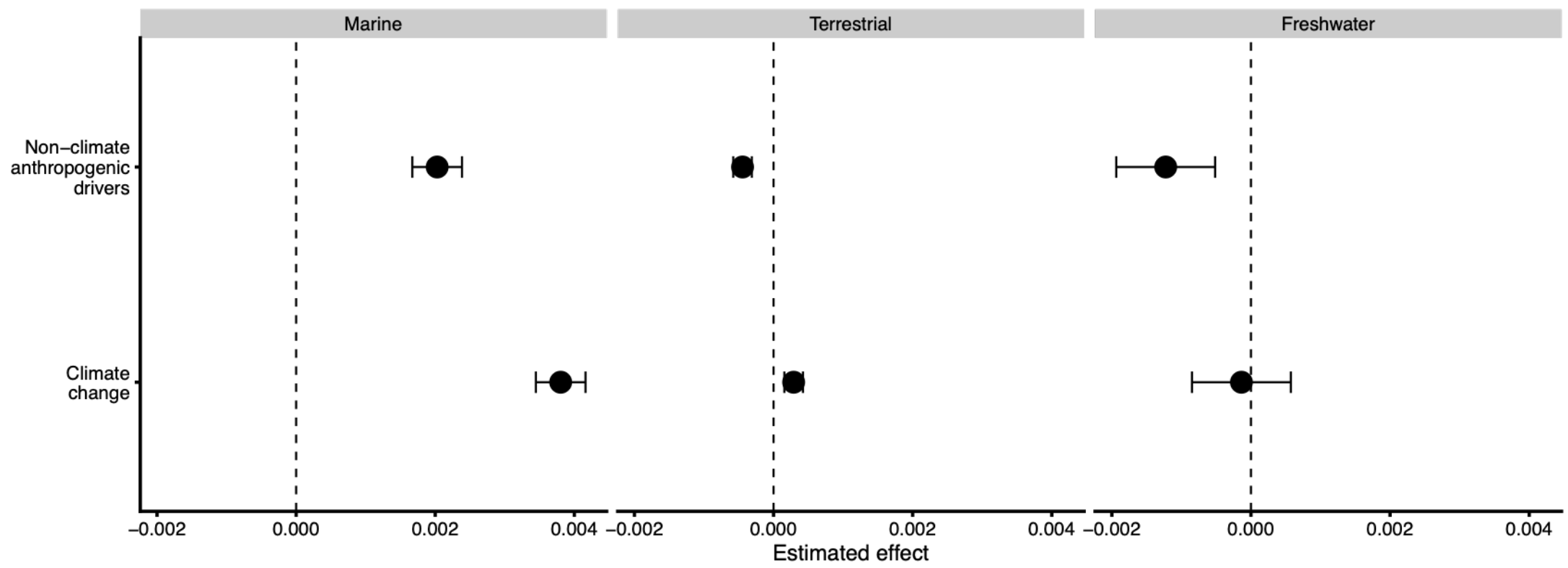

Supplementary Fig. 7 | Environmental correlates of total assemblage abundance change. Points represent standardized OLS regression coefficients; error bars show 95% confidence intervals from models relating time series-level total abundance change rates to temperature change and non-climatic anthropogenic drivers. Dashed line indicates zero trend. Models were fitted separately for each realm (Marine: *n* = 3,219; Terrestrial: *n* = 1,223; Freshwater: *n* = 66 assemblage time series). Positive coefficients indicate that higher values of the driver are associated with more positive abundance trends.

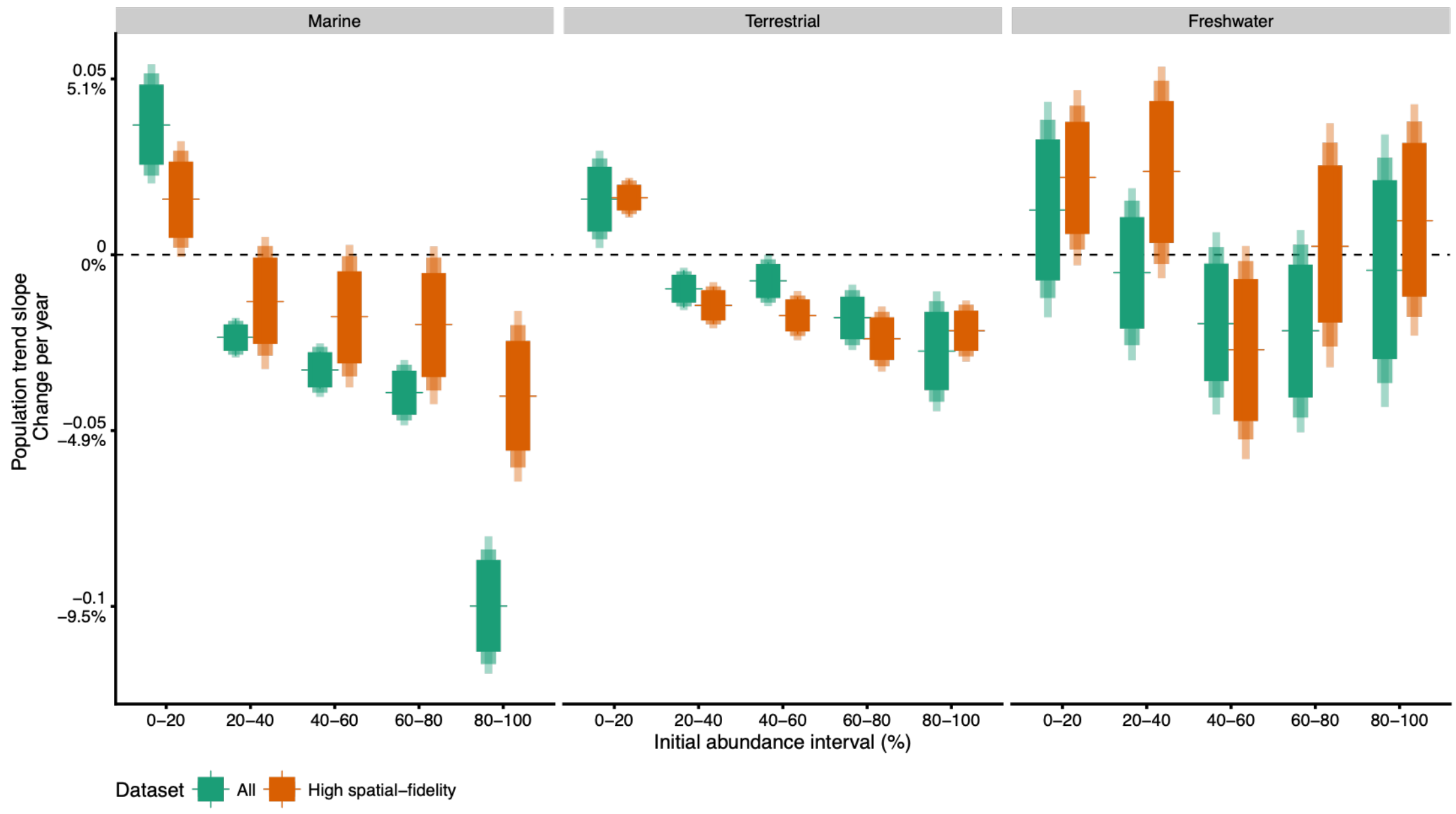

Supplementary Fig. 8 | Sensitivity analysis using only high spatial-fidelity studies. Cross symbols indicate posterior means; thick, medium, and thin error bars represent 80%, 90%, and 95% CRI, respectively, for species grouped by initial abundance intervals across realms. Colors distinguish results from all time series (All dataset) versus the high spatial-fidelity subset (*n* = 76 studies, 59% of total; studies with explicitly documented permanent or fixed sampling locations, see Supplement Data 9). Dashed line indicates zero trend. Results from the high spatial-fidelity subset are qualitatively consistent with the full dataset.

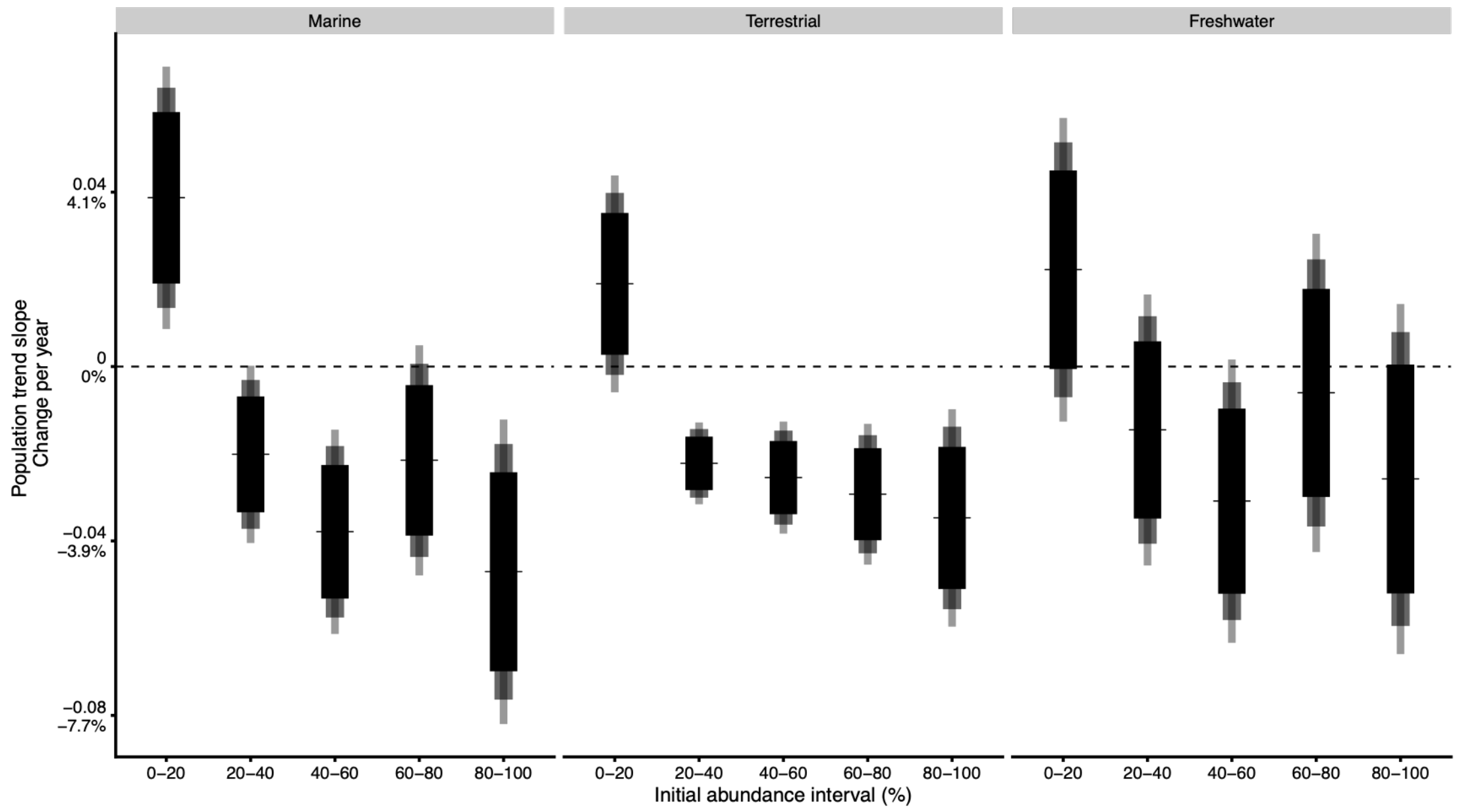

Supplementary Fig. 9 | Sensitivity analysis incorporating phylogenetic and spatial autocorrelation structures. Cross symbols indicate posterior means; thick, medium, and thin error bars represent 80%, 90%, and 95% CRI, respectively, for species grouped by initial abundance intervals across marine, terrestrial, and freshwater realms. Dashed line indicates zero trend. Models additionally incorporated a phylogenetic correlation matrix and a spatial autocorrelation structure, restricted to the high spatial-fidelity subset of studies. Compared with the main analysis, credible intervals are appreciably wider under this specification. The central pattern of stronger declines in initially common species remains qualitatively consistent with the main analysis across marine and terrestrial realms.
