# Supplementary material for: Widespread declines of formerly common species reshape local assemblages over time
Source: Nat Commun. 2026 Sep 12;17:10769. doi: 10.1038/s41467-026-77501-4 (PMC13661514; doi:10.1038/s41467-026-77501-4)
Supplement: Supplementary file 4 [file 41467_2026_77501_MOESM2_ESM.pdf]

Description of Additional Supplementary Files

File Name: Supplementary Data 1
Description: Summary of published studies reporting population trends in relation to initial species abundance, including study details, taxa, realms, and key findings. References for the included studies are provided in a separate tab.

File Name: Supplementary Data 2
Description: Total abundance trends across ecological realms. Mean annual rate (%) of change in total abundance for marine, terrestrial, and freshwater assemblages, derived from hierarchical Bayesian models.

File Name: Supplementary Data 3
Description: Population trends by initial abundance intervals across realms. Mean annual rate (%) of change for species grouped by initial relative abundance percentiles across marine, terrestrial, and freshwater ecosystems.

File Name: Supplementary Data 4
Description: Population trend estimates under no truncation, 1-year, and 3-year left-truncation, with realm-specific regression-to-the-mean correction factors derived from the difference between truncated and non-truncated slopes.

File Name: Supplementary Data 5
Description: Posterior mean total abundance trend estimates and credible intervals by taxonomic group and realm.

File Name: Supplementary Data 6
Description: Taxonomic patterns of population trends by initial abundance intervals across realms. Mean annual rate (%) of change for species grouped by taxonomic identity and initial relative abundance percentiles across marine, terrestrial, and freshwater ecosystems. Numbers in parentheses alongside Taxa labels indicate the number of species per taxonomic group, realm, and abundance interval.

File Name: Supplementary Data 7
Description: Standardized OLS regression coefficients and 95% confidence intervals for temperature change and nonclimatic anthropogenic drivers, by realm and initial abundance interval, corresponding to Fig. 4. Numbers in parentheses alongside Realm labels indicate the number of species per abundance interval and realm.

File Name: Supplementary Data 8
Description: WAIC values for Negative Binomial and Poisson model specifications, used to justify selection of the Negative Binomial framework.

File Name: Supplementary Data 9
Description: Classification of all 129 studies by spatial fidelity level (high, medium, low), including Study IDs and supporting metadata, used in the sampling site fidelity sensitivity analysis. Study ID numbers correspond to references available in the BioTIME 2.0 database.
